# Towards a theory for Earth’s albedo stability and hemispheric symmetry in the 21st Century

**DOI:** 10.1038/s43247-026-03817-0

**Published:** 2026-07-21

**Authors:** Daniel R. Feldman, Jake J. Gristey, Maria Z. Hakuba, Doug Hellinger, Samuel Kahn

**Affiliations:** 1 Earth and Environmental Sciences Area, Lawrence Berkeley National Laboratory, Berkeley, CA USA; 2 Cooperative Institute for Research in Environmental Sciences, University of Colorado, Boulder, CO USA; 3 NOAA Chemical Sciences Laboratory, Boulder, CO USA; 4 Laboratory for Atmospheric and Space Physics, University of Colorado, Boulder, CO USA; 5 Jet Propulsion Laboratory, California Institute of Technology, Pasadena, CA USA; 6 Santa Cruz Institute for Particle Physics, University of California-Santa Cruz, Santa Cruz, CA USA; 7 Research Fellow, University of California-Santa Cruz, Santa Cruz, CA USA

**Keywords:** Projection and prediction, Atmospheric science

## Abstract

The current scientific understanding of top-of-atmosphere globally-averaged albedo stability and hemispheric symmetry on interannual to centennial time-scales is largely phenomenological and diagnostic, leading to diverging albedo estimates over the 21^*st*^ Century. We present a framework for hypothesis testing and theory development, complemented by observations, data methods, and models, to advance towards a theory for these phenomena. We present the theory’s necessary features needed and then explore two hypotheses and their observational tests: that albedo is (1) constrained by invariant Earth system properties, or (2) maintained by cloud buffering. We argue that there is a supporting role for artificial intelligence (AI) methods that express the non-linear processes that underlie these observed phenomena. Additionally, we show that the response to and relaxation from major perturbations like stratovolcanic eruptions may help to falsify hypotheses. Finally multi-model ensemble experiments and observations can support this multi-pronged approach to advance towards a working theory for albedo.

## Abstract (web summary)

Artificial intelligence methods that capture key nonlinear processes can advance theory development for global albedo stability and symmetry, as shown by testing two competing hypotheses with observational data and multi model ensemble experiments.

## Introduction

The top-of-atmosphere albedo of Earth is central to setting the Earth system’s climate because imbalances in radiative energy necessarily produce climate variable responses in the Earth system^1,2^. Fundamentally, albedo is set by the absorption and reflection of solar radiation by the Earth’s surface and atmosphere, and this ratio of reflected to incoming shortwave radiation at the top-of-atmosphere is, globally and annually-averaged, approximately 29%^3^. To date, the scientific understanding of globally-averaged albedo is largely based on satellite instrument records. The foundation of the observational record comes from a robust, multi-decadal set of comprehensive, spatially and temporally-resolved measurements of top-of-atmosphere (TOA) reflected shortwave radiation (RSR) from a fleet of Clouds and the Earth’s Radiant Energy System (CERES) instruments that began circa 2000 and, as of this writing, is ongoing^4,5^ and will be continued by Libera^6–8^. Separate measurements of TOA solar insolation from the SOlar Radiation and Climate Experiment (SORCE) and the Total Solar Irradiance Spectrometer (TSIS)^9^ enable the calculation of albedo. From these and other satellite observations of RSR and other related quantities^10,11^, the scientific community has determined that even though top-of-atmosphere albedo varies dramatically in space and time, it has also exhibited two remarkable, but as yet unexplained, phenomena:

1. The globally-averaged albedo appears to be stable, or nearly stable, on multi-decadal time-scales^3,12–14^, with global-averages ranging from 25% to 33% on hourly to seasonal time-scales^15^, while remaining around 29% on interannual time-scales and exhibiting only a change from 29.4% to 28.7% from 2000 to 2025^16^.
2. The albedos of the northern and southern hemispheres are indistinguishable^3,17–22^, although there are initial indications that an asymmetry may be emerging^23^.

Regarding the first phenomenon, recent observational data are showing approximately a 1% decrease in globally-averaged albedo driven by reduced low-level clouds at low-latitudes^24^. However, the hemispheric symmetry of albedo has shown, to within observational uncertainty, no change^20^.

While the scientific community is able to decompose the spatial and temporal fluctuations in albedo and ascribe contributions from surface reflectivity, clouds, and aerosols, that decomposition has only been able to provide suggestions as to an explanation of these phenomena and whether or not these phenomena will persist into the future. Previous studies have sought to advance the understanding of these phenomena through simplified models and other conceptual approaches^3,19^.

It is natural to turn to climate models for predictions and understanding. While climate models simulate globally-averaged and hemispheric albedo, they report a wide range of albedos, and have been individually unable to reproduce the observed phenomenon of hemispheric symmetry in albedo^25^. Some of the range of results can be attributed to net-radiation tuning by which model developers adjust under-constrained processes that impact outgoing longwave radiation (OLR) and albedo^26–29^. It remains unclear how this tuning impacts the predictive value of albedo from models for future projections.

Instead of just relying on tuned climate models to make a prediction of changes in globally- or hemispherically-averaged albedo, there is a need for a systematic approach to uncovering the principles that underlie the observed phenomena. This approach must recognize the challenges presented in predicting albedo, the rich range of observational datasets that are, and are increasingly becoming, available to address the problem, and a framework for evaluating candidate explanations of the underlying principles to the extent that they can be confronted with observations and either be confirmed or falsified.

Therefore, this paper focuses on advancing approaches to the study of top-of-atmosphere albedo in the 21st Century and is organized as follows: (1) we discuss gaps between phenomenology and predictive understanding, (2) we discuss the necessary conditions for a theory of albedo predictability at hemispheric and planetary spatial scales and seasonal to interannual temporal scales, (3) we present two hypotheses to explain the albedo phenomena and walk through corresponding existing and future observational tests for these hypotheses, (4) we discuss the potential application of artificial intelligence (AI) methods for developing advanced tests of candidate theories, and (5) we discuss what is learned from such applications and argue for a multi-pronged approach that can enable the scientific community to develop a working theory for albedo.

### Phenomenology and scaling challenges

**Fig. 1:**
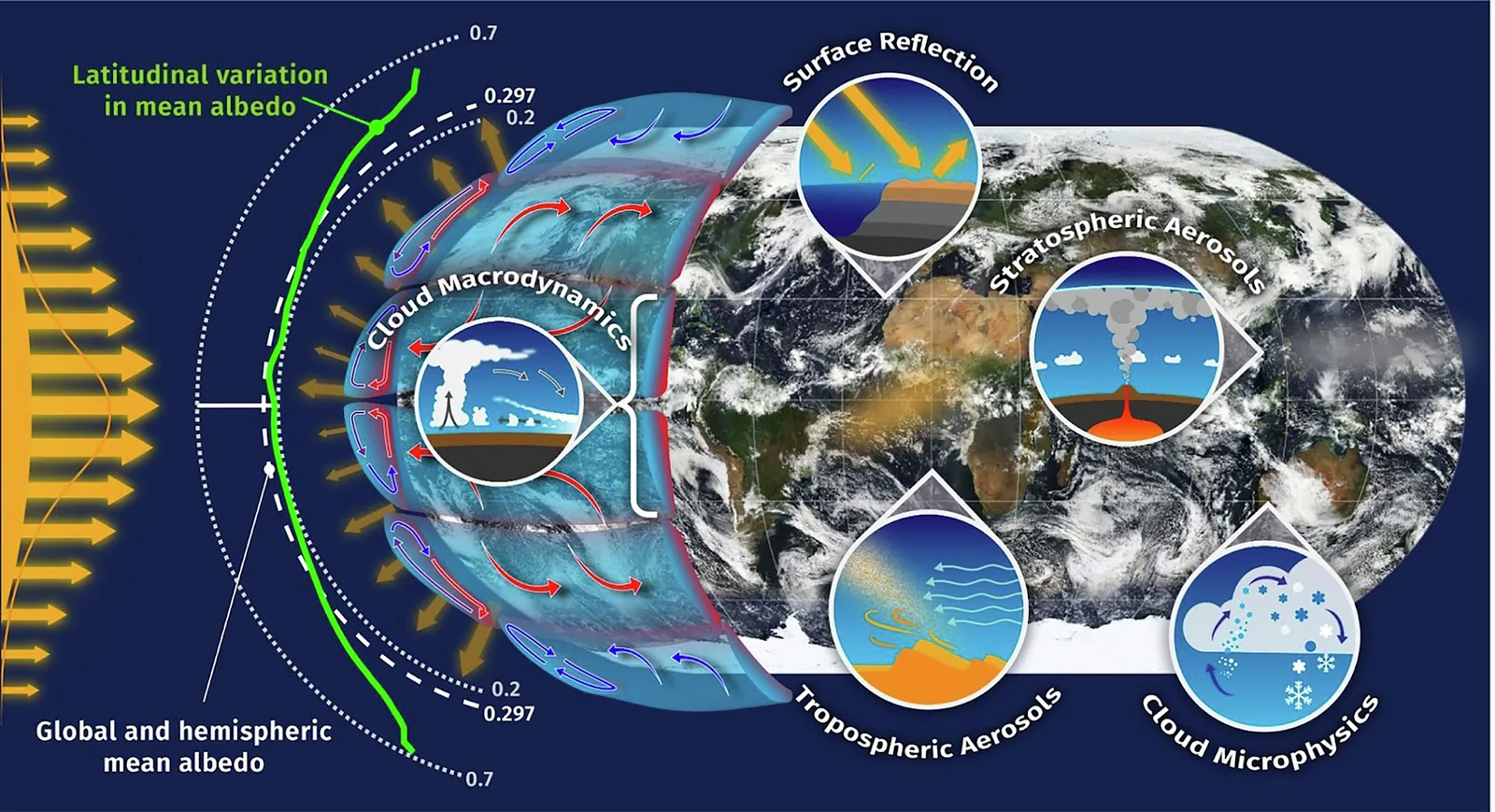
Infographic of the surface and atmospheric state features that interact to produce the top-of-atmosphere globally and zonally-averaged albedo, including the general circulation, surface reflection, cloud microphysics and macroscopic properties, and tropospheric and stratospheric aerosols. Gas absorption and molecular scattering are not depicted in this illustration. The satellite Earth image element of this figure is a composite of NASA MODIS Terra and Aqua true color corrected reflectance observations collected on July 11, 2005, and filled with the NASA Blue Marble Base Map for July south of 60°S.

The primary reason for the gap between the phenomenology and a predictive understanding of the albedo phenomena is that there is a tremendous range of spatial and temporal scales of processes that produce the observed phenomena. The top-of-atmosphere albedo is principally impacted by the global distribution of atmospheric condensates (aerosols, clouds, and combinations thereof) and surface reflection, which are affected by many dynamical, thermodynamical, and radiative processes, to name a few. The spatial and temporal scales at which clouds form and dissipate establish the radiative effects of clouds. But that belies the complexity of these effects, since cloud-controlling factors^30,31^ can also influence those effects. The range of scales is astounding: cloud microphysical processes, acting at micrometer spatial scales and sub-hour temporal scales, impact the formation and dissipation of individual clouds^32^. At the same time, satellite observations clearly show that cloud systems are organized on continental and larger spatial scales (tens of thousands of kilometers)^33^. In terms of temporal scales, clouds impact albedo on time-scales of cloud formation and dissipation (sub-hourly)^34^ while cloud feedback research indicates changes in cloud reflectivity can occur at the time-scale of the entire 21st Century^30,35,36^. The known processes that jointly and separately impact albedo are depicted in Fig. 1.

Meanwhile, aerosols also have direct and indirect impacts on TOA albedo. Again, like with clouds, there are micrometer spatial and temporal scales at which aerosols form and evolve, but they also interact with clouds regularly, and, independently of aerosol–cloud interactions, aerosols can directly impact globally-averaged albedo on global and multi-year time-scales, such as through stratovolcanic eruptions^37–39^.

The processes that drive variability in surface reflection generally vary on longer spatial and temporal scales than those that impact clouds and aerosols. Surface reflection changes are governed primarily by frozen surface extent, which varies on synoptic time-scales and regional spatial scales, and vegetation, which varies on intra- and inter-annual time-scales and sub-regional spatial scales, and can be impacted directly by anthropogenic activities.

Nevertheless, the wide range of relevant temporal and spatial scales of Earth system features that impact albedo frustrates efforts to advance the predictive understanding of this quantity. To date, the contributions of these terms to albedo have been diagnosed reductively, rather than holistically: that is, the contributions of condensates and surface reflection to albedo can be quantified at each grid box and also in different regions (e.g., low-latitude high-clouds, and mid-latitude stratocumulus clouds) and summed up at hemispheric and planetary scales. This reductive diagnosis is helpful for characterizing past events, but the community is still seeking to understand if the observed phenomena are coincidental or if there are teleconnections that produce these phenomena that are the result of fundamental, and yet-to-be-determined, physical mechanisms.

There are three issues here. First, there is a lack of a theory to explain the observations, so that additional observations merely lengthen the time-series of recorded albedo without confirming or falsifying a predictive understanding of the phenomena. Second, the complexity of the process interactions may require different approaches and/or advanced data analytic methods that are outside of the traditional geoscience approaches. Third, it is unclear the extent to which Earth System Models can assist in advancing this predictive understanding.

Because of these issues, we are working here towards a theoretical framework supported by data analytics and modeling approaches that will help to predict how albedo will evolve in the 21st Century.

### Necessary conditions for a working albedo theory

We have identified four requisite features of a theory for globally-averaged and hemispheric albedo, as well as several non-requirements.

First, the working theory must produce observationally-testable predictions of the globally-averaged and hemispheric values of Earth’s albedo in the 21st Century (and not necessarily prior to, or subsequent to, this period). Specifically, the theory does have to predict globally-averaged and hemispheric albedo values on interannual time-scales and hemispheric spatial scales for a given level of anthropogenic radiative forcing, with specified greenhouse gas, land-use change, and aerosols. Second, any working theory must be consistent with the existing observational record, recognizing that record’s observational uncertainties (see refs. ^40,41^ for two such examples). Third, the working theory must be falsifiable. That falsification requirement may need to be satisfied with a longer observational record than exists at the time that a working theory is put forth, but the theory must be eventually falsifiable during the 21st Century, preferably as early as possible. Finally, the working theory must not require exhaustive albedo observations in the 21st Century to produce albedo predictions.

It is also important to point out features of a working theory that are not required: (1) the working theory has no requirement for elegance: there may be multiple processes that produce the observations, so the emergent phenomena may well not be amenable to short-form representations or descriptions; (2) the working theory does not have to produce analytic solutions from which the observed albedo phenomena emerge; (3) the working theory does not have to explain why the present-day value of Earth’s albedo is 29%; rather, it only is required to make testable predictions about whether changes in albedo stability or symmetry will occur and (4) the working theory does not have to explain globally-averaged albedo stability or symmetry for time periods outside of the 21st Century or even in more general cases for other planets. To be sure, it is preferable for a theory to encompass Earth System states outside of the 21st Century, but there are no observations and no known observational proxies of past (or future) top-of-atmosphere albedo outside of direct observations. The albedo of those Earth system states that differ radically from the last several decades can only be modeled, so the applicability of a theory across these other states can only be developed and tested by models.

We bring up these non-requirements to note to the community that future activities focused on the development and testing of working theories to understand and predict the Earth’s albedo should consider a very wide range of potential explanations, irrespective of their elegance, simplicity, ease of evaluation, universality, or whether the observed albedo necessarily emerges from the theory.

**Fig. 2:**
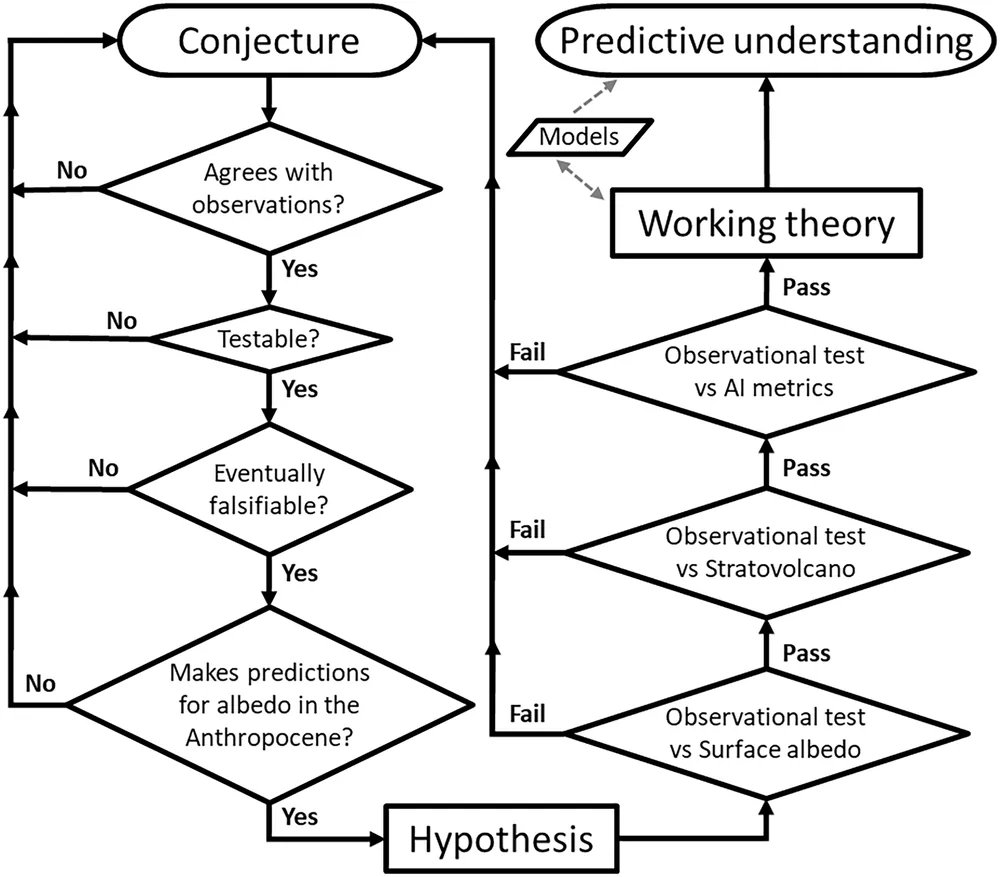
Flow-chart for the development of a working theory of globally-averaged albedo stability and hemispheric albedo symmetry.

Figure 2 depicts a flow-chart for the development of such a working theory. It highlights how there are numerous tests to subject each potential hypothesis, some of which can be performed with observations that exist, and some of which may require observations that do not yet exist. There are some elements of this flow-chart that follow traditional elements of scientific inquiry, but we note that there are key specifics that are worth highlighting. First, the role of stratovolcanic perturbations to albedo in theory development is paramount. Second, the scope of temporal and spatial scales spanned by a candidate theory is very large, so it must be tested across these scales with advanced analytics. Third, Fig. 2 recognizes that the observational data record for albedo spans several decades, and a viable theory may require more observations, so the establishment of testable observational corollaries is also paramount.

## Results

### Range of climate models projections

**Fig. 3:**
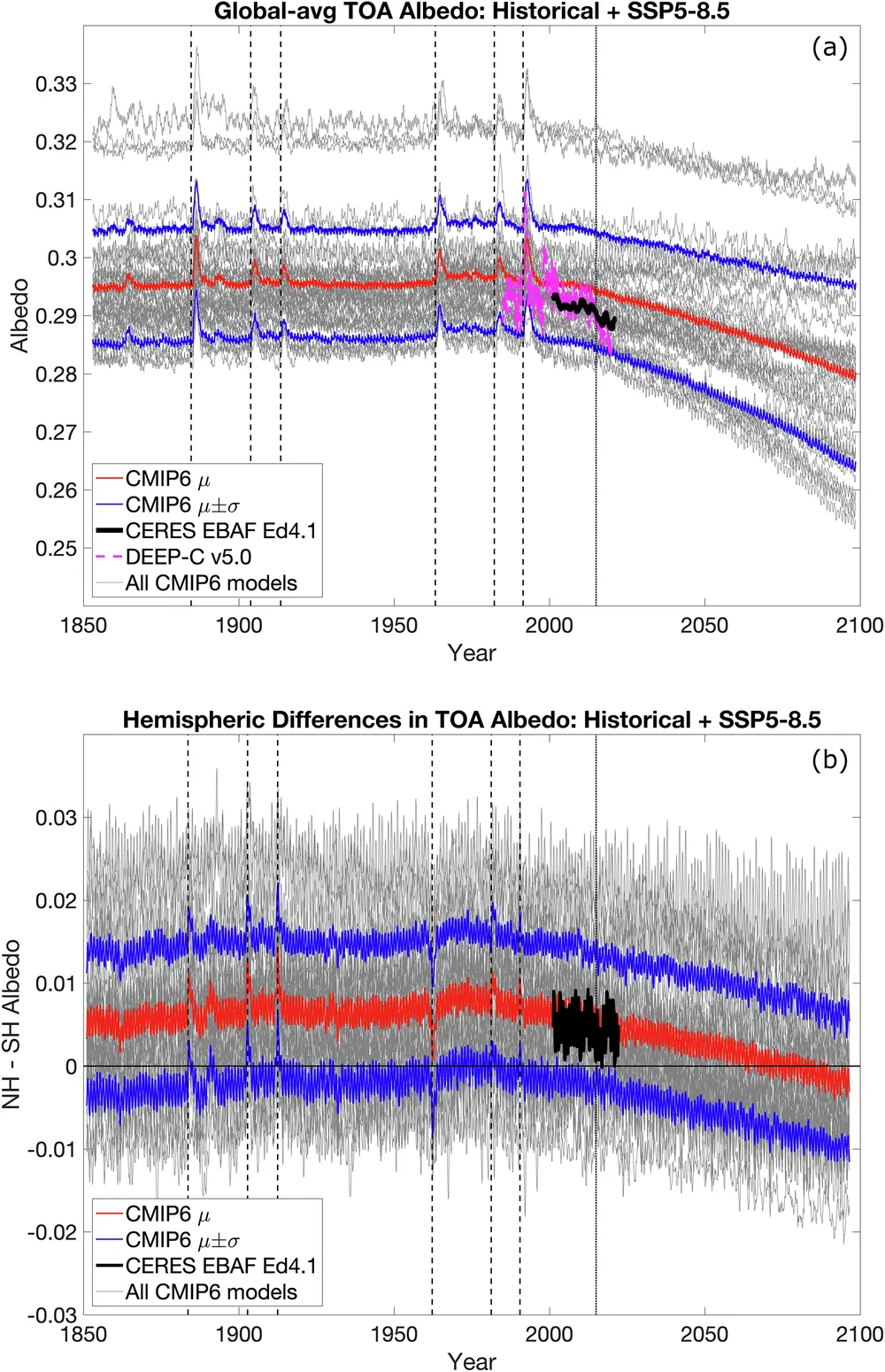
Globally-averaged TOA albedo as measured by CERES-EBAF (black), an albedo reconstruction from DEEP-C data and the Kopp solar database (pink dashed), and historical simulations and future projections from a suite of CMIP6 models with the multi-model average and standard deviation depicted. Vertical dashed black lines indicate VEI-5 or greater stratovolcanic eruptions. Vertical dotted line marks the end of CMIP6 historical simulations (**b**). Same as **a** but for Northern hemisphere average albedo minus Southern hemisphere average albedo and without the planetery-averaged DEEP-C albedo historical reconstruction.

Climate models exhibit a range in their TOA globally-averaged albedo fields over the historical period (prior to 2015), but the mean follows observations. The multi-model ensemble mean also roughly follows the observed albedo from CERES and also roughly follows the albedo field that was reconstructed prior to the start of the CERES record^10^ (see Supplemental). However, these climate models exhibit a large range in their projections of globally-averaged and hemispheric albedo change in the 21st Century that is well beyond observational uncertainties^42^. This is shown in Fig. 3, which analyzes the output from 40 different models from the Coupled Model Intercomparison Project—Phase 6^43^ both for their historical simulations and their simulations of 21st*-c*entury albedo with the Shared Socioeconomic Pathway 5 with 8.5 W/m^2^ radiative forcing relative to pre-industrial conditions (SSP5-8.5)^44^. The range of albedo projections for the 21st Century corresponds to over 2.5 W/m^2^ of shortwave radiation that is available, or not, to the Earth’s climate system at the end of the 21st Century, depending on which climate model’s projections are used.

As the advancement of climate models has pushed climate models to greater complexity, the biases that the climate models have exhibited in their simulated top-of-atmosphere albedo relative to CERES observations have persisted^45^, as have the magnitude of hemispheric albedo differences^18^. The reasons for this persistence are still being investigated. For global averages, there may be issues with model representations of aerosols^46^ as well as challenges they face with the apparently changing role of clouds in contributing to albedo^47,48^. For hemispheric symmetry, several reasons have been identified, including (1) how realistic the models are in terms of reflected shortwave radiation in midlatitudes, and (2) the representation of cloud responses to coupled ocean-atmosphere processes^20^.

At the same time, the skill of the multi-model ensemble mean must be recognized with respect to its reproduction of observations, both over the historical period (prior to 2015) and since 2015 when the models were forced by scenario projections. While some of this skill can be attributable to energy imbalance constraints in climate model tuning^26–29^, this skill indicates that experiments with an ensemble of different models may support theory development, even if individual models may not. Figure 3 shows how closely this mean tracks the CERES-observed albedo over time, both for global averages and hemispheric differences. The models notably track recent observed declines in albedo that appear to be, in part, due to aerosols^46^. Figure 3 also shows that the response of models to the Mt. Pinatubo stratovolcanic eruption was at least moderately realistic, even if the magnitude of the modeled perturbation was lower than observed and the relaxation times of that perturbation were longer than observed. These findings suggest that the Central Limit Theorem may be applicable for analyzing multiple models, performing experiments with the ensemble, and gaining insights into the observed albedo phenomena because it may be appropriate to assume that each model is an independent, if imperfect, sample of possible globally- or hemispherically-averaged albedo.

Ultimately, the physical mechanisms and their connections that lead to the observed phenomena of albedo stability and symmetry remain subjects of active scientific investigation. There is a role for a working theory to describe the emergent properties of top-of-atmosphere albedo in observations and climate models so that that theory can be explicitly represented in such models.

#### Invariant Earth system properties hypothesis

To illustrate a path forward towards theory development, we present two hypotheses and their observational corollaries, though note that these are merely examples and there may be other, superior candidate hypotheses. The first hypothesis is that the Earth’s top-of-troposphere globally-averaged albedo and its hemispheric symmetry are the result of properties of the Earth system at the surface and in the troposphere that are invariant on the time-scales of the 21st Century.

More specifically, the small range of albedo on interannual time-scales may be constrained by a set of physical constants far more than the range of possible spatial and temporal variations of the Earth’s atmospheric and surface states.

Invariant properties of the Earth system at the surface and in the troposphere that materially impact albedo include, but are not limited to, the following:

- Earth’s orbital parameters and rotation rate, which impact time-varying insolation levels.
- The fraction of Earth covered by land and its geographic configuration affect surface reflection.
- The refractive indices of liquid/ice water, which impact cloud reflection and frozen/non-frozen surface reflection.
- Water vapor spectroscopic absorption, which affects atmospheric shortwave radiation.
- Water thermodynamic phase transitions, which impact the formation of clouds, snow, and ice.
- Atmospheric composition of gases that absorb shortwave radiation.

At the same time, we recognize that recent observations of albedo suggest that globally-averaged albedo may not be completely invariant on interannual time-scales^22^, so modifications to this hypothesis may be required, perhaps to account for the impact of changing atmospheric composition on the turbulence that sustains clouds driven by longwave radiative cooling of the cloud tops^49,50^. Additionally, we recognize that stratospheric reflectivity can vary independently of the troposphere and materially impacts TOA albedo (e.g., with stratovolcanic eruptions), so this hypothesis is focused on the top-of-troposphere reflectance only.

If globally- or hemispherically-averaged albedo is indeed constrained by Earth System constants, there are observational corollaries to this hypothesis that Earth Radiation Budget (ERB) measurements like CERES and Libera can test. First, the top-of-atmosphere albedo should be invariant to significant changes in surface albedo. Indeed, globally-averaged albedo has not responded in any significant way to transient changes in surface albedo that have regularly occurred due to changing frozen surface extent ^51,52^. Second, globally-averaged and hemispheric albedo should also be invariant to significant changes in tropospheric aerosols. The reason for this is that there have been many short-term fluctuations and long-term trends in anthropogenic aerosols^53^, especially sulfates, and there is a significant asymmetry in hemispheric aerosol loading, yet aerosols have not definitively impacted the TOA albedo stability or symmetry. Third, the relaxation of the Earth’s albedo following two or more similar stratovolcanic perturbations must be similar. Finally, following the previous corollary, the increase in TOA albedo must increase monotonically with the light scattering of the material injected into the stratosphere.

#### Cloud buffering hypothesis

The second hypothesis that we present is that the Earth’s globally-averaged albedo maintains its stability and symmetry through cloud buffering^3^. According to this hypothesis, the albedo of Earth appears to be highly constrained because clouds’ scattering compensates for variations in surface albedo and aerosols that would otherwise impact albedo. This hypothesis also has observational corollaries that can be tested with ERB measurements. First, globally- or hemispherically-averaged cloud radiative effects must respond inversely to large changes in surface albedo. Second, a major stratovolcanic event like Mt. Pinatubo or other perturbations to globally-averaged albedo would, according to this hypothesis, lead to a global, detectable reduction in cloud radiative effects to compensate for the rise in aerosol scattering. Any compensating cloud radiative effects would also need to account for the fact that stratospheric aerosols may also immediately brighten clouds via diffusion of the radiation field^54^. The hypothesis would also suggest that, after a stratovolcanic eruption in one hemisphere, the cloud radiative effects would respond differently in each hemisphere, and the albedo of the other hemisphere would also increase.

Furthermore, there are AOD limits for this hypothesis that can be explored, while still noting that a working theory does not have to encompass these limits. In the limit of very large AOD on Earth (e.g., the Chicxulub asteroid^55,56^, which likely produced globally-averaged aerosol optical depths of greater than four^57–59^), there should be much less cloud radiative effect. Conversely, in the limit of no aerosols, the cloud-buffering hypothesis predicts that there should be a higher cloud radiative effect than at present, even though cloud formation will be slower^60^ in that limit.

#### AI methods to test albedo hypotheses

Given the scaling challenge associated with albedo analysis, we note that Artificial Intelligence (AI) methods, when properly implemented, may be particularly relevant since they are well-suited to discerning connections, both obvious and subtle, across temporal and spatial scales in Earth science^61^. The scientific community studying albedo may be able to gain additional understanding about the stability and symmetry phenomena with methods such as these, especially because they can discern subtle connections across temporal and spatial scales that may be contributing to these phenomena.

Here, we develop AI data models to illustrate their utility in assisting theory development. To be clear, these methods by no means supplant the scientific approaches previously discussed here, but can provide guidance on research directions. We use these AI models to analyze the importance of, and teleconnections between the TOA albedo field and cloud radiative effects, AOD, and surface albedo using observations and ancillary data from 20 years of CERES EBAF product data^62^.

We first develop a feed-forward neural network (FFNN) in order to determine the relative contributions of the atmospheric and surface states to the observed albedo (see Methods for details). This can be accomplished, in principle, with sufficiently accurate radiative transfer (RT) algorithms, but the reason for using a FFNN is that when it is properly configured, it can emulate high-complexity nonlinear functions (i.e., the radiative transfer) and we do not need to make myriad assumptions about RT model configuration (e.g., surface reflectance function, number of streams, whether or not to include 3-D effects, choices about condensate optical properties, etc. that can vary in space and time). Rather, the FFNN is able to incorporate realistic choices for those assumptions by learning from a large number of predictors and predictands to develop functional RT relationships to be consistent with the observations. Most importantly, FFNNs are easily incorporated into analyses of feature importance to understand which geophysical variables matter the most for albedo. This feature’s importance information can then be used to support a mechanistic understanding of how the albedo phenomena arise.

The feature importance analysis is performed with Shapley Additive Parameter (SHAP) analysis (See Methods for details), which produces a ranked set of contributions of each input atmospheric or surface state feature to TOA albedo values across space and time^63–66^. SHAP works by estimating each physical variable’s marginal contribution to the model’s predictions, regardless of the linearity of the model. SHAP therefore produces an interpretable measure of the impact of a set of atmospheric and surface state characteristics on albedo. This approach answers the fundamental question of why a model produces an output given a set of inputs when the inputs contribute differentially to the outcome. Because SHAP is a model-agnostic framework, it allows us to focus first on achieving sufficient predictive skill with the FFNN and then apply SHAP as an interpretability layer to diagnose which physical parameters the model relies on most strongly in producing those predictions.

**Fig. 4:**
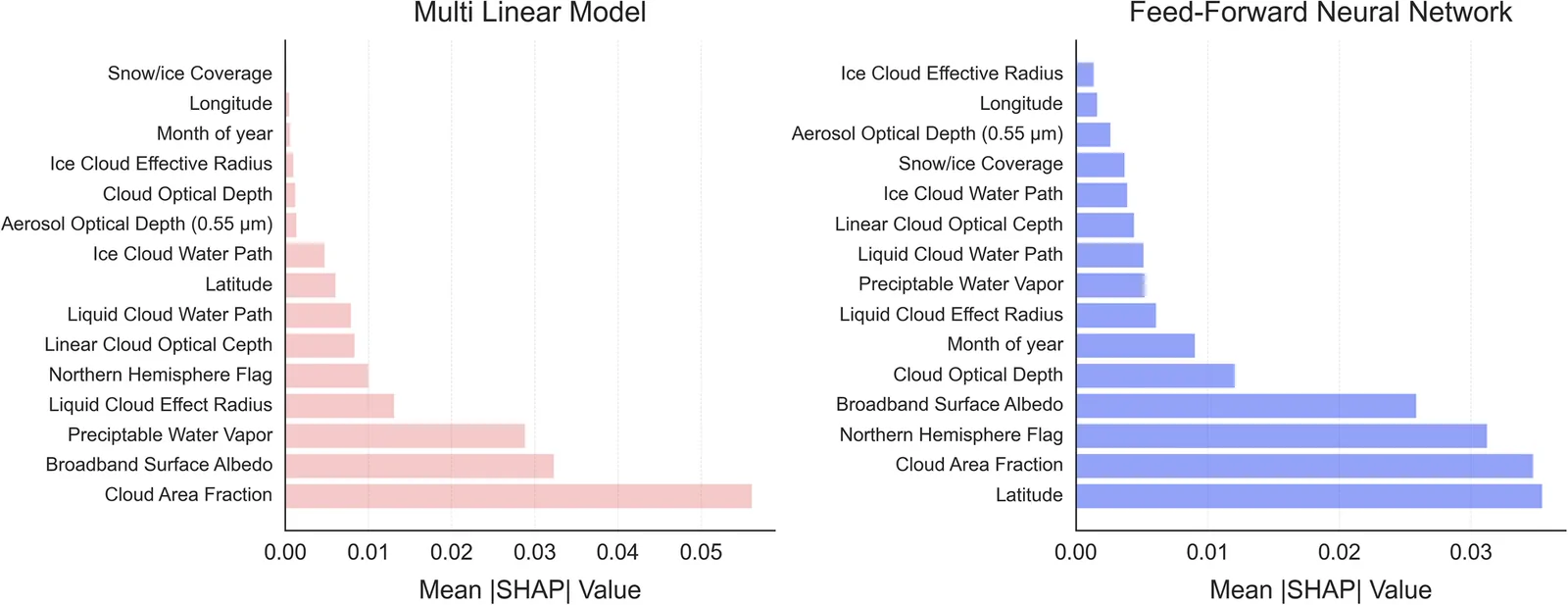
Mean SHAP values showing the relative importance of features for explaining the 2000–2023 CERES-EBAF Ed4.1 albedo data record using a multi-linear regression (MLR) and a nonlinear feed-forward neural network (FFNN), where spatial patterns can be learned. There is a different ranking of feature importance values for the MLR and FFNN that suggests different research directions from the linear method and the FFNN.

The SHAP analysis of the FFNN is performed across all CERES EBAF grid cells and is shown in Fig. 4. We also present a SHAP analysis of a multi-linear regression (MLR) model to compare results from two data models of very different complexities (see Supplemental for details). Both methods indicate that cloud area fraction is, unsurprisingly, important for the prediction of globally-averaged and hemispheric albedo. More surprisingly, though, is that the SHAP analysis of the FFNN also shows the importance of latitude but the lack of importance of longitude and time for controlling a given location’s albedo. This finding suggests that the processes that are responsible for albedo at each and every grid box have been stationary over time, and also that they vary far more meridionally than zonally. This finding further suggests that the development of a working theory is well-posed, given the zonal and temporal stationarity of processes that produce the observed albedo phenomena. The different order of importance variables in the MLR model and the superior performance of the FFNN model (see SI Appendix) both highlight the need for data models that can represent non-linear interactions and indicate that insights into albedo phenomena require data model complexity and, conversely, albedo research can be misled by simple data models (see Supplemental for details).

While the FFNN and SHAP provide insight into how surface and atmospheric state characteristics produce the albedo phenomena for each grid box, they do not explicitly capture teleconnections. They treat each grid box largely independently and do not explicitly model spatial or temporal structures across multiple scales. Since albedo stability and symmetry phenomena may depend on large- or even medium- or small-scale teleconnections, we require an approach capable of representing more complex spatio-temporal patterns.

In physical sciences in general and climate science in particular, there is precedent for using U-Net architectures (see “Methods”)^67^ for these types of geophysical correlation analyses^68,69^. The U-net modeling approach analyzes datasets like albedo and its underlying atmospheric and surface states that vary in space and time for those essential features that can be used to reproduce the albedo dataset being analyzed. This approach is analogous to approaches used to compress and expand images while retaining the underlying information in the images. Ultimately, the U-net can then be used to predict how the albedo will vary in space and time for different combinations of atmospheric and surface states.

The FFNN & U-Net are modeling different levels of complexity. The FFNN is essentially learning radiative transfer and basic spatial patterns to establish the interactions between different processes that impact TOA albedo for a given grid box. At the same time, the FFNN does not have to learn as much about the complexities of spatial and time scales as a U-Net. The U-Net is performing a set of operations to compare different levels of model complexity in order to determine the optimal level of complexity for albedo prediction, since it is not known a priori.

**Fig. 5:**
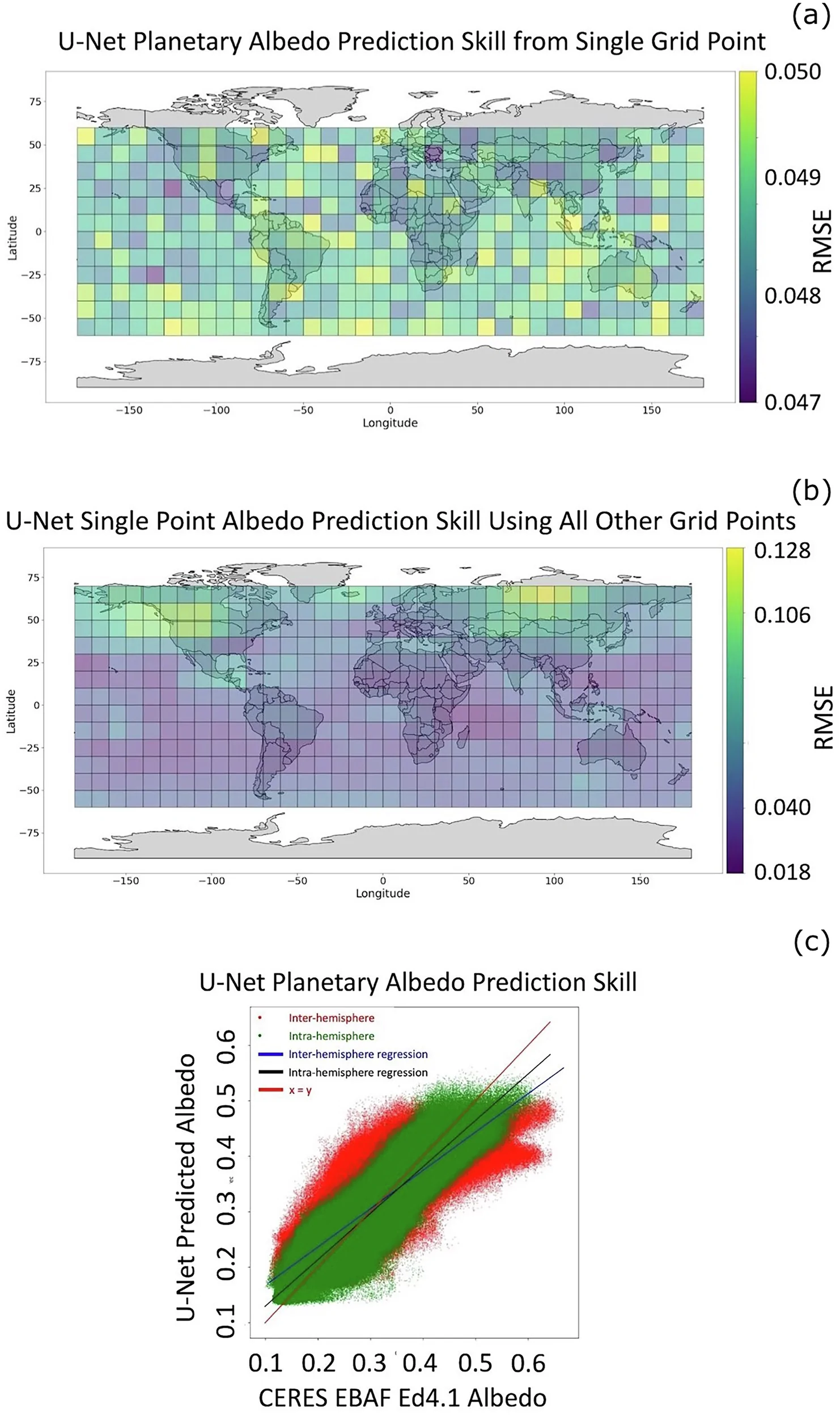
**a** RMSE of the skill of a U-Net to predict, from atmospheric/surface state and observed albedo in a single grid cell at a single time-step, the globally-averaged albedo at that time-step. **b** RMSE of the skill of a U-Net to predict, from the atmospheric/surface state and observed albedo at a single time-step, with all grid cells except the one being predicted, and the albedo at that single grid cell. **c** Predicted vs. observed albedo of the U-Net shown in (**a**) within and across hemispheres.

In order to probe the spatial and temporal correlation structures that contribute to albedo phenomena, we use a U-Net to determine if, where, and/or when there are robust teleconnections that underlie the albedo phenomenology. We first explore whether there are regions that control globally- or hemispherically-averaged albedo. We do this by training a U-Net just on a subset of EBAF predictor data from a single grid box and a corresponding subset of the spatially- and temporally-resolved EBAF albedo field as the predictand. We then use that model to predict the EBAF albedo at all grid boxes and determine that model’s error. We repeat this training, prediction, and error determination for all grid boxes, and plot the results in Fig. 5a. The lack of a discernible structure in this plot is apparent. Accordingly, this plot shows that there is no particular region or set of regions on Earth that exerts strong control on the spatial and temporal evolution of globally-averaged albedo, because if there were, the U-Net would learn robust relationships between that grid box and global albedo predictands and would then exhibit a lower RMSE than the other regions. What we actually see is a small range of RMSE values from each grid box.

Meanwhile, Fig. 5b represents a complementary analysis, but one focused on whether a given region’s albedo is largely determined by the albedo, atmospheric, and/or surface states in other regions. Figure 5b evaluates this approach and shows that the prediction skill of albedo in one location using the rest of the current and historical atmospheric and surface information is similar across the Earth, with the exception of some areas of North America and Siberia. These results generally show regional agnosticism with respect to albedo prediction.

Finally, we use a U-Net trained on predictors and predictands from one hemisphere and determine if it can predict albedo in that same hemisphere or the other hemisphere. Figure 5c shows that predicting albedo in the other hemisphere is a more difficult task than predicting albedo in the same hemisphere. Those findings suggest that there are fundamental differences between processes that control albedo in one hemisphere and those that control it across the globe.

In light of these findings, we circle back to Fig. 5a and note that the spatiotemporal correlation between albedo in one region and in others is not a function of clear or cloudy regions. Consequently, the buffering of albedo by clouds is either not occurring, or at least not seen in efforts to discern whether some regions contribute more than others to the globally-averaged albedo. We expected that regions that have higher surface albedo and/or larger cloud amounts and/or brighter or more variable clouds would serve as better predictors for globally-averaged albedo, but we do not see this. Rather, we see that each location provides approximately equal predictive power as others, which is more consistent with an explanation that albedo arises from properties shared by each grid box, rather than ones that are unique to a given set of grid boxes.

Finally, we can use the trained U-net to determine if, as the cloud buffering hypothesis suggests, there are indeed inverse relationships between cloud fraction and surface albedo at scales that are significant for regulating the Earth’s globally- or hemispherically-averaged albedo. As shown in the Supplemental Fig. 2 in the SI Appendix, we find that these inverse relationships do indeed exist, although they are quite weak and suggest that large changes in surface albedo may not be offset by equivalently large changes in cloud radiative effects.

The insights provided by this initial study encourage the further application of deep learning models for scientific discovery related to Earth’s albedo through perturbative analysis.

#### Insights from historical and future stratovolcanic eruptions

Stratovolcanic eruptions with ejecta of more than 1 km^3^ (Volcanic Explosivity Index (VEI) of 5 or more) temporarily increase globally-averaged albedo, and possibly change hemispheric asymmetries. After a few years, that perturbation to albedo relaxes. Figure 3 shows the modeled impact of 6 stratovolcanic eruptions on globally-averaged albedo: Krakatoa, Indonesia, in August 1883; Santa María, Guatemala, in October 1902; Novarupta, Alaska, in June 1912; Mount Agung, Indonesia, in March 1963; El Chichón, México, in March 1982; and Mt. Pinatubo, Philippines, in June 1991.

These events are significant for globally-averaged albedo: the Mt. Pinatubo eruption of 1991 increased globally-averaged albedo by 0.007 (2.4% relative increase), produced direct and indirect changes in albedo over clear and cloudy areas, and diminished within a few years as those aerosols settled out of the stratosphere^37–39^.

Stratovolcanic eruptions may provide a large amount of information on those underlying principles that control albedo, as the perturbative increase of albedo in space and time and the subsequent relaxation of that perturbation can reveal the underlying mechanisms governing albedo stability and symmetry. There are at least four reasons that stratovolcanic eruptions provide insights into the mechanisms that control albedo:

1. They produce large perturbations in globally-averaged albedo that are directly attributable to a single event, where the amount of aerosols injected into the stratosphere can be independently estimated. Consequently, the central estimate of, and the upper and lower bounds on, the direct impact on albedo from those aerosols can be estimated from reconstructions of total ejecta, composition, aerosol size distribution, and optical properties and evaluated against other Earth system changes that also influence albedo.
2. Stratovolcanic aerosols and their influence on albedo decay over time due to settling, allowing for observations of that decay and of Earth’s albedo response to that decay.
3. Stratovolcanic eruptions occur in one hemisphere only and typically mix on seasonal timescales in that hemisphere, while mixing in the other hemisphere occurs on semi-annual to annual time-scales^70^. Therefore, the response of the other hemisphere can reveal cross-hemisphere teleconnections that contribute to hemispheric albedo symmetry.
4. The challenge of disentangling aerosol-cloud physical and chemical interactions from the radiative influences of aerosols and clouds on albedos is largely avoided: Consequently, stratovolcanic-generated aerosols produce radiative effects that influence albedo in the stratosphere, while clouds produce radiative effects that influence albedo in the troposphere.

A comparison of the reconstruction of the globally-averaged albedo response to the Mt. Pinatubo eruption in models and observations, as shown in Fig. 3 and in Supplementary Fig. 3, suggests that the Earth’s actual response to this forcing was larger, had a faster onset, and also a faster decay than the multi-model ensemble and individual models. When future stratovolcanic eruptions occur, satellites will be able to measure the albedo field in clear and cloudy scenes, teleconnections in albedo, and albedo change over time in response to a stratovolcanic eruption, along with dozens of variables in the atmosphere and at the surface that are known to directly or indirectly impact albedo. They will also be critical for the testing of a working theory.

Specifically, for the two hypotheses presented here, a decrease in cloud radiative effects from a VEI-5 or greater stratovolcanic eruption would support a cloud-buffering hypothesis, but if cloud radiative effects remain unchanged, then cloud-buffering is rejected, and the invariant Earth System properties hypothesis would be more supported.

## Discussion

The challenges of understanding and predicting Earth’s albedo in the 21st Century are formidable. In spite of decades of observational datasets and dozens of climate models’ projections of albedo, a working theory for albedo phenomena has eluded the scientific community. In order to make substantive progress towards a working theory, the challenges of spatial and temporal scales that underlie the observed phenomena must be addressed head-on by the community. Fortunately, hypothesis-driven investigations that use several underutilized or unutilized tools can provide additional insights into the nature of these phenomena. As such, we have shown that there are three approaches that can help evaluate these hypotheses beyond what has already been discussed in previous publications: (1) advanced data methods, (2) stratovolcanic eruptions, and (3) multi-model ensemble experiments.

Future stratovolcanic eruptions will likely falsify at least one of the hypotheses presented here, while advanced data methods, including the use of AI, indicate weak support for the cloud buffering hypothesis. Finally, climate models, when they structurally support a working theory for albedo, may be useful in predicting albedo and probing the limits of albedo stability and symmetry. The skill of the multi-model ensemble in reproducing observed albedo phenomena and emerging (though not definitive) trends suggests that variational analysis (e.g., in ref. ^23^) and experiments with the ensemble are warranted and may be supportive of theory development.

There are several important caveats to what we presented. First, we must be careful to note that globally-averaged albedo stability and hemispheric albedo symmetry may be independent phenomena. We simply present them both here because the approaches for advancing towards a working theory for either of these phenomena are similar.

Second, we present two hypotheses and their observational corollaries merely as examples to the community. Alternative hypotheses that differ from what was presented here, but at the same time meet the requirements for advancing to working theory set forth here, are needed.

Third, there is a need to evaluate the data analysis techniques, whether they are low-complexity, high-complexity, or somewhere in the middle. We present a moderately high complexity set of techniques with an FFNN and a U-Net. We recognize that our approach may not be achieving an optimal level of complexity and skill for advancing scientific studies of albedo, but we need to achieve a balance between complexity (to handle nonlinearities) and model predictive skill so that such a model can provide leading (and not misleading) guidance to theory development. Our methods show that FFNN methods achieve a balance of skill in predicting out-of-sample data and model complexity in terms of metrics that balance these competing features. While the FFNN may not be the most optimal model, it is more optimal than linear models (see “Data Model Complexity” in Supplemental for details).

Still, the need for a working albedo theory remains. With longer time series of albedo measurements and potentially greater perturbations or secular changes in spatial patterns of albedo and hemispheric and global averages, one or more candidate theories may be able to emerge as viable. As the 21st Century proceeds, the growing data record of observations from CERES, and the continuity of Earth Radiation Budget observations that Libera will produce^71,72^, may show unprecedented changes in the Earth’s albedo field. The Earth Radiation Budget community must be ready to explain such changes, should they occur. Currently, such an explanation would be diagnostic: the community will be able to explain where on Earth the albedo has changed and the local atmospheric and surface conditions that will have contributed to such changes. However, the community will definitely be asked to predict future changes in globally- and hemispherically-averaged albedo, especially if albedo exhibits significant changes that might indicate a climate tipping point^73^. A community-level theory development effort will be needed at some point in the 21st Century, and the approaches discussed here, along with our preliminary findings, hopefully will help support that effort.

## Methods

### Climate model data

The data that produced Fig. 3 are from two sources. Each of the 40 climate model time-series displayed was downloaded from the Climate Data Store (https://doi.org/10.24381/cds.c866074c) and produced by dividing the top-of-atmosphere outgoing shortwave radiation (output variable name: “rsut”) by the incident shortwave at the top of the atmosphere (output variable name: “rsdt”). We chose the following ensemble members for the historical DECK experiments (1850-2014), concatenated with the SSP5-8.5 experiment (2015-2100). These were chosen because they represented each of the modeling center submissions to CMIP6 that reported the necessary fields to show historical and 21st-century projected globally-averaged and hemispheric albedo. • ACCESS-CM2, r1i1p1f1 • ACCESS-ESM1-5, r1i1p1f1 • AWI-CM-1-1-MR, r1i1p1f1 • BCC-CSM2-MR, r1i1p1f1 • CAMS-CSM1-0, r1i1p1f1 • CCCR-IITM, r1i1p1f1 • CCMA, r1i1p1f1 • CESM2-WACCM, r1i1p1f1 • CESM2, r1i1p1f1 • CMCC-CM2-SR5, r1i1p1f1 • CMCC-ESM2, r1i1p1f1 • CNRM-CM6-1-HR, r1i1p1f2 • CNRM-CM6-1, r1i1p1f2 • CNRM-ESM2-1, r1i1p1f2 • CanESM5-CanOE, r1i1p1f2 • E3SM-1-1, r1i1p1f1 • EC-Earth3-CC, r1i1p1f1 • EC-Earth3-Veg-LR, r1i1p1f1 • FGOALS-f3-L, r1i1p1f1 • FGOALS-g3, r1i1p1f1 • FIO-ESM2-0, r1i1p1f1 • GFDL-CM4, r1i1p1f1 • GFDL-ESM4, r1i1p1f1 • GISS-E2-1-G, r1i1p1f1 • HadGEM3-GC31-LL, r1i1p1f3 • HadGEM3-GC31-MM, r1i1p1f3 • IITM-ESM, r1i1p1f1 • INM-CM4-8, r1i1p1f1 • INM-CM5-0, r1i1p1f1 • IPSL-CM6A-LR, r1i1p1f1 • KACE-1-0-G, • KIOST-ESM, r1i1p1f1 • MIROC-ES2L, r1i1p1f2 • MIROC6, r1i1p1f1 • MPI-ESM1-2-LR, r1i1p1f1 • MRI-ESM2-0, r1i1p1f1 • NESM3, r1i1p1f1 • NorESM2-MM, r1i1p1f1 • TAIESM1, r1i1p1f1 • UK-ESM1-0-LL: r1i1p1f2.

### CERES EBAF data

We used the energy-balanced and filled (EBAF) version 4.1 of the Clouds and the Earth’s Radiant Energy System (CERES) mission (obtained from https://ceres.larc.nasa.gov/data/), which includes all-sky shortwave flux and incoming solar flux.

### Neural network architecture

The model used to produce Fig. 4 is a feed-forward neural network (FFNN)^74^. It can be downloaded here: https://github.com/samuelhkahn/ai-albedo-research. This particular FFNN consists of an input layer, 2 hidden layers, and an output layer which predicts top-of-atmosphere, spatially-resolved albedo.

The input layer size is determined by the dimensionality of the input data. In our case, it maps atmospheric and surface state variables from the CERES-EBAF Ed4.1 product, which include the following:

- Cloud Properties (MODIS and GEO) [Diurnally Complete]
  - Cloud Area Fraction
  - Cloud Effective Pressure
  - Cloud Effective Temperature
  - Cloud Effective Height
  - Cloud Top Pressure
  - Cloud Base Pressure
  - Cloud Particle Phase
  - Liquid Water Path
  - Ice Water Path
  - Water Particle Radius
  - Ice Particle Effective Diameter
  - Cloud Visible Optical Depth
- Aerosols (MODIS & MATCH)
  - MATCH Total Aerosol Optical Depth @ 0.55 μm
  - MODIS Aerosol Optical Depths over Land & Ocean
- Auxiliary Data (GMAO GEOS)
  - Surface Type Percent Coverage
  - Skin Temperature
  - Total Column Precipitable Water
  - Upper-Tropospheric Precipitable Water
  - Column Ozone
  - Longitude
  - Latitude
  - Timestamp

Each hidden layer comprises four pieces;

1. Fully connected layer: This layer can be thought of as a linear transformation. When data is passed through the fully connected layer, each input neuron is connected to all other neurons in the following layer. The dimensionality of outputs is called the hidden layer size or dimension.
2. Batch Normalization^75^: This is a technique for improving the speed, performance, and stability when training neural networks. It standardizes the inputs to a layer for each mini-batch, which stabilizes the learning process. This reduces how long we need to train the neural network to reach convergence.
3. The Rectified Linear Unit (ReLU)^76^: The ReLU is a non-linear activation function that is applied to the data as it flows through the network. The ReLU maps to zero when the input neuron is zero or less and has a linear mapping above zero. Non-linear activation functions such as the ReLU play a critical role for all neural networks to model complex non-linear phenomena such as the response of globally-averaged albedo to different perturbations of covariates. Without the ReLU, a neural network would behave just like a single-layer neural network since summing any number of linear transformations (fully connected layers) results in a linear function, which would restrict the type of phenomena we could model with the network.
4. Dropout^77^: This is a technique used to regularize a neural network to help improve generalization performance. While a network is training, a fraction of the total neurons are randomly ignored (dropped out) while data propagates through the network. The purpose of this is to temporarily remove the contribution of a dropped-out neuron during the forward propagation step of training, and weight updates are not applied to a dropped-out neuron during the backpropagation pass. This helps the model not rely too heavily on the activation of certain features or hidden neurons. Dropout is not applied at inference time.

The final layer of the network is a final fully-connected layer that projects the size of the last hidden dimension to the desired output. In the case of this paper, the dimension of the output is one because we are predicting a single value for the globally-averaged albedo.

This FFNN learned from the input data by adjusting the network’s weights through the backpropagation of error with gradient descent^78^. We use multiple hidden layers to allow the feed-forward neural network to learn complex representations of the input data. The learning of rich feature representations is a unique and effective feature of neural networks compared to more traditional statistical or machine learning models.

Normalization is a crucial step in preparing data for neural networks due to several reasons^79^. Transforming features onto a normalized scale prevents gradients from becoming too small or too large (vanishing and exploding gradients) during back propagation. It has been shown that normalization helps optimization algorithms converge more efficiently than without normalization [ibid].

We use the normalization technique, min–max normalization, which transforms numeric data to the range [0,1]. For a data point *x*, the min–max normalized value $x{\prime}$ is computed as:

1 $$x{\prime} =\frac{x-min (X)}{(max (X)-\min (X))}$$

where *X* is the set of all values for a feature, and min(X) and max(X) represent the minimum and maximum value of the input feature *X*, respectively. It must be noted that all features are min-max normalized independently.

We use the optimizer called Adam^80^. The Adam optimizer is a common stochastic gradient descent method used pervasively in deep learning. Adam is known for its computational efficiency and robustness when training. This Adam algorithm utilizes the first and second moments of the gradient to dynamically adjust the learning rate for each weight in the model.

These hidden layers are between the input and output layers and perform transformations on the input data. The first hidden layer directly interacts with the input layer. The activation function then introduces non-linearity, enabling the network to learn complex patterns. As we move deeper into the network, the hidden layers operate over the output projections of the previous hidden layer. These successive transformations, projections, and application of non-linearities in the hidden layers make deeper layers learn increasingly abstract and complex representations of the data. These layers model interactions between the features learned in the previous hidden layer. The final hidden layer transforms the learned representations such that they can be used by the output layer to make the final output prediction.

In the context of predicting globally-averaged albedo, we can imagine the deeper we go into the network, the more it learns to model complex interactions between input features like surface albedo and AOD. These interactions become more complex to understand deeper in the network, but we can probe the behavior of the model using a perturbative approach.

The performance of the FFNN we trained was evaluated using the root mean square error (RMSE) metric. Our neural network achieved a low RMSE (see Supplementary Information), indicating that the model’s predictions are close to the actual observations. To ensure an unbiased estimate of the model’s generalization capability, we use a hold-out set to report the performance of the network. This data were not seen in the training process.

Because FFNN’s are notoriously difficult to perform inference, we use an additional inference algorithm called Shapley Additive Explanations (SHAP)^81^ after the FFNN is trained. SHAP allows us to understand why the FFNN is making the predictions it does.

### SHapley additive parameter

The SHapley additive parameter (SHAP) analysis attributes a data model prediction to the specific contributions of individual features, thereby addressing the often-asked question: “Why did the model predict this value?” For linear models, in which the prediction is expressed as a weighted sum of input features,

2 $$f(x)={\beta }_{0}+{\sum }_{j=1}^{M}{\beta }_{j}{x}_{j}$$

the SHAP values have an exact closed-form solution:

3 $${\phi }_{i}(x)={\beta }_{i}\left({x}_{i}-E\left[{X}_{i}\right]\right.$$

where *ϕ*_*i*_(*x*) denotes the contribution of feature *i*, *β*_*i*_ is the coefficient estimated during training, *x*_*i*_ is the feature value for the given instance, and *E*[*x*_*i*_] is its expectation over the training distribution. In this setting, contributions are independent and additive, and no feature interactions are represented.

For general models, such as neural networks or random forests, SHAP values are defined by the cooperative game-theoretic formulation:

4 $${\phi }_{i}={\sum}_{z{\prime} \subseteq x{\prime} }\frac{(M-| z{\prime} | -1)!| z{\prime} | !}{M!}\left[{f}_{x}(z{\prime} \cup \{i\})-{f}_{x}(z{\prime} )\right]$$

where $z{\prime}$ denotes subsets of input features excluding predictor variable *i*, *M* is the total number of predictor variables, and ${f}_{x}(z{\prime} )$ is the model’s prediction given the inputs $z{\prime}$. By systematically perturbing inputs and assessing the resulting prediction differences, SHAP provides interpretable feature importances from linear models to neural network models, providing insights into the factors driving TOA albedo variation.

### U-net architecture

We use a standard U-Net architecture scheme^67^ as a means to capture the salient spatial and temporal patterns in albedo and other surface and atmospheric state variables that are responsible for the observed albedo phenomena. We use the U-Net to predict top-of-atmosphere albedo at one location based on the albedo and surface and atmosphere state information at all other locations on Earth and vice versa. The results of this U-Net are shown in Fig. 5. It can be downloaded here: https://github.com/samuelhkahn/ai-albedo-research.

We scale and pad the input to the U-Net using reflection padding so the first convolutional (C) layer can be applied correctly. We apply C with 64, 128, 256, 512, 1024 input channels (kernels). We apply transposed convolutions (CT) with 1024, 512, 256, 128, and 64 input channels (kernels). We apply 2-d max pooling (MP) operations and a rectified linear unit (ReLU) after all C and CT layers within each expansion and contraction block. The fully-connected (FC) layer reprojects the output of the last CT layer. Arrows represent skip connections between the expansion and the contraction phase. We use drop-out layers with values of 0.1 between each contraction, expansion, and reprojection block. This architecture is depicted in Fig. S1.

The U-Net’s contracting path consists of a series of contraction blocks. Each contraction block is made up of a convolutional layer to encode spatial information into “depth” information, encoding rich & learned features of the data. Each convolutional layer is followed by a max-pooling operation to reduce the spatial dimensions and a Rectified Linear Unit (ReLU) non-linearity as the output. This path is designed to capture the hierarchical features of the input image channels with each successive layer encoding higher-level semantic information.

The U-Net’s expansion path is designed to project the rich feature space back to the input image’s spatial dimensions. This is done through a series of expansion blocks that increase the spatial resolution of the feature maps. The expansion blocks are made of a transposed convolution layer to project the rich feature maps to spatial information. Each transposed convolutional layer is followed by a max-pooling operation to reduce the spatial dimensions and a rectified linear unit (ReLU) non-linearity as the output. Each expansion block is followed by a concatenation operation with the corresponding feature map from the contracting path. This enables the model to leverage both high-level semantic information and low-level spatial details, making it highly expressive.

The U-Net’s expansion path ends with a final fully connected layer that maps the features to a single output channel of spatial dimensions 16 × 16. We then crop the image to 10 × 10 and calculate the loss, which in our case is the mean-squared error, between the predicted albedo and true albedo in the 10 × 10 patch. Drop-out is applied with values of 0.1 between each contraction, expansion, and reprojection block to help regularize the model.

Finally, we perform a series of ablation studies on this U-Net first to ensure that its performance degrades when it does not have access to the predictor variables it needs to predict albedo and then to identify which input variables produce the most degradation in model skill when they are removed from the U-Net, or replaced with a constant value. This is an important step to ensure that the model is robust to physically realistic perturbations, and, conversely, that the model performance degrades substantially when it is perturbed such that it is trying to predict realistic spatiotemporal patterns of albedo with unrealistically noisy input predictors.

For these ablation studies, either a large amount of noise is injected into predictor variables to model physically unrealistic perturbations, or they are set to the minimum value of each predictor variable to simulate a physically realistic perturbation. These perturbations are applied successively & independently to each variable, accounting for variable interactions within the model by tracking growth in the RMSE as predictors are ablated. Figure S2 shows these results. The left panel first indicates that replacing predictor variables with the minimum, which corresponds to zero in the normalized space, leads to a large degradation in model performance when latitude is removed. For noise injection, the model degrades most when noise is added to the variables related to liquid clouds, snow fractional area coverage, the previous month’s albedo, and aerosol optical depth. The noise injection ablation shows that variables that, through known, well-established radiative processes of reflection and scattering, strongly influence albedo instantaneously, are also important for the U-Net model predictive skill. They show that the model has captured the salient radiative processes that influence albedo, while also providing unique insights into their temporal and spatial correlation structure. When ablation involves the rearrangement of spatio-temporal features, as shown in the right panel of Fig. S2, we see that rearranging the regions used to predict albedo as a function of latitude and longitude (grid) and the regions where albedo is predicted (target), degrades model performance (increase RMSE). Additionally, changing the time-lag at which albedo is predicted produces a very small impact on the model’s RMSE, indicating that the model finds that the processes that predict spatial patterns in albedo are temporally stationary.

## Supplementary information

Transparent Peer Review file

Supplementary Information

## Data Availability

As noted in the Methods, the Data used for this manuscript are publicly available in the following locations: • Satellite imagery for Fig. 1: NASA MODIS Aqua/Terra true color corrected reflectances from NASA WorldView: https://shorturl.at/EWpZU• NASA Blue Marble Base Map for Fig. 1: https://science.nasa.gov/earth/earth-observatory/blue-marble-next-generation/base-map/• CERES EBAF and ancillary datasets: https://ceres-tool.larc.nasa.gov/ord-tool/jsp/EBAFTOA421Selection.jsp• CMIP6 albedo: https://doi.org/10.24381/cds.c866074c.

## Code availability

As noted in the Methods, the Data used for this paper are publicly available in the following locations: • Neural Network and U-Net: https://github.com/samuelhkahn/ai-albedo-research.
