# Supplementary material for: Towards a theory for Earth’s albedo stability and hemispheric symmetry in the 21st Century
Source: Commun Earth Environ. 2026 Jul 21;7(1):884. doi: 10.1038/s43247-026-03817-0 (PMC13751292; doi:10.1038/s43247-026-03817-0)
Supplement: Supplementary file 1 [file 43247_2026_3817_MOESM1_ESM.pdf]

nature portfolio

Peer Review File

Towards a Theory for Earth's Albedo Stability and Hemispheric Symmetry in the 21st Century

Corresponding Author: Dr Daniel Feldman

This file contains all reviewer reports in order by version, followed by all author rebuttals in order by version.

Version 0:

Reviewer comments:

Reviewer #1

(Remarks to the Author)
Review of "Towards a Theory for Earth's Albedo Stability and Hemispheric Symmetry in the Anthropocene", by D. R. Feldman et al.

This paper discusses the phenomena of a nearly-invariant planetary albedo, and essentially indistinguishable northern and southern hemisphere albedo, that are evident in satellite observations from recent decades. The paper sets forth criteria for establishing a working theory to explain these phenomena, and presents two hypotheses and their observational corollaries which can be tested. Using artificial intelligence (AI) methods, the authors then perform some preliminary testing of the two hypotheses, finding some support for the hypothesis that the observed albedo phenomena can be explained by intrinsic Earth system properties that have been invariant during the Anthropocene.

I enjoyed reading this paper and find it to be a useful contribution that will hopefully help to catalyze effort within the scientific community to better understand these phenomena, which is important (as the authors note) for predictive capability. I have just a few additional comments below that should require only minor revisions to the manuscript.

Additional comments:

1) I feel that the manuscript as currently written is a bit too dismissive on the potential utility of traditional climate models for helping to advance our understanding of these albedo phenomena. The authors try to make the case that these models are not generally useful for this purpose because they are tuned, and they note persistent biases in the models' albedo simulation (lines 126-127). I am honestly not particularly convinced by the model tuning argument. First of all, it is not the case that models are tuned to get the present-day albedo, as is stated at one point (line 272). If that was the case, the models would have the same albedo as CERES, yet Figure 3 indicates that that is clearly not true. Second, while it may be true that models are tuned to get the present-day global energy balance (as stated elsewhere in the manuscript), I don't think this precludes their use for examining how the global energy balance (including albedo) may respond to forcing. Indeed, this has been done routinely in the community for decades now in model-based climate sensitivity studies. Regarding the issue of persistent biases in the models' albedo simulation that is noted, I think it would be helpful if the authors could elaborate on this a bit, rather than just citing previous work. The reason I say this is because looking at Figure 3, it doesn't seem to me that the models are doing too bad. For the planetary albedo (Fig. 3a), the CERES observations are a bit lower than the multimodel mean, but still well within the model spread. For the hemispheric albedo difference (Fig. 3b), the observations are close to the multimodel mean. So, which aspects of the observed albedo do the models struggle to capture? In summary, I feel that the manuscript should present a more balanced view of the potential utility of traditional climate models for tackling this problem. Certainly these models are not perfect, and perhaps it is the case that model biases limit their utility in some regards, but I still believe that they have more potential value for addressing this problem than is currently conveyed in the manuscript. For example, can these models provide any insight into the albedo response to stratovolcanic eruptions? What about their value in helping to better understand observed albedo changes over recent decades? Looking at Figure 3a, the observed ~1% decrease in planetary albedo that is evident in CERES appears similar to the simulated albedo decrease during this time in several models. Does the model-simulated decrease occur for the same reason as the observed decrease? In closing, this rather lengthy comment should not be taken as an objection to the use of AI methods for tackling this problem, as the authors have advocated. I can certainly see the potential value of these methods. I'm simply arguing that there's a role for traditional climate models, too.

2) There are a couple of places in the manuscript where specific AI methods are mentioned and the reader is referred to the SI Appendix for details (lines 179-180, 182). However, these details are actually provided in the Methods section of the paper, not in the SI Appendix. Also, in the Supplementary Information, Figure S1 appears twice (i.e., the same figure is duplicated). Thus, the Figure S2 that is discussed in the text does not appear.

3) I feel that a couple of the figure captions are too brief and not informative enough, in particular for Figure 4 and Figure S1. For Figure 4, how do I interpret a positive versus a negative SHAP value? For both figures, the various acronyms displayed should be defined somewhere, if not in the figure caption then a referral to the Methods section where they are defined.

Reviewer #2

(Remarks to the Author)
Review of "Towards a theory for Earth's albedo stability and hemispheric symmetry in the Anthropocene" for Nature Communications.

In this paper the apparent stability of Earth's planetary albedo and corresponding hemispheric asymmetry is discussed with an overall aim of building a theory for this apparent phenomenon. The evidence presented is results from a machine learning algorithm applied to the CERES record assisted by some additional datasets. It is also suggested that a future volcanic eruption will advance our knowledge.

I cannot help to feel that the paper discusses basic theory- and hypothesis-building at length, and in a tone that seems to devalue past work, but fails to deliver compelling evidence to support it. By default, any machine learning algorithm is opaque to the readership of a paper such as this, and so the algorithm will have to earn credibility, to a larger extent than something more familiar, such as multi-linear regression or other basic statistics. Furthermore, I was surprised that the section on volcanic eruptions did not take advantage of Pinatubo, which was observed by ERBE, and if nothing else the authors should refer to studies that did. The results have varied.

I would recommend the authors tone down their writing style, and rather than spending a lot of time on how theory may be formed, focus on what the data and machine learning algorithm says and provide the support needed to build confidence in that. In plain terms, put the horse in front of the cart. I have my doubts this can be done as part of a regular major revision.

Here I provide some detailed comments, though I note that addressing these alone will not satisfy the above expressed concerns:

'Anthropocene' is a somewhat controversial geological era definition. I would recommend not using it here since it seems irrelevant as we are anyway limited to instrumental records. After all, the goal of a theory on Earth's albedo should not depend on humans influence on sediments, and also the term is controversial. I would recommend removing all references to this era.

37-38, somewhat repetitive

83-85, this statement was a bit upsetting. It sounds like zero progress has been made before, and the authors know exactly why?

113, I wouldn't use 'very narrow range' to describe this

118-121, rather irrelevant, one is a feedback, the other a forcing.

122-128, this is a postulate, one could also argue that with more knobs to tune, more data and time on their hands models can improve their skill. I am also not sure how this paragraph is relevant?

129, 'are unknown' seems to imply that zero progress has been made so far?

131, of course climate models are built on theory

151, 'cooling' -> 'cloud radiative effect'

152-157, please take time to explain how you derived these corollaries.

160-161, not sure I understand how 'because clouds have multiple degrees of freedom' should mean that they stabilise Earth's albedo? They could also pose a positive shortwave feedback (which is the mainstream assessment, e.g. IPCC AR6, Sherwood et al. 2020), which would make it change faster.

164, that a volcanic eruption that injects stratospheric aerosols reduce the cloud radiative effect is trivial as less sunlight will reach the clouds, and will happen without clouds responding at all.

173-175, again a quite upsetting statement, please moderate

185, change to 'to identify the spatial'

187-192, the way this is written, I get the sense that the FFNN assumes that mechanisms are stationary. If that is the case, then I don't think it can be used to test whether it is.

Figure 4, please improve this figure. First all acronyms must be explained. But then remember that most readers, including me, will have little familiarity with the method applied and will not know what the SHAP value represents. I would suggest instead of wasting time on Figure 1+2, to use this space for pedagogically working your way to this figure.

199-218, this is a very long paragraph with many results presented, in fact most of the actual evidence presented in the paper. Please improve the readability of this central part.

217, it is unclear what 'these' refers to. Are all your results just for illustration?

280, it is not true that these are 'distinct', planetary albedo is completely dominated by cloud feedbacks, and trying to distinguish the lines of research is most likely counterproductive. Also, I am not sure I would use the words 'albedo science'.

Version 1:

Reviewer comments:

Reviewer #1

(Remarks to the Author)
I am generally satisfied with the authors' responses to my concerns on the original manuscript and with the revisions that they have made. I can recommend that the revised manuscript be accepted for publication.

Reviewer #3

(Remarks to the Author)
Overview:

If I understand correctly, this manuscript is submitted as an original-research article, not a perspective or commentary. The distinction is that research articles report specific new advances in a field, while perspectives give general high-level arguments aimed at shaping how the community thinks about the field as a whole. This manuscript feels like a hybrid: large tracts (lines 63-115 and Figs 1 & 2) are devoted to very general, high-level discussion that has more the feel of a big-picture perspective think piece. Every paper needs an introduction providing a background to frame the new results, but this is much more general and extensive than usual. After such a portentous spin-up, one would expect the paper to deliver a startling new research result that reshapes our general thinking about Earth's albedo. But the paper underdelivers---it sets out a couple of very general, in my view underspecified hypotheses, then goes into some very technical, opaque application of machine learning (ML) that so far as I can see doesn't really test the hypotheses in a strong, direct sense, and ends with some more very general discussion. In the end, I'm not sure what I have learned from the paper or how it changes my thinking.

Nonetheless, the application of ML to the albedo problem is interesting, and I think the paper could become a readable and useful contribution with some additional work. I suggest (i) cutting to the chase: remove most of the high-level discussion and get to the point more quickly (ii) sharpen up the hypotheses (iii) spend a bit more time laying out the ML methods and giving more of an intuitive feel for how to interpret their results (iv) apply them to actually test the hypotheses in a more clear and conclusive way.

Specific comments:

1. The "infographic" in Fig 1 doesn't really provide much new info to the presumably well informed readership of a disciplinary research article like this. Fig 2 provides a basic schematic of Karl Popper's falsification logic, which again is something most scientists learn in graduate school and doesn't need repeating unless there's a very good reason to (which I'm not seeing here). So suggest removing these figures and associated text to gain space for other, more novel material later on.

2. l.29, unexplained phenomena: these two facts are the central focus of the paper. I'm happy with citing fact 2 as an important unexplained fact: it is indeed surprising that

albedo is hemispherically symmetric given the large, permanent asymmetries in continents, aerosol loading, ocean currents etc between the two hemispheres. Fact 1 seems more problematic. What do you mean exactly by "planetary albedo appears to be stable"?
- Firstly, by "planetary" do you mean "globally averaged"? When discussing albedo, the expression "planetary albedo" is usually taken to be interchangeable with "Bond albedo", ie. the ratio of TOA reflected to incident insolation. Please clarify here and throughout when you mean Bond albedo and when you mean globally-averaged albedo, and use the terms consistently.
- Secondly, what do you mean by "stable"? Do you mean that the global-mean albedo varies little over time? Fig 3 shows that observed albedo does vary in time---there's a clear increase corresponding to Pinatubo, some minor wiggles later on, and an overall decreasing trend over the record. Is this variability small? Small compared to what? If the climate system were in perfect steady state, we would in fact expect albedo to be constant on some long-term average. So please be more precise as to what phenomenon you are referring to and why it is surprising and needing an explanation.

3. l.95: Why does the theory need to apply only to the 21C and not other centuries? Surely the laws of physics are time invariant, so a good theory for the 21C should apply to any other period in which the climate system is subject to large radiative forcing, whether it is anthropogenic or otherwise. Please clarify.

4. l.118-133: Actually I find it surprising and interesting that the CMIP6 mean matches the observations so closely (Fig 3a). This seems like an interesting result that would merit more discussion. In particular, it the model-mean seems to capture the overall negative trend over the observational period, and the projections show that this trend simply continues to the end of the 21C. So the models suggest that global-mean albedo is not actually stable, but decreases in response to anthropogenic forcing? In fact, this decrease is consistent with the sea ice and snow retreat, increased SW absorption due to increasing water vapor, and negative SW cloud feedbacks that we generally expect in a warming climate. So how do the model results stack up against your "unexplained phenomena" and the hypotheses presented later on? I suggest expanding discussion of these aspects and working them in to the discussion of your hypotheses later on.

5. l.146-162: I have a hard time understanding what you mean by "intrinsic properties". The sentence "Earth's top-of-troposphere planetary albedo and its hemispheric symmetry are the result of properties of the Earth system at the surface and in the troposphere that are invariant on the time-scales of the 21st Century" makes some sense --- I guess you are saying that albedo is *not* strongly affected by things that will change over the 21C, like CO_2 and aerosol loading. But CO_2 and aerosols affect clouds and water vapor, which are known to affect albedo. In fact it is very hard to think of anything in the climate system that will stay strictly invariant in the face of 21C radiative forcing. One thing we can be sure won't change is the layout of continents, but this is highly asymmetric across hemispheres and can't be used as a basis for explaining the hemispheric symmetry of albedo. So I am very confused about the precise content of your hypothesis. For example, do you mean to say that albedo itself is an intrinsically invariant property of the climate system, which does not change however much one changes CO_2 or aerosols? Moreover, what exactly is the physical content of your hypothesis? For example, what possible mechanisms make Bond albedo be insensitive to surface albedo, as you claim in at l. 170?

6. l.178-193: Cloud buffering hypothesis. This hypothesis has more physical content, in that it points to a specific mechanism by which the climate system may arrange itself to maintain roughly constant albedo. In this sense, it seems like a more precise and specific version of your previous hypothesis (but I am unsure about this since I don't understand your "intrinsic" hypothesis). In any case, isn't the hypothesis immediately falsified by the observation that Bond albedo increases a lot during Pinatubo? I note that you cite Gristey and Feingold 2025 in support of your hypothesis (l.185). But if I understand that paper correctly, it actually says the opposite: it says that cloud scattering (and therefore cloud albedo, and thus SW CRE) actually *increases* in the presence of stratospheric aerosol, instead of decreasing as your hypothesis requires (I quote from their abstract: "diffusion of the radiation field that would accompany stratospheric aerosol injection can result in sunlight entering clouds at steeper angles, which increases cloud albedo without actively injecting aerosols into clouds.")

7. As discussed in 5 and 6, the hypotheses as they stand seem hazy and underdetermined to me. I suggest sharpening them up and possibly dropping the "intrinsic" hypothesis. The cloud buffering hypothesis on the other hand sounds interesting and could be a good case for testing with you ML approach.

8 l.194-221: ML methods. ML is relatively new to climate science, and needs to be introduced with some care and attention that the reader understands what is being done and how results should be interpreted. The material under the methods section unfortunately reads to my untrained eye like a jargon word salad. Please rewrite in more user-friendly language (where "user" in this case means "climate scientist with very little previous contact with ML", which is the majority of the community at the current stage), and provide some intuitive insight into how the methods work and how they are different from standard regression. In particular you are using SHAP, which if I understand correctly is an attempt to make ML interpretable; if so, please spend a bit more time explaining *how* it should be interpreted.

9. Fig 4. It's interesting that linear regression indicates a weak role for latitude, while ML indicates a leading role. Climatologically, albedo has a big meridional temperature gradient (as shown in Fig 1), so I would expect even linear regression to pick that up. Providing more insight into reasons for the difference between these two methods would give a better sense for the value added by the ML approach.

10. It doesn't seem to me that your ML application actually tests the hypotheses you propose. Why not do a more direct test --- for example, you could look at correlations between surface albedo and cloud albedo to directly test you cloud buffering hypothesis, or put another way, check if your ML method predicts decreased cloud albedo when surface albedo increases. Along the way it might turn out that simple linear regression does not capture this correlation---if so, that would go a long way to buttressing your case that ML have a useful and unique role to play in understanding Earth's albedo.

Version 2:

Reviewer comments:

Reviewer #3

(Remarks to the Author)
The authors have responded very thoroughly to my previous comments and I find the paper much improved. In particular the new results regarding the cloud buffering hypothesis are very interesting. The general presentation and framing of the hypotheses is also a lot clearer now. I can recommend the paper for acceptance in its current form.

Open Access This Peer Review File is licensed under a Creative Commons Attribution 4.0 International License, which permits use, sharing, adaptation, distribution and reproduction in any medium or format, as long as you give appropriate credit to the original author(s) and the source, provide a link to the Creative Commons license, and indicate if changes were made.
In cases where reviewers are anonymous, credit should be given to 'Anonymous Referee' and the source.
The images or other third party material in this Peer Review File are included in the article's Creative Commons license, unless indicated otherwise in a credit line to the material. If material is not included in the article's Creative Commons license and your intended use is not permitted by statutory regulation or exceeds the permitted use, you will need to obtain permission directly from the copyright holder.
To view a copy of this license, visit https://creativecommons.org/licenses/by/4.0/

Dear Editor,

We thank the two Reviewers for their time and effort to review our manuscript “Towards a Theory for Earth’s Albedo Stability and Hemispheric Symmetry in the 21st Century” for *Communications Earth and Environment* so thoroughly and similarly thank your office for your time and effort here. We appreciate the invitation from your office to resubmit our manuscript following their suggested revisions. We provide a point-by-point response (in plain text) to each comment (in blue italics) as well as a revised manuscript that specifically addresses all of the referees' concerns.

Reviewer #1 (Remarks to the Author):

Review of “Towards a Theory for Earth’s Albedo Stability and Hemispheric Symmetry in the Anthropocene”, by D. R. Feldman et al.

This paper discusses the phenomena of a nearly-invariant planetary albedo, and essentially indistinguishable northern and southern hemisphere albedo, that are evident in satellite observations from recent decades. The paper sets forth criteria for establishing a working theory to explain these phenomena, and presents two hypotheses and their observational corollaries which can be tested. Using artificial intelligence (AI) methods, the authors then perform some preliminary testing of the two hypotheses, finding some support for the hypothesis that the observed albedo phenomena can be explained by intrinsic Earth system properties that have been invariant during the Anthropocene.

I enjoyed reading this paper and find it to be a useful contribution that will hopefully help to catalyze effort within the scientific community to better understand these phenomena, which is important (as the authors note) for predictive capability. I have just a few additional comments below that should require only minor revisions to the manuscript.

Additional comments:

1) I feel that the manuscript as currently written is a bit too dismissive on the potential utility of traditional climate models for helping to advance our understanding of these albedo phenomena. The authors try to make the case that these models are not generally useful for this purpose because they are tuned, and they note persistent biases in the models’ albedo simulation (lines 126-127). I am honestly not particularly convinced by the model tuning argument. First of all, it is not the case that models are tuned to get the present-day albedo, as is stated at one point (line 272). If that was the case, the models would have the same albedo as CERES, yet Figure 3 indicates that that is clearly not true. Second, while it may be true that models are tuned to get the present-day global energy balance (as stated elsewhere in the manuscript), I don’t think this precludes their use for examining how the global energy balance (including albedo) may respond to forcing. Indeed, this has been done routinely in the community for decades now in model-based climate sensitivity studies. Regarding the issue of persistent biases in the models’ albedo simulation that is noted, I think it would be helpful if the

authors could elaborate on this a bit, rather than just citing previous work. The reason I say this is because looking at Figure 3, it doesn't seem to me that the models are doing too bad. For the planetary albedo (Fig. 3a), the CERES observations are a bit lower than the multimodel mean, but still well within the model spread. For the hemispheric albedo difference (Fig. 3b), the observations are close to the multimodel mean. So, which aspects of the observed albedo do the models struggle to capture? In summary, I feel that the manuscript should present a more balanced view of the potential utility of traditional climate models for tackling this problem. Certainly these models are not perfect, and perhaps it is the case that model biases limit their utility in some regards, but I still believe that they have more potential value for addressing this problem than is currently conveyed in the manuscript. For example, can these models provide any insight into the albedo response to stratovolcanic eruptions? What about their value in helping to better understand observed albedo changes over recent decades? Looking at Figure 3a, the observed ~1% decrease in planetary albedo that is evident in CERES appears similar to the simulated albedo decrease during this time in several models. Does the model-simulated decrease occur for the same reason as the observed decrease? In closing, this rather lengthy comment should not be taken as an objection to the use of AI methods for tackling this problem, as the authors have advocated. I can certainly see the potential value of these methods. I'm simply arguing that there's a role for traditional climate models, too.

<u>Response:</u>
We appreciate Reviewer 1's thorough review of our submission and thank the Reviewer for raising these important issues, including advocating for a path forward for albedo theory that leverages the capabilities of climate models.

The original manuscript should have properly characterized the tuning process as focused on, among other optimizations, the Earth's top-of-atmosphere net (SW + LW) radiation, instead of just being focused on albedo. The manuscript has been revised (Line 46) to indicate the TOA energy imbalance as a tuning target and how this may impact albedo.

"This net-radiation tuning adjusts under- or over-constrained processes that impact outgoing longwave radiation (OLR) and albedo,and can therefore influence the albedo values within each model at least during its simulations of the historical period (Mauritsen et al 2012; Golaz et al 2013; Hourdin et al 2017; Schmidt et al 2017). It remains unclear how this tuning impacts the predictive value of albedo from models for future projections."

As Reviewer 1 indicates, there can be value in having an ensemble of CMIP simulations together as they depict a range of albedo magnitude, trends, and variability on annual to decadal time-scales. Fundamentally, the ensemble represents a numerical expression of the scientific community's understanding of this quantity. The agreement of the multi-model mean to within less than 1/2 standard deviation of the CERES EBAF observational product indicates that, on average, the scientific community's understanding and ability to model this field is unbiased, even if an individual model might be. Furthermore, the multi-model mean time-series over the historical period roughly tracks the observed decline in albedo, although since the length of the CERES EBAF is only a few decades, we cannot rule out that the agreement in

trends between the multi-model mean and EBAF may be coincidental. The cause(s) of this emergent agreement between the CMIP ensemble and EBAF is an interesting topic in and of itself, though it is beyond the scope of this manuscript. Nevertheless, we agree that it is reasonable to posit that the multi-model ensemble performance provides justification for its use to gain predictive understanding of planetary albedo. What that means is that coordinated experiments across the multi-model ensemble may be valuable for predicting and exploring the response of the Earth's albedo to likely perturbations. It also means that it may be difficult to interpret isolated experiments that utilize a single or small subset of models to predict and explore the Earth's albedo.

In point of fact, Figure 3 shows that the absolute magnitude of albedo for a given model can vary between ~0.28-0.32. This amounts to a range in reflected shortwave radiation of 14 Wm-2 which is much larger than the tuning target of +/-1-3 Wm-2.

The revised manuscript has been changed to the following (Lines 118-141):

"Partially due to climate model tuning (Mauritsen et al, 2012; Golaz et al, 2013; Hourdin et al, 2017; Schmidt et al, 2017), climate models exhibit a range in their TOA planetary albedo fields over the historical period (prior to 2015). The multi-model ensemble mean follows the observed albedo from CERES and also roughly follows the albedo field that was reconstructed prior to the start of the CERES record (see Supplemental) (Allan et al, 2014)). However, these climate models exhibit a much larger range in their projections of planetary and hemispheric albedo change in the 21st Century. This is shown in Figure 3 which analyzes the output from 40 different models from the Coupled Model Intercomparison Project - Phase 6 (Eyring et al 2016) both for their historical simulations and their simulations of 21st Century albedo with the Shared Socioeconomic Pathway 5 with 8.5 W/m^2 radiative forcing relative to pre-industrial conditions (SSP5-8.5) (O'Neill et al, 2016). The range of albedo projections for the 21st Century corresponds to over 2.5 W/m^2 of shortwave radiation that is available, or not, to the Earth's climate system at the end of the 21st Century depending on which climate model's projections is used.

As the advancement of climate models pushes climate models to greater complexity, the biases that the climate models exhibited in their simulated top-of-atmosphere albedo relative to CERES observations have persisted (Jian et al, 2020), as have the magnitude of hemispheric albedo differences (Rugenstein et al, 2021). The reasons for this persistence are still being investigated. For planetary averages, there may be issues with model representations of aerosols (Hodenburg et al, 2024) as well as challenges they face with the apparently changing role of clouds in contributing to albedo (Stephens et al, 2022; Goessling et al, 2025). For hemispheric symmetry, several reasons have been identified including (1) how realistic the models are in terms of reflected shortwave radiation in midlatitudes, and (2) the representation of cloud responses to coupled ocean-atmosphere processes (Jonsson et al, 2022).

At the same time, the skill of the multi-model ensemble mean must be recognized to reproduce observations, including over the historical period (prior to 2015) and even since 2015 when the models were forced by scenario projections. This skill indicates that experiments with an ensemble of different models may support theory development, even if individual models may not. Figure 3 shows how closely this mean tracks the CERES-observed albedo over time both for planetary averages and hemispheric differences. The models notably track recent observed declines in albedo that appear to be, in part, due to aerosols (Hodneburg et al, 2024). Figure 3 also shows that the response of models to the Mt. Pinatubo stratovolcanic eruption was at least moderately realistic, even if the magnitude of the modeled perturbation was lower than observed and relaxation times of that perturbation were longer than observed."

The point raised by Reviewer 1 regarding modeled response to stratovolcanic eruptions is well-taken and is explored in the revised manuscript. We compare a historical, planetary-averaged reconstruction of albedo from DEEP-C with CMIP6 historical simulations (revised Figure 3) and show that models capture the same general impact of Mt. Pinatubo, but that they underestimate the observed increase in albedo and overestimate the length of time that that stratovolcanic eruption enhanced planetary albedo.

In terms of value in understanding changes over recent decades, recent papers by Raghuraman et al, 2021, doi:10.1038/s41467-021-24544-4; Stephens et al, 2022, doi:10.1098/rspa.2022.0053; Hodnebrog et al, 2024, doi:10.1038/s43247-024-01324-8; and Mauritsen et al, 2025, doi:10.1029/2024AV001636 dissect the impact of forcings and feedbacks to help understand Earth Radiation Budget variability using measurement-model intercomparisons. This is challenging to do with observations alone, and those papers indicate that a holistic process understanding is still being investigated. Certainly the model hemispheric symmetry phenomenon for individual models is highly problematic (see Rugenstein and Hakuba, 2023, doi:10.1029/2022GL101802 Fig 1), but again, the ensemble exhibits skill and this should be explored in future work.

2) There are a couple of places in the manuscript where specific AI methods are mentioned and the reader is referred to the SI Appendix for details (lines 179-180, 182). However, these details are actually provided in the Methods section of the paper, not in the SI Appendix. Also, in the Supplementary Information, Figure S1 appears twice (i.e., the same figure is duplicated). Thus, the Figure S2 that is discussed in the text does not appear.

<u>Response:</u>
The manuscript has been revised to refer to the Methods section for specific AI methods (Line 200 and 216). Also, we apologize for the oversight in Figure 2 of the Supplementary Material. It has been fixed and is now included in the revised manuscript's Supplementary Material.

3) I feel that a couple of the figure captions are too brief and not informative enough, in particular for Figure 4 and Figure S1. For Figure 4, how do I interpret a positive versus a

negative SHAP value? For both figures, the various acronyms displayed should be defined somewhere, if not in the figure caption then a referral to the Methods section where they are defined.

Response:

Figure 4 in the original manuscript is called a beeswarm SHAP plot. The beeswarm plot is designed to display an information-dense summary of how the top features in a dataset impact the model's output. Each instance of the given explanation is represented by a single dot on each feature row. The x position of the dot is determined by the SHAP value of that feature, and dots "pile up" along each feature row to show density. Color is used to display the original value of a feature. The absolute value of SHAP value determines its importance, so, like correlation coefficients, both very positive and negative values indicate a feature's importance, while values close to zero do not.

However, we recognize that the beeswarm plot contains too much information for the purposes of highlighting the rank-order of feature importance. Figure 4 has been changed in the revised manuscript to show the mean absolute SHAP values in order to avoid the confusion regarding positive and negative values of a 'beeswarm' SHAP plot, and the captions of the figures have been changed so that it is accessible to the reader.

Additionally, the caption in Figure S1 has been updated to explain the U-Net architecture in greater detail.

Reviewer #2 (Remarks to the Author):

Review of "Towards a theory for Earth's albedo stability and hemispheric symmetry in the Anthropocene" for Nature Communications.

In this paper the apparent stability of Earth's planetary albedo and corresponding hemispheric asymmetry is discussed with an overall aim of building a theory for this apparent phenomenon. The evidence presented is results from a machine learning algorithm applied to the CERES record assisted by some additional datasets. It is also suggested that a future volcanic eruption will advance our knowledge.

I cannot help to feel that the paper discusses basic theory- and hypothesis-building at length, and in a tone that seems to devalue past work, but fails to deliver compelling evidence to support it. By default, any machine learning algorithm is opaque to the readership of a paper such as this, and so the algorithm will have to earn credibility, to a larger extent than something more familiar, such as multi-linear regression or other basic statistics. Furthermore, I was surprised that the section on volcanic eruptions did not take advantage of Pinatubo, which was observed by ERBE, and if nothing else the authors should refer to studies that did. The results have varied.

I would recommend the authors tone down their writing style, and rather than spending a lot of time on how theory may be formed, focus on what the data and machine learning algorithm says and provide the support needed to build confidence in that. In plain terms, put the horse in front of the cart. I have my doubts this can be done as part of a regular major revision.

<u>Response:</u>

We appreciate Reviewer #2’s concerns regarding the tone of the original manuscript, and recognize that it is critical that the paper strike a tone that embraces the scientific accomplishments on this topic to date, especially since we would like to use this paper to motivate community activities that advance the state of the science on this topic further. The revised manuscript contains many modifications to its tone and includes many more references to describe previous work on this topic. Specifically, the section on the range of climate model projections (Lines 117-141) has been greatly modified, as have the sections in lines 88-90 and 142-145 that did not recognize the state of the science and important community accomplishments. More references have been added, and the Discussion section (lines 306-344) have also been toned down.

Regarding the machine-learning components of the original manuscript, we appreciate the Reviewer’s concern and the need to expound on this aspect of the paper. We have added a small amount of explanatory material to the main text (Including “AI Methods to Advance Albedo Hypotheses to Working Theories” section and the Discussion on lines 326-333. Furthermore, a

considerable amount of text and figures to the Supplementary Information (especially the Data Model Complexity section) to address this very point.

In this additional material in the revised manuscript, we make the case for why the data model complexity, and by extension, the use of machine learning methods, is an important tool that can help advance albedo science. We do this by recognizing that there is a wide range of data model complexities, and the data models that achieve the greatest amount of predictive skill with out-of-sample predictors and predictands will, after also recognizing the risks of overfitting those models and therefore penalizing model complexity, be better tools for supporting albedo science and theory development.

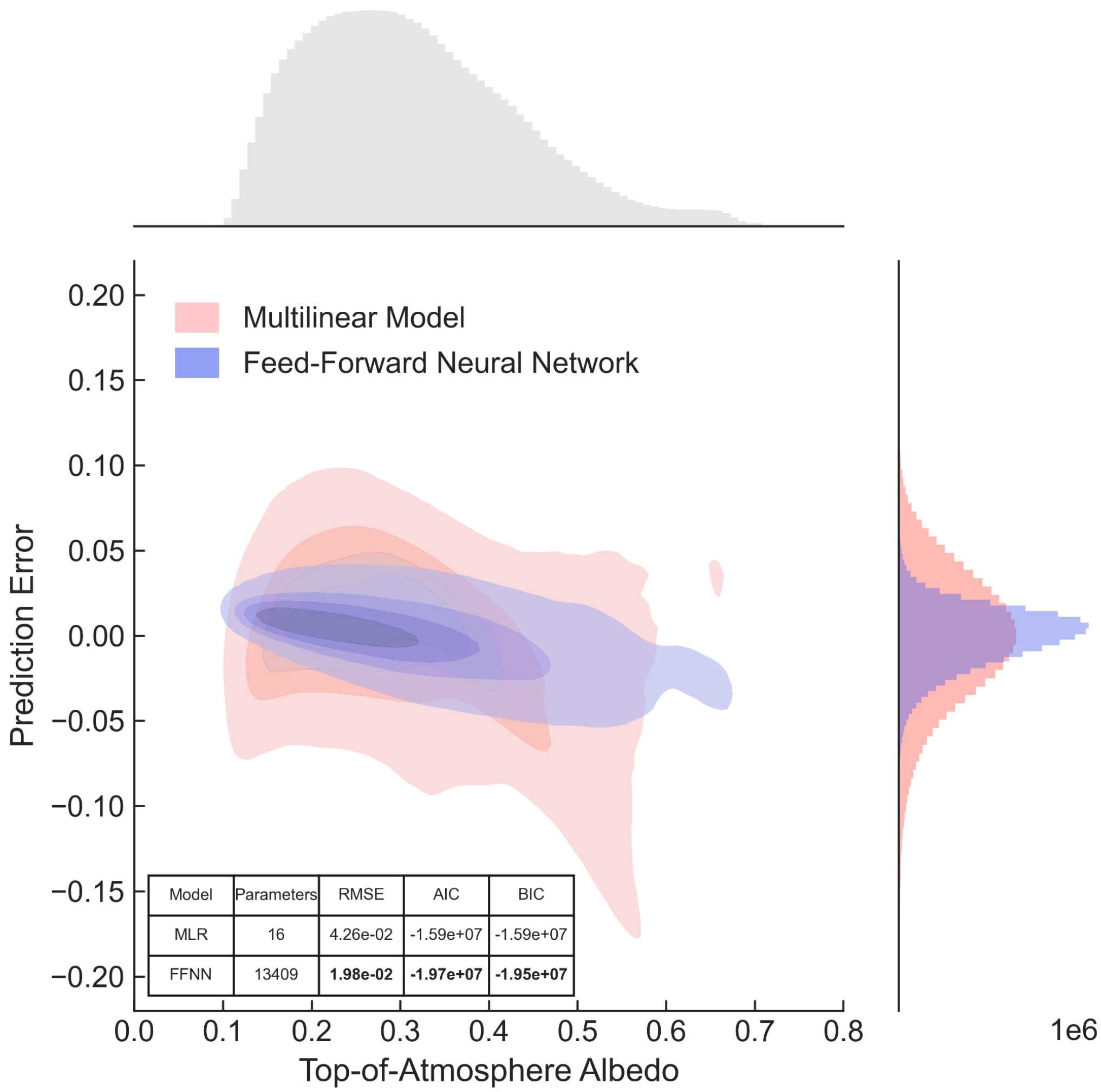

Model	Parameters	RMSE	AIC	BIC
MLR	16	4.26e-02	-1.59e+07	-1.59e+07
FFNN	13409	**1.98e-02**	**-1.97e+07**	**-1.95e+07**

Supplementary Figure 4: Out-of-sample TOA Albedo prediction errors for Multilinear Regression (MLR) and 4 Layer Feed-Forward Neural Network (FFNN) models. The gray PDF at top of plot indicates distribution of EBAF-observed TOA albedo and the red and blue PDFs on the right of plot indicate the distributions of prediction errors for all albedo values with MLR and FFNN models, respectively. The number of model parameters, root-mean squared error (RMSE), the Akaike Information Criterion (AIC) and the Bayesian Information Criterion (BIC) for each data model are shown on the inset table. The better score of the two models is highlighted in bold.

To illustrate this, we have performed an example analysis to intercompare multi-linear methods to neural networks which shows why more advanced, nonlinear methods like machine learning should be strongly considered, even if they are more opaque (Supplementary Figure 4).

This example analysis shows that a multi-linear regression (MLR) model is less optimal than a feed-forward neural network (FFNN) for predicting the CERES EBAF albedo field based on the set of predictor variables included along with the EBAF product. This optimality evaluation is based on a variety of well-established metrics that balance predictive skill and model complexity.

Additionally, there are key theoretical reasons that an MLR model is less optimal than an FFNN that we describe in the Supplementary Information section on Data Model Complexity. Specifically, there are inherent nonlinearities in the radiative transfer and dynamics processes that contribute to albedo. The more complex models, in particular, can capture spatial and temporal patterns from the data. While spatial and temporal features must be engineered in lower-complexity data models such as an MLR, and those features are not known a priori. Simply put, there are many more degrees of freedom in an FFNN data model than an MLR one, and there is an inherently optimal, but currently unknown, number of degrees of freedom needed to support albedo analysis and theory development.

Furthermore, we note that there are differences in the SHAP feature importance identified by these models, and these differences point albedo researchers in different research directions depending on the model complexity chosen. The differences in SHAP feature ordering indicate that the choice of data model complexity does matter for this very problem. The FFNN indicates that the processes that vary meridionally, such as heat transport, insolation, the configuration of continental land masses, and frozen surface extent, may be key to understanding the albedo phenomena and ultimately predicting their evolution in the 21st Century. An MLR approach does not indicate that meridionally-varying processes are as important for the observed albedo phenomena, whereas a FFNN approach - with reduced model prediction error - does.

To be clear, the rank-ordering of feature importance from methods like SHAP analysis does not produce a theory, but it can, with optimal data methods, help guide researchers by providing clues to the underlying nature of albedo teleconnections. We argue that these approaches should therefore be considered by the community as it works towards theory development.

That being said, the FFNN discussed here is presented as an example only. We can say that an FFNN is more optimal than an MLR data model, but should note that an investigation into the optimal data model type and its complexity is needed in the future to address this question thoroughly.

Finally, we thank the Reviewer for raising the issue regarding Mt. Pinatubo. While the original manuscript cited a few papers, it did not include the ERBS observations. We have revised Figure 3a to include an albedo time-series reconstruction that includes ERBS data which is part of the planetary-averaged DEEP-C Version 5.0 dataset (Liu and Allan, 2022) and a TSI dataset

from Greg Kopp (https://spot.colorado.edu/\string~koppg/TSI/). More specifically, the DEEP-C product includes ERBS WFOV version 3.0 data and the TOA flux anomaly is constrained by WFOV anomaly in each 10 degree x 10 degree grid box. We also included a zoomed-in version (from 1985-2025) of Figure 3a as Supplemental Figure 3. We note in the main text, and in more detail in the Supplementary Information, that the CMIP6 models clearly contain a signal from Mt. Pinatubo. That being said, the CMIP6 models underestimate how large and how quickly albedo actually increased in the observationally-reconstructed data and those models also overestimate how long it actually took for the perturbation from Pinatubo to decay as compared to the observationally-reconstruct data.

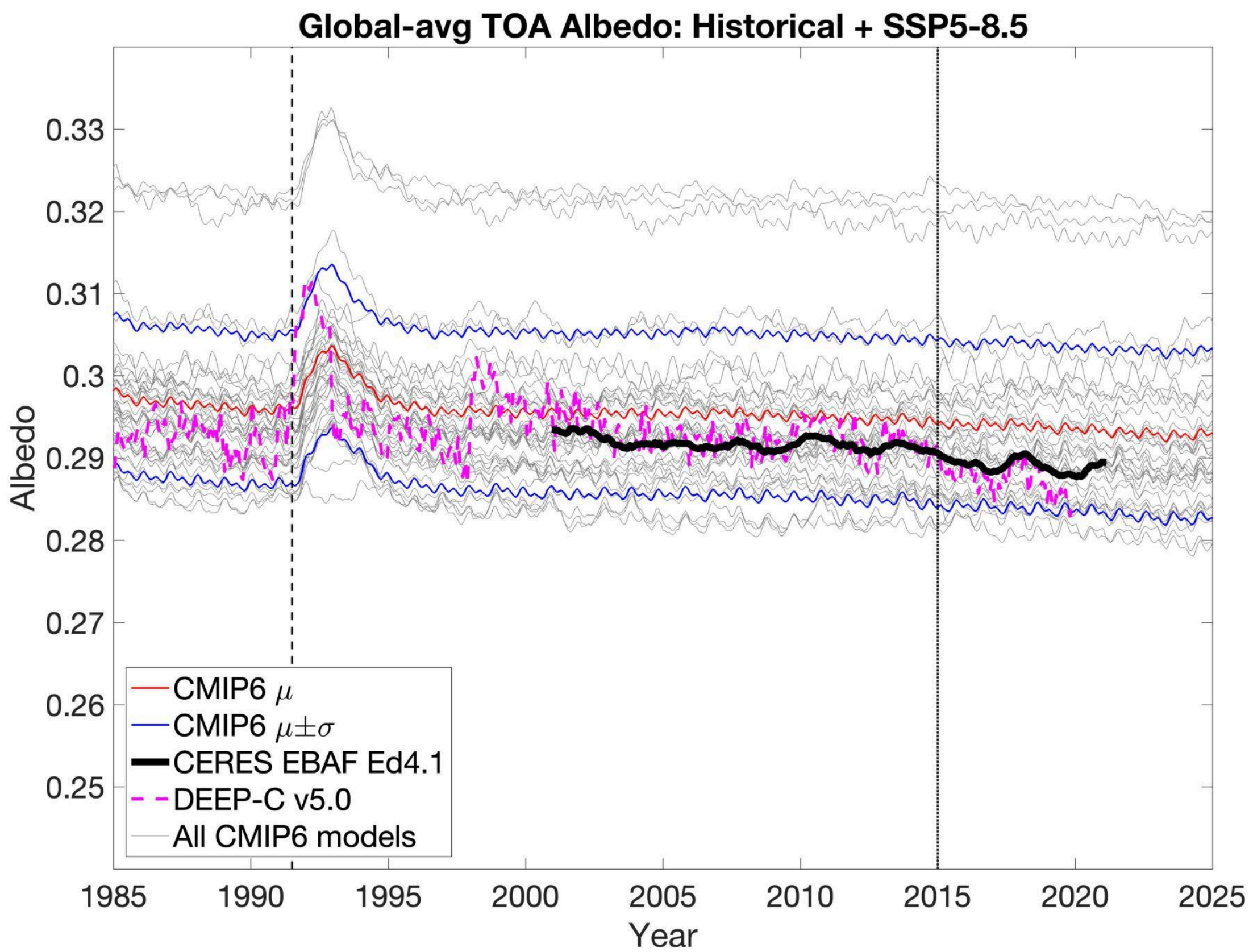

Supplementary Figure 3 in Revised Manuscript: Globally-averaged TOA albedo as measured by CERES-EBAF (black), DEEP-C and Kopp database (pink dashed) and historical simulations and future projections from a suite of CMIP6 models with the multi-model average and standard-deviation depicted. The vertical dashed black line indicates the Mt. Pinatubo stratovolcanic eruption in June, 1991. Vertical dotted line marks end of CMIP6 historical simulations.

Here I provide some detailed comments, though I note that addressing these alone will not satisfy the above expressed concerns:

'Anthropocene' is a somewhat controversial geological era definition. I would recommend not using it here since it seems irrelevant as we are anyway limited to instrumental records. After all, the goal of a theory on Earth's albedo should not depend on humans influence on sediments, and also the term is controversial. I would recommend removing all references to this era.

Response:
Thank you for raising this point. We have removed all instances of the "Anthropocene" in the revised manuscript, recognizing the potential controversy that goes along with it, and replaced them with "the 21st Century".

37-38, somewhat repetitive

Response:
As part of the revision, and also responding to Reviewer #1's comments, this sentence and the previous sentence have been changed, along with adding a recognition of some of the previously-undertaken conceptual model approaches (Lines 37-38).

83-85, this statement was a bit upsetting. It sounds like zero progress has been made before, and the authors know exactly why?

Response:
This statement has been changed in the revised manuscript, especially to recognize the need for a revised tone that Reviewer #2 raised in the above comment. It now reads (Lines 88-90): "This reductive diagnosis is helpful for characterizing past events, but the community is still seeking to understand if the observed phenomena are coincidental or if there are teleconnections that produce these phenomena that are the result of fundamental, and yet-to-be-determined physical mechanisms."

113, I wouldn't use 'very narrow range' to describe this

Response:
This sentence has been changed in the revised manuscript, also per Reviewer #1's comments. It now reads (Lines 118-119): "Partially due to climate model tuning (Mauritsen et al, 2012; Golaz et al, 2013; Hourdin et al, 2017; Schmidt et al, 2017), climate models exhibit a range in their TOA planetary albedo fields over the historical period (prior to 2015)."

118-121, rather irrelevant, one is a feedback, the other a forcing.

Response:
The second sentence has been removed in the revised manuscript. We retain the first sentence to indicate to the reader the energetic implications of the range in CMIP6 albedo projections (Lines 124-126).

122-128, this is a postulate, one could also argue that with more knobs to tune, more data and time on their hands models can improve their skill. I am also not sure how this paragraph is relevant?

Response:
This point made by Reviewer #2 is well-taken and is also related to comments from Reviewer #1 regarding the role of models in supporting albedo science. In the revised manuscript (Lines 127-133), the paragraph has been changed to remove postulating about future models, highlight relevant albedo science using models, and the following paragraph now indicates that science using the multi-model ensemble may be warranted given that ensemble's ability to track EBAF albedo, even outside of the CMIP6 historical experiments (from 2015 onwards).

129, 'are unknown' seems to imply that zero progress has been made so far?

Response:
The manuscript has been revised and toned down. This sentence and the following sentence now read (Lines 142-145): "Ultimately, the physical mechanisms and their connections that lead to the observed phenomena of albedo stability and symmetry remain subjects of active scientific investigation. There is a role for a working theory to describe the emergent properties of top-of-atmosphere albedo in observations and climate models so that that theory can be explicitly represented in such models"

131, of course climate models are built on theory

Response:
This point is well-taken, though top-of-atmosphere albedo is an emergent property of the theoretical principles on which climate models are built. This sentence in the revised manuscript now reads (Lines 143-145): "There is a role for a working theory to describe the emergent properties of top-of-atmosphere albedo in observations and climate models so that that theory can be explicitly represented in such models."

151, 'cooling' -> 'cloud radiative effect'

Response:
Our language here was imprecise. We meant to refer to the mechanism in the Scheider et al, 2019 paper whereby turbulence-driven longwave radiative cooling, which maintains stratocumulus clouds (as described by Wood, Mon. Weather Rev., 2012) can be limited by rising greenhouse gases. This sentence in revised manuscript now reads (Lines 163-166): “At the same time, we recognize that recent observations of albedo suggest that planetary albedo may not be completely invariant on interannual time-scales (Diamond et al, 2023), so modifications to this hypothesis may be required, perhaps to account for the impact of changing atmospheric composition on the turbulence that sustains clouds driven by longwave radiative cooling of the cloud tops (Wood, 2012; Schneider et al, 2019).”

152-157, please take time to explain how you derived these corollaries.

Response:
Explanations of these corollaries have been added to the revised manuscript per the Reviewer’s recommendation on Lines 167-177 of the revised manuscript.

160-161, not sure I understand how 'because clouds have multiple degrees of freedom' should mean that they stabilise Earth's albedo? They could also pose a positive shortwave feedback (which is the mainstream assessment, e.g. IPCC AR6, Sherwood et al. 2020), which would make it change faster.

Response:
We refer the Reviewer to Section 7.2 of Stephens et al, 2015 (doi:https://doi.org/10.1002/2014RG000449) which discuss how interannual variability in RSR is dominated by interannual variability in atmospheric scattering, and also the abstract of that paper that presents the degrees of freedom argument. To be clear, we present this as an example from the published literature, so that its observational corollaries can be explored. At the start of the “Intrinsic Properties Hypothesis” section, we changed the language in the revised manuscript (Lines 147-148) to caveat these two hypotheses with:

“To illustrate a path forward towards theory development, we present two hypotheses and their observational corollaries, though note that these are merely examples and there may be other, superior candidate hypotheses”

164, that a volcanic eruption that injects stratospheric aerosols reduce the cloud radiative effect is trivial as less sunlight will reach the clouds, and will happen without clouds responding at all.

Response:

We concur that there are the direct changes to incident solar radiation at cloud-tops that could impact their radiative effects. However, there are additional considerations beyond this. Stratospheric aerosols change the partitioning of the radiation between direct and diffuse that, in turn, can change the cloud albedo (Gristey and Feingold, 2025, https://doi.org/10.1029/2024GL113914). Also note that the cloud buffering hypothesis suggests that there would be some detectable amount of compensatory changes in cloud scattering and that this compensation would go down over time as stratospheric aerosols themselves decay over time. The revised manuscript now reads (Lines 183-185): “Second, a major stratovolcanic event like Mt. Pinatubo or other perturbations to planetary albedo would, according to this hypothesis, lead to a global, detectable reduction in cloud radiative effects to compensate for the rise in aerosol scattering. Changes in cloud radiative effects could occur due to changes in cloud amount, cloud properties, or changes to the radiation field even for identical clouds (Gristey and Feingold, 2025, https://doi.org/10.1029/2024GL113914). That compensatory effect would also lessen in tandem as aerosol scattering decays.”

173-175, again a quite upsetting statement, please moderate

Response:
We recognize that statement was upsetting and requires revision. The revised manuscript now reads (Lines 196-198): “The scientific community studying albedo may be able to gain additional understanding about the stability and symmetry phenomena with methods such as these, especially because they can discern subtle connections across temporal and spatial scales that may be contributing to these phenomena.”

185, change to 'to identify the spatial'

Response:
The requested change has been made to the revised manuscript (Lines 227).

187-192, the way this is written, I get the sense that the FFNN assumes that mechanisms are stationary. If that is the case, then I don't think it can be used to test whether it is.

Response:
An FFNN, or other model, learns patterns from its training data that ideally are actually the underlying principles of the system being analyzed. Because there is a finite amount of training data used, the FFNN will only have nonstationarities to the extent that the training data also have nonstationary features. At the same time, we did run experiments where we trained on one part of the data record and predicted on another to determine if there is temporal dependence to FFNN skill, and we did not see such a dependence.

Figure 4, please improve this figure. First all acronyms must be explained. But then remember that most readers, including me, will have little familiarity with the method applied and will not know what the SHAP value represents. I would suggest instead of wasting time on Figure 1+2, to use this space for pedagogically working your way to this figure.

Response:
In response to this comment and a similar comment from Reviewer #1, we have revised Figure 4.

Figure 4 in the original manuscript is called a beeswarm SHAP plot. The beeswarm plot is designed to display an information-dense summary of how the top features in a dataset impact the model's output. Each instance of the given explanation is represented by a single dot on each feature row. The x position of the dot is determined by the SHAP value of that feature, and dots "pile up" along each feature row to show density. Color is used to display the original value of a feature. The absolute value of SHAP value determines its importance, so, like correlation coefficients, both very positive and negative values indicate a feature's importance, while values close to zero do not.

However, we recognize that the beeswarm plot is esoteric for the machine-learning community and contains too much information for the purposes of highlighting the rank-order of feature importance, which is really what we meant to convey. Figure 4 has been changed in the revised manuscript to show the mean absolute SHAP values in order to avoid the confusion regarding positive and negative values of a 'beeswarm' SHAP plot.

Additionally, a significant amount of text has been added to describe the FFNN and U-Net.

199-218, this is a very long paragraph with many results presented, in fact most of the actual evidence presented in the paper. Please improve the readability of this central part.

Response:
This paragraph in the revised manuscript has been extensively edited for clarity in order to delineate the findings from the U-Nets and describe their implications (Lines 222-253).

217, it is unclear what 'these' refers to. Are all your results just for illustration?

Response:
The revised manuscript now reads (Lines 261-262): "However, we are by no means confirming the intrinsic properties hypothesis, as more comprehensive testing is needed."

280, it is not true that these are 'distinct', planetary albedo is completely dominated by cloud feedbacks, and trying to distinguish the lines of research is most likely counterproductive. Also, I am not sure I would use the words 'albedo science'.

Response:
This paragraph has been removed from the revised manuscript.

Dear Editor,

We thank the two Reviewers for their time and effort to review our manuscript “Towards a Theory for Earth’s Albedo Stability and Hemispheric Symmetry in the 21st Century” that we submitted to *Communications Earth and Environment* for consideration for publication. The Reviewers examined our manuscript thoroughly and we very much appreciate it. We also thank your office for your time and effort here, and we appreciate the invitation from your office to resubmit our manuscript following the Reviewers’ suggestions and those from your office. Please see the point-by-point response (in plain text) to each comment (in blue italics) below, as well as a revised manuscript that specifically addresses all of the points raised by the Reviewers.

Reviewer #1 (Remarks to the Author):

I am generally satisfied with the authors' responses to my concerns on the original manuscript and with the revisions that they have made. I can recommend that the revised manuscript be accepted for publication.

We thank Reviewer #1 for recommending that our revised manuscript be accepted for publication. We note that there are now no further comments from the original reviewers. Therefore, our responses to Reviewer #3 seek to balance the new requests from Reviewer #3 with our responses to the comments and perspectives of the first two Reviewers so as to create a second revision that is harmonious with all of the comments of the 3 reviewers.

Reviewer #3 (Remarks to the Author):

Overview:

If I understand correctly, this manuscript is submitted as an original-research article, not a perspective or commentary. The distinction is that research articles report specific new advances in a field, while perspectives give general high-level arguments aimed at shaping how the community thinks about the field as a whole. This manuscript feels like a hybrid: large tracts (lines 63-115 and Figs 1 & 2) are devoted to very general, high-level discussion that has more the feel of a big-picture perspective think piece. Every paper needs an introduction providing a background to frame the new results, but this is much more general and extensive than usual. After such a portentous spin-up, one would expect the paper to deliver a startling new research result that reshapes our general thinking about Earth's albedo. But the paper underdelivers---it sets out a couple of very general, in my view underspecified hypotheses, then goes into some very technical, opaque application of machine learning (ML) that so far as I can see doesn't really test the hypotheses in a strong, direct sense, and ends with some more very general discussion. In the end, I'm not sure what I

have learned from the paper or how it changes my thinking.

Nonetheless, the application of ML to the albedo problem is interesting, and I think the paper could become a readable and useful contribution with some additional work. I suggest (i) cutting to the chase: remove most of the high-level discussion and get to the point more quickly (ii) sharpen up the hypotheses (iii) spend a bit more time laying out the ML methods and giving more of an intuitive feel for how to interpret their results (iv) apply them to actually test the hypotheses in a more clear and conclusive way.

We appreciate the thorough review from Reviewer #3 and their detailed feedback. We also are grateful for the opportunity to respond to the points raised here by Reviewer #3, as they are well-taken and we have incorporated their suggestions into the revised manuscript.

First, we wanted to provide a bit of context to Reviewer #3, the other reviewers, and the Editorial Office to explain the structure of the manuscript, which Reviewer #3 rightly describes as including conceptual and higher-level information along with detailed findings. We have been motivated to do the work for this manuscript because: there is

1. A lack of a theory for describing observed albedo phenomena.
2. A lack of a recognition in the published literature for a need for such theory.
3. Minimal information describing paths to get to a theory.
4. No examples of albedo hypothesis testing.

For these reasons, we think it is important to provide a review of the published literature on the state of the phenomenology regarding albedo, formally broach in this manuscript that there is, indeed, a lack of a theory, provide information about why no such theory has been developed to date and also how to get to such a theory.

At the same time, we felt that a manuscript with just information on the first two points listed above would be an opinion piece and as such would only really broach the issues that we see as motivating for this manuscript while not providing meaningful information on how to address what we see as a glaring set of issues. That is why we think it is important to also provide detailed analysis and findings to provide concrete illustrations to the scientific community about how to actually start addressing the issues we raise in the first part of the manuscript.

Together, we recognize that this makes the introduction more extensive than usual, but feel that it is important to include those three missing components to lay the foundation for describing the later sections of the manuscript. As we elaborate on below, we also feel it is important and necessary to include Figures 1 and 2 because of the scope of *Communications Earth and Environment* as a journal, as these help introduce readers who may not be as familiar with the

science of the Earth's energy budget but, most critically, can bring important perspectives and contributions to this topic.

We appreciate Reviewer #3's comment regarding the setup and delivery of the paper. We recognize that with such an introduction, a reader could reasonably at least hope for, or even expect, that a fully-formed theory to describe the described albedo phenomena would be presented, and possibly tested and demonstrated in this manuscript. However, the relatively-limited (several decades) of observations has not been a sufficient constraint to develop such a theory, and we cannot claim that we have achieved it as part of this study.

We can really only claim that there is a viable path to a theory and that:

1. Several hypotheses can be evaluated now and in the future as more observations become available.
2. Stratovolcanic eruptions are a key test for any theory
3. Advanced data methods can provide unique insights into the nature of teleconnections that underlie the albedo phenomenology
4. There may be a role for a multi-model ensemble analyses.

We have added framing language to the introduction to set expectations (revision 2, Line 14, 85-91).

Reviewer #3's suggestion regarding tighter hypotheses presented in the paper is well-taken. We have sharpened especially the previously-described "intrinsic properties" hypothesis and reframed and described it more detail, and we have also provided a smoother on-ramp for discussion on machine learning methods as well as details regarding what is learned from these approaches and the extent to which they are used, or can be used to test hypotheses presented. These changes are reflected in the revision 2, lines 161-191).

Furthermore, we appreciate Reviewer #3's recommendation regarding the technical nature of the ML methods, the associated opacity with them, and the need for language that provides a smooth onramp for the reader. This language has been added to the revised manuscript (revision 2, lines 218-235, 244-258, 264-266).

Finally, we made modifications to the conclusions and discussion in order to show (1) the extent to which our machine learning methods are able to test hypotheses, and (2) how a limited data record relative to resolving the temporal and spatial scales of the processes that contribute to albedo, currently presents challenges to drawing definitive conclusions (revision 2, lines 337-347).

Below, we have specific responses that address Reviewer #3's specific comments.

Specific comments:

1. The "infographic" in Fig 1 doesn't really provide much new info to the presumably well informed readership of a disciplinary research article like this. Fig 2 provides a basic schematic of Karl Popper's falsification logic, which again is something most scientists learn in graduate school and doesn't need repeating unless there's a very good reason to (which I'm not seeing here). So suggest removing these figures and associated text to gain space for other, more novel material later on.

Because *Communications Earth and Environment* has a broad scope and reaches a broad readership, we contend that a figure highlighting the range of processes and scales that contribute to the large-scale albedo phenomena is both an important framing for the remainder of the paper to indicate why there hasn't been a theory developed to date: the time and space scales of the processes that contribute to the phenomenology render the problem inaccessible to facile theory development. Furthermore, we contend that this infographic is central to inducing interest in the topic and potential contributions from the readership to albedo science moving forward. One of our primary reasons for submitting to this journal was that the journal indicates that: "Our scope covers all areas of the geosciences, climate and environmental sciences as well as planetary sciences, including those at the interface with ecology, sustainability and environmental social sciences".

Given this scope, we do <u>not</u> expect the majority of readers that are reached by this journal to have Earth radiation budget science expertise. At the same time, that readership does have science perspectives that are potentially quite valuable for this specific difficult-to-crack Earth science problem. In point of fact, the work we are presenting here is the result of ongoing collaborations between Earth radiation budget science researchers and those with data science backgrounds. The authors with data science backgrounds have been able to more thoroughly probe existing observational datasets for spatial and temporal structures in albedo, atmospheric and surface states than have been previously discussed in the published literature. They have also been able to show that nonlinear methods are necessary for analyzing observations and developing a theory.

Readers of this manuscript with data science and other geoscience, climate, and environmental science backgrounds may not be familiar with the interacting processes and scales of top-of-atmosphere albedo phenomena but can bring their interdisciplinary expertise to bear on the issue with the small amount of framing provided in Figure 1. We have added text to the manuscript to note the importance of interdisciplinary expertise in addressing this issue to the Phenomenology and Scaling Challenges section (revision 2, lines 85-91):

"There are three issues here. First, there is lack of a theory to explain the observations so that additional observations merely lengthen the time-series of recorded albedo without confirming or falsifying a predictive understanding of the phenomena. Second, the complexity of the process interactions may require different approaches and/or advanced data analytic methods that are outside of traditional geoscience approaches. Third, it is unclear the extent to which Earth System Models can assist in advancing this predictive understanding.

Because of these issues, we are working here towards a theoretical framework, analytics, and modeling approaches that address these teleconnections will help predict how or whether these phenomena will evolve in the 21st Century."

Regarding Figure 2, we recognize that it does depict some standard elements of scientific inquiry, which could be seen as retreading well-known topics. At the same time, however, we argue that Figure 2 presents key innovations specific to the problem at hand that are central to the manuscript's approach. Namely, the integration of observational analysis of stratovolcanic eruptions, the role of AI/ML methods for hypothesis development and testing, and the actual mechanisms for testing albedo-related hypotheses are specifics for this scientific inquiry that are unique for albedo science in terms of learning from natural experiments, utilizing information-theoretic methods for observational data processing, and using existing observations while recognizing their range in skill.

Furthermore, there is currently no pathway described in the published literature of which we are aware that depicts how the scientific community can move towards a theory to support the predictive understanding of planetary albedo. We therefore argue that such a figure, albeit rudimentary in some aspects, along with associated background material in the manuscript's introduction, are important to describe as essential infrastructure for a theory, especially as it relates to the remainder of this manuscript and the hopeful impact that this manuscript will have. The readers could easily be confused as to the approach that is being described in the Sections presenting two hypotheses (invariant Earth System and cloud buffering hypotheses) unless we essentially describe what is in the Figure 2 flow-chart as text of the manuscript without having that flow-chart.

Still, Reviewer 3's point that regarding the well-known aspects of Figure 2 is well-taken, and we have added language to the revised manuscript to explain that this is not just the scientific method all over again, but that there are key particulars in Figure 2 relative to a standard scientific inquiry approach as they relate to albedo science (revision 2, lines 121-126).

"There are some elements of this flow-chart that follow traditional elements of scientific inquiry, but we note that are key specifics that are worth highlighting. First, the role of stratovolcanic perturbations to albedo in theory development is paramount. Second, the scope of temporal and spatial scales spanned by a candidate theory is very large, so it must be tested across these scales with advanced analytics. Third, Figure 2 recognizes that the observational data record for albedo is several decades, and a viable theory may require more observations, so the establishment of testable observational corollaries is also paramount."

2. l.29, unexplained phenomena: these two facts are the central focus of the paper. I'm happy with citing fact 2 as an important unexplained fact: it is indeed surprising that albedo is hemispherically symmetric given the large, permanent asymmetries in continents, aerosol loading, ocean currents etc between the two hemispheres. Fact 1 seems more problematic. What do you mean exactly by "planetary albedo appears to be

stable"?
- Firstly, by "planetary" do you mean "globally averaged"? When discussing albedo, the expression "planetary albedo" is usually taken to be interchangeable with "Bond albedo", ie. the ratio of TOA reflected to incident insolation. Please clarify here and throughout when you mean Bond albedo and when you mean globally-averaged albedo, and use the terms consistently.
- Secondly, what do you mean by "stable"? Do you mean that the global-mean albedo varies little over time? Fig 3 shows that observed albedo does vary in time---there's a clear increase corresponding to Pinatubo, some minor wiggles later on, and an overall decreasing trend over the record. Is this variability small? Small compared to what? If the climate system were in perfect steady state, we would in fact expect albedo to be constant on some long-term average. So please be more precise as to what phenomenon you are referring to and why it is surprising and needing an explanation.

While the term “bond albedo” in non-standard in Earth radiation budget science, Reviewer #3 is correct that we are referring to globally-averaged albedo. We have replaced all instances of “planetary albedo” with “globally-averaged albedo.”

We have clarified that we are referring to stability on multi-year time-scales. The stability at multi-year time-scales can be compared with (1) the global mean magnitude averaged over the entire CERES record of ~29%, (2) the spatial variability, essentially spanning from near 0% to near 100%, and (3) the globally-averaged temporal variability on diurnal to seasonal time-scales ranges from 25% to 33%. However, globally-averaged values of albedo over time have been exceedingly stable, with a change of globally-averaged albedo from 29.4 to 28.7% from 2000 to 2025. By any of the preceding measures, global albedo variations on multi-year timescales indicate much smaller variability. To avoid any confusion, we have included these quantities for comparison immediately after the stability is introduced (revision 2, lines 28-30).

3. l.95: Why does the theory need to apply only to the 21C and not other centuries? Surely the laws of physics are time invariant, so a good theory for the 21C should apply to any other period in which the climate system is subject to large radiative forcing, whether it is anthropogenic or otherwise. Please clarify.

We concur with the Reviewer that a theory should apply across more than just in the 21st Century, and, ideally cover other past, present and future periods of the Earth’s climate system with larger or smaller radiative forcing. At the same time, the limited set of albedo observations that exist have only been collected over the past several decades. Therefore, they do not encompass the range of Earth system states that would be expected to majorly influence top-of-atmosphere albedo.

Indeed, models indicate that these vastly different Earth system states (e.g., snowball earth or the Paleocene-Eocene Thermal Maximum (PETM)) can strongly influence this albedo and produce values that differ dramatically from present-day conditions e.g., Hoffman and Schrag,

2002, doi: https://doi.org/10.1046/j.1365-3121.2002.00408.x; Caldeira and Kasting, 1992, doi: https://doi.org/10.1038/359226a0 calculated a planetary albedo of more than 0.6 for snowball Earth while Heinemann, 2009, ISSN 1614-1199 calculated a planetary albedo of less than 0.26 for the PETM).

The albedo of the Earth could have been very different in past ice ages and under much warmer conditions and it could eventually be higher in the future, similar to that of Venus (>0.75) when the solar insolation rises and the Earth enters a runaway greenhouse condition (e.g., Li et al, 2009, doi:https://doi.org/10.1073/pnas.0809436106). But there are no observations and no known observational proxies of past top-of-atmosphere albedo outside of direct observations, and the albedo of those Earth system states or future ones that differ radically from the last several decades can only be modeled.

Because of the very practical and immediate impacts of albedo stability and symmetry in the coming decades, we encourage the community to focus on advancing the predictive understanding of albedo for those Earth system conditions that we expect in the 21st Century, which will likely not include ice ages or conditions like PETM or runaway greenhouse conditions. Furthermore, we would like the community to not necessarily feel the need to encompass those conditions which are unobserved, especially those that can only be tested by models. To be sure, the albedo for these conditions can be modeled, but it is also highly-underconstrained for the atmospheric and surface conditions that have occurred in the hundreds of thousands, millions, or even billions of years in the past or are likely to occur in the distant future of Earth. Again, it would be preferable if an albedo theory encompasses the full range of past, present and future Earth System states, but theory development for Earth system states that cannot be observed or will not be observable could create an over-reliance on Earth system modeling.

Language has been added to the non-requirement section to note that a theory that encompasses the broad range of future and paleo climates is preferred to the extent that it can be physics- and not modeling-based (revision 2, lines 110-114).

“To be sure, it is preferable for a theory to encompass Earth System states outside of the 21st Century, but there are no observations and no known observational proxies of past (or future) top-of-atmosphere albedo outside of direct observations. The albedo of those Earth system states that differ radically from the last several decades can only be modeled, so the applicability of a theory across these other states can only be developed and tested by models.”

4. l.118-133: Actually I find it surprising and interesting that the CMIP6 mean matches the observations so closely (Fig 3a). This seems like an interesting result that would merit more discussion. In particular, it the model-mean seems to capture the overall negative trend over the observational period, and the projections show that this trend simply continues to the end of the 21C. So the models suggest that global-mean albedo is not actually stable, but decreases in response to anthropogenic forcing? In fact, this decrease is consistent with the sea ice and snow retreat, increased SW absorption due to increasing water vapor, and negative SW cloud feedbacks that we generally expect in a

warming climate. So how do the model results stack up against your "unexplained phenomena" and the hypotheses presented later on? I suggest expanding discussion of these aspects and working them in to the discussion of your hypotheses later on.

We concur with Reviewer #3 that the performance of the multi-model mean of albedo with respect to observations is indeed noteworthy and merits discussion in the revised manuscript. At the same time, the underlying reasons for this performance are well beyond the scope of this current paper, as determining the root cause(s) of this emergent behavior of the multi-model mean requires, at the very least, variational tests of a range of climate model outputs. Nevertheless, the community-wide skill in modeling globally-averaged albedo and hemispheric symmetry clearly exceeds that of the skill of a single model and/or small group of researchers. The range of assumptions in Earth system parameterizations and choices for model tuning is broad but the emergent result is an unbiased estimate with respect to observations. This finding suggests that the Central Limit Theorem may be applicable for analyzing models and gaining insights into the observed albedo phenomena and that it may be appropriate to assume that each model is an independent, if imperfect, sample of a distribution of albedo. We have added text to the revised manuscript as follows (revision 2, lines 154-156).

“These findings suggest that the Central Limit Theorem may be applicable for analyzing multiple models, performing experiments with the ensemble, and gaining insights into the observed albedo phenomena because it may be appropriate to assume that each model is an independent, if imperfect, sample of possible globally- or hemispherically-averaged albedo.”

We have also added language to the abstract and throughout the main text of the revised manuscript highlighting this finding and pointing to the potential to use a multi-model ensemble for theory development.

Regarding Reviewer #3’s points on the apparent negative trend in globally-averaged albedo, we would contend that the trends in albedo and in the multi-model mean are apparently both negative. Again, though, the determination of the cause(s) of such apparent trends is outside of the scope of this manuscript because it also requires a variational analysis, along with extensive radiative transfer calculations and process model simulations to isolate the partial contributions of anthropogenic forcing, changes in cryospheric extent, water vapor, negative SW cloud feedbacks, and dynamics etc. This would follow Loeb et al, 2025, doi:https://doi.org/10.1073/pnas.2511595122), who performed a partial radiative perturbations and identified, in descending order of importance, clouds, surface albedo, and water vapor as causes of an emerging asymmetry.

We have added text to the Discussion section of the revised manuscript as follows to highlight the need to explore the use of multi-model means and research approaches that determine why multi-model mean skill relative to observations is higher than a single model for averages and trends (revision 2, lines 342-347).

“Finally, climate models, when they structurally support a working theory for albedo may be useful in predicting albedo and probing the limits of albedo stability and symmetry. The skill of the multi-model ensemble in reproducing observed albedo phenomena and emerging (though not definitive) trends suggests that variational analysis (e.g. in [23]) and experiments with the ensemble are warranted and may be supportive of theory development.”

*5. l.146-162: I have a hard time understanding what you mean by "intrinsic properties". The sentence "Earth’s top-of-troposphere planetary albedo and its hemispheric symmetry are the result of properties of the Earth system at the surface and in the troposphere that are invariant on the time-scales of the 21st Century" makes some sense --- I guess you are saying that albedo is *not* strongly affected by things that will change over the 21C, like CO2 and aerosol loading. But CO2 and aerosols affect clouds and water vapor, which are known to affect albedo. In fact it is very hard to think of anything in the climate system that will stay strictly invariant in the face of 21C radiative forcing. One thing we can be sure won't change is the layout of continents, but this is highly asymmetric across hemispheres and can't be used as a basis for explaining the hemispheric symmetry of albedo. So I am very confused about the precise content of your hypothesis. For example, do you mean to say that albedo itself is an intrinsically invariant property of the climate system, which does not change however much one changes CO2 or aerosols? Moreover, what exactly is the physical content of your hypothesis? For example, what possible mechanisms make Bond albedo be insensitive to surface albedo, as you claim in at l. 170?*

We acknowledge that “intrinsic properties” is not the most intuitive wording to represent what we mean here. To improve clarity, we have decided to rename this as the “invariant Earth system properties” hypothesis.

This hypothesis is that invariant properties of the Earth System are clearly central to albedo, so those properties could result in an integrated system where the albedo is highly constrained within a limited range on interannual time-scales.

This can best be considered from the perspective of performing all of the radiative transfer calculations necessary to produce globally- or hemispherically-averaged albedo values over the time-scales at which we observe albedo to be approximately constant. While the atmospheric and surface states will vary as inputs to those calculations, there will also be a large number of quantities in that calculation that are fixed. However, those atmospheric and surface states vary together according to atmospheric dynamic and thermodynamic principles. Reviewer #3 rightly points out that changing atmospheric and surface composition from rising CO2 to changes in aerosols, clouds, or the extent of frozen surface cover would be expected to impact albedo. But it is not necessarily the case that the atmospheric and surface state changes can, together, produce globally- or hemispherically-averaged changes in albedo because those states do not exist in isolation. This hypothesis is, therefore, that the constants that impact albedo are more determinative of albedo than the variability in atmospheric and surface states on interannual time-scales.

Language has been added to the revised manuscript as follows (revision 2, lines 166-167):

“More specifically, the small range of albedo on interannual time-scales may be constrained by a set of physical constants far more than the range of possible spatial and temporal variations of the Earth's atmospheric and surface states.”

In terms of what those constants are, there are several key examples: the spectroscopic absorption of water molecules and the refractive indices of frozen and liquid water greatly impact globally-averaged or hemispherically-averaged albedo by establishing shortwave atmospheric absorption and the reflectance/absorption of clouds and frozen surfaces. In the previous manuscript, we provided a bulleted list of examples of invariant properties that could fall into this category to assist the reader’s understanding. To improve clarity, we have now moved the bulleted list of examples immediately after the reframed hypothesis is introduced (revision 2, lines 170-175).

At the same time, we want to be clear that we are merely putting forward the invariant Earth System properties hypothesis to show that it can be tested in our framework. We are not claiming that this hypothesis is true, or advocating for its acceptance within the community. We are merely presenting it as an alternative to the cloud buffering hypothesis to show that there are distinct observational corollaries to both hypotheses.

As outlined in the revised manuscript (revision 2, lines 182-191), there would be a number of observational corollaries, if this hypothesis were true. One observational corollary is that the albedo response to two or more identical perturbation events like a stratovolcanic eruptions should be identical. We recognize that no two eruptions perturbations (e.g., eruptions) are identical, but a similar albedo response to similar eruptions should occur to confirm this hypothesis. Ultimately, when confronted with the observations available to date, or continued observations in the 21st Century, one can follow the falsification framework outlined in Figure 2 to test the hypothesis.

*6. l.178-193: Cloud buffering hypothesis. This hypothesis has more physical content, in that it points to a specific mechanism by which the climate system may arrange itself to maintain roughly constant albedo. In this sense, it seems like a more precise and specific version of your previous hypothesis (but I am unsure about this since I don't understand your "intrinsic" hypothesis). In any case, isn't the hypothesis immediately falsified by the observation that Bond albedo increases a lot during Pinatubo? I note that you cite Gristey and Feingold 2025 in support of your hypothesis (l.185). But if I understand that paper correctly, it actually says the opposite: it says that cloud scattering (and therefore cloud albedo, and thus SW CRE) actually *increases* in the presence of stratospheric aerosol, instead of decreasing as your hypothesis requires (I quote from their abstract: "diffusion of the radiation field that would accompany stratospheric aerosol injection can result in sunlight entering clouds at steeper*

angles, which increases cloud albedo without actively injecting aerosols into clouds.")

The reviewer is correct that Gristey and Feingold 2025 generally show an increase in cloud albedo when stratospheric aerosols are present. In the current study, we do not intend to claim that this effect is causing compensation in albedo. This is an instantaneous effect, so it is separate from any potential compensating cloud radiative effects that would act as part of the cloud buffering hypothesis. The purpose is to point out that non-trivial interactions between stratospheric aerosols and clouds exist and could be important in this context. To avoid confusion, we have edited this sentence to say (revision 2, lines 199-201) "Any compensating cloud radiative effects would also need to account for the fact that stratospheric aerosols may also immediately brighten clouds via diffusion of the radiation field[54]"

7. As discussed in 5 and 6, the hypotheses as they stand seem hazy and underdetermined to me. I suggest sharpening them up and possibly dropping the "intrinsic" hypothesis. The cloud buffering hypothesis on the other hand sounds interesting and could be a good case for testing with you ML approach.

We concur on the need to sharpen the hypotheses.

The idea is that globally- or hemispherically-averaged albedo, as a function of radiative processes in the surface and atmosphere that materially impact the flows of shortwave radiation and ultimately its reflection back to space, is simply a constant that flows down from other constants, and while atmospheric and cryospheric dynamics can temporarily perturb this quantity, the fundamentals of the Earth System (such as the Earth's orbital parameters, solar insolation, the fraction of its area that is covered by land, the gaseous and condensate composition of the atmosphere, the optical properties of gases and condensates, and thermodynamics) ultimately restore albedo to a constant value on multi-year time-scales. It is this idea that constant properties of the Earth System are restorative for albedo. This has observational implications: the time-varying response of the Earth System to identical perturbations when that Earth System is in an identical state should also be identical.

We have modified the language in the section of the manuscript beginning on line 162 to reflect this sharpening. Ultimately, we still feel it is important to have a contrasting hypothesis to the cloud buffering hypothesis, so that (1) the reader is left with the impression that there are competing explanations for albedo phenomena, and (2) that the response of the Earth System to future stratovolcanic eruptions will be able to falsify at least one of these hypotheses.

8 l.194-221: ML methods. ML is relatively new to climate science, and needs to be introduced with some care and attention that the reader understands what is being done and how results should be interpreted. The material under the methods section unfortunately reads to my untrained eye like a jargon word salad. Please rewrite in more user-friendly language (where "user" in this case means "climate scientist with very little previous contact with ML", which is the majority of the community at the current

*stage), and provide some intuitive insight into how the methods work and how they are different from standard regression. In particular you are using SHAP, which if I understand correctly is an attempt to make ML interpretable; if so, please spend a bit more time explaining *how* it should be interpreted.*

We appreciate the Reviewer's comment about the need for a careful and attentive introduction to the methods and interpretation of results. Several of the authors of this manuscript have climate science backgrounds and had little previous contact with ML prior to working on the material presented here, while other authors are well-versed in the methods and interpretations, and both groups have learned much from each other and need to express that in the manuscript.

The revised manuscript has been rewritten to provide a smooth on-ramp for climate scientists with little previous contact with ML. We recognize that broaching of the FFNN, SHAP, and the U-Net in the manuscript was jarring and without context. We have therefore added more explanatory material to describe conceptually what the methods are doing in service of a deeper understanding about the relationships between predictor variables and the predictand, all while minimizing jargon.

The essence of SHAP, in particular, is important for this paper. The method answers the question of why a particular value of albedo occurs as a function of input variables. SHAP has its origins in game theory for determining the partial, potentially nonlinear contributions of many factors to an outcome, weighted by how many times each marginal contribution of that parameter arises.

The revised manuscript now reads (revision 2, lines 228-235):

"The feature importance analysis is performed with Shapley Additive Parameter (SHAP) analysis (See Methods for details) which produces a ranked set of contributions of each input atmospheric or surface state feature to TOA albedo values across space and time (Shapley, 1953; van Straaten, 2022; Flora et al, 2022a,b). SHAP works by estimating each physical variable's marginal contribution to the model's predictions, regardless of the linearity of the model. SHAP therefore produces an interpretable measure of the impact of a set of atmospheric and surface state characteristics on albedo. This approach answers the fundamental question of why a model produces an output given a set of inputs when the inputs contribute differentially to the outcome. Because SHAP is a model-agnostic framework, it allows us to focus first on achieving sufficient predictive skill with the FFNN and then apply SHAP as an interpretability layer to diagnose which physical parameters the model relies on most strongly in producing those predictions."

9. Fig 4. It's interesting that linear regression indicates a weak role for latitude, while ML indicates a leading role. Climatologically, albedo has a big meridional temperature gradient (as shown in Fig 1), so I would expect even linear regression to pick that up. Providing more insight into reasons for the difference between these two methods would give a better sense for the value added by the ML approach.

We concur with Reviewer #3 that there are clearly meridionally-varying processes that impact albedo, and it is encouraging that both linear regression and ML methods pick this up, but, as

we and the Reviewer note, ML methods determine that the importance of these processes is more than linear regression does. So while linear regression predicts that the latitude of a location is a predictor variable for a location's albedo, the method finds that other predictor variables are more important.

The primary reason for the different results from the two methods is that those processes that produce meridional gradients in albedo are nonlinear, both in terms of the radiative transfer for a given atmospheric and surface state that reflects shortwave radiation and the dynamics that produce that atmospheric and surface state to begin with. For the latter effects, frozen surface coverage, mid-latitude subsidence, Hadley and Farrell cell circulation, and ocean heat transport, to name a few, are the result of nonlinear processes. Advanced data methods like ML have the ability to capture nonlinearities in their data analysis while linear regression does not.

This above paragraph, and the following figures and analysis, which we have added a figure to the Supplemental section that shows this.

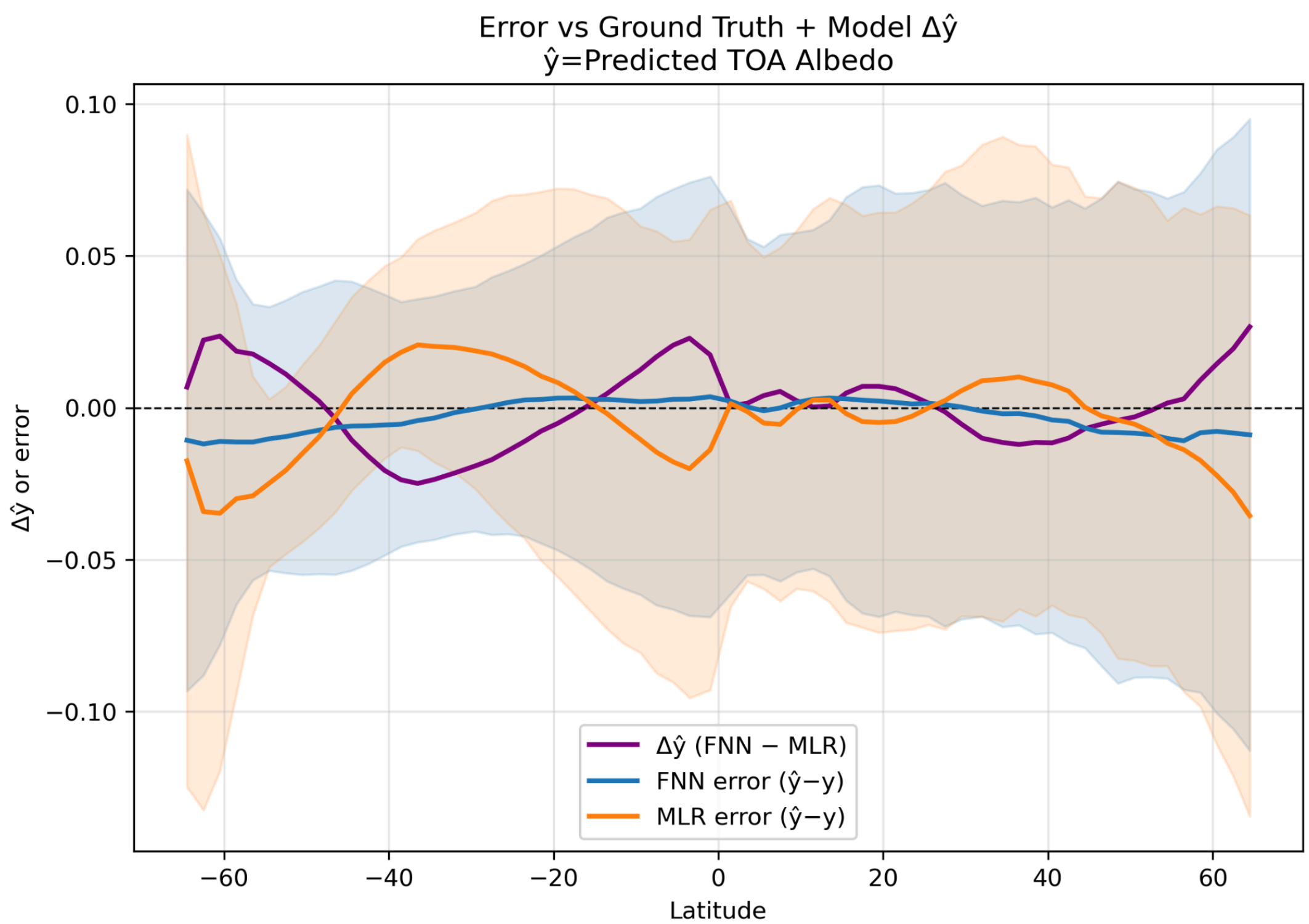

Here we plot the out-of-sample errors in prediction for the Fourier Neural Network (FNN) and Multi-Linear Regression (MLR) models, as well as the difference between the model predictions. We see the MLR errors are both larger and vary more meridionally than the FNN errors. The FNN model outperforms the MLR model because TOA Albedo is non-linearly dependent upon latitude. The FNN model has more degrees of freedom than the MLR and the relative performances results of the two models indicate that more degrees of freedom are needed for this problem than MLR can provide.

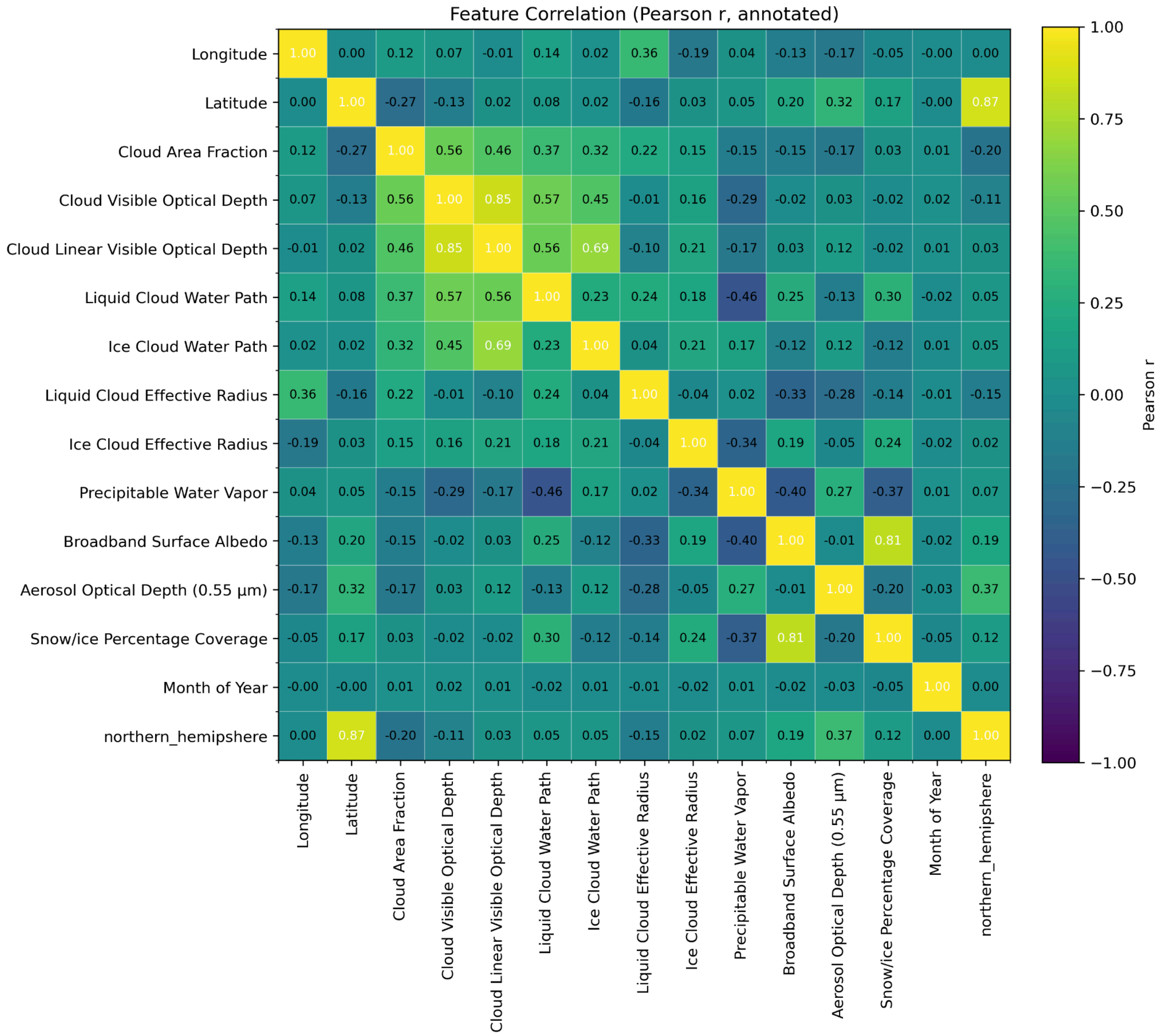

Finally, the correlation matrix of predictors for top-of-atmosphere albedo indicates many off-diagonal components of varying signs and magnitudes, showing the complex and non-linear dependencies of TOA Albedo on latitude.

10. It doesn't seem to me that your ML application actually tests the hypotheses you propose. Why not do a more direct test --- for example, you could look at correlations between surface albedo and cloud albedo to directly test you cloud buffering hypothesis, or put another way, check if your ML method predicts decreased cloud albedo when surface albedo increases. Along the way it might turn out that simple linear regression does not capture this correlation---if so, that would go a long way to buttressing your case that ML have a useful and unique role to play in understanding Earth's albedo.

We appreciate the need for more clarity about what the ML methods are achieving with respect to testing our hypotheses. To Reviewer #3's recommendation, we have performed a detailed analysis of albedo and albedo predictors to determine the relationships between cloud area fraction and broadband surface albedo.

To our surprise, Reviewer #3's suggestion led to analysis that does not reject the Cloud Buffering Hypothesis. The imputation analysis we performed provides some measure of support for that hypothesis, but it is by no means definitive. Regardless, that hypothesis remains viable and worth exploring further, and we have modified this second revision of the manuscript so that, rather than evaluating the Invariant Earth System Properties Hypothesis, we evaluate the Cloud Buffering Hypothesis and discuss its potential merits.

The below figure shows the 2-D histogram of the raw relationship and reveals that there are several distinct patterns for different surface types. We can see that for ocean surfaces (lowest horizontal bright line), cloud area fraction is high while more complex patterns exist over other parts of the 2-D histogram.

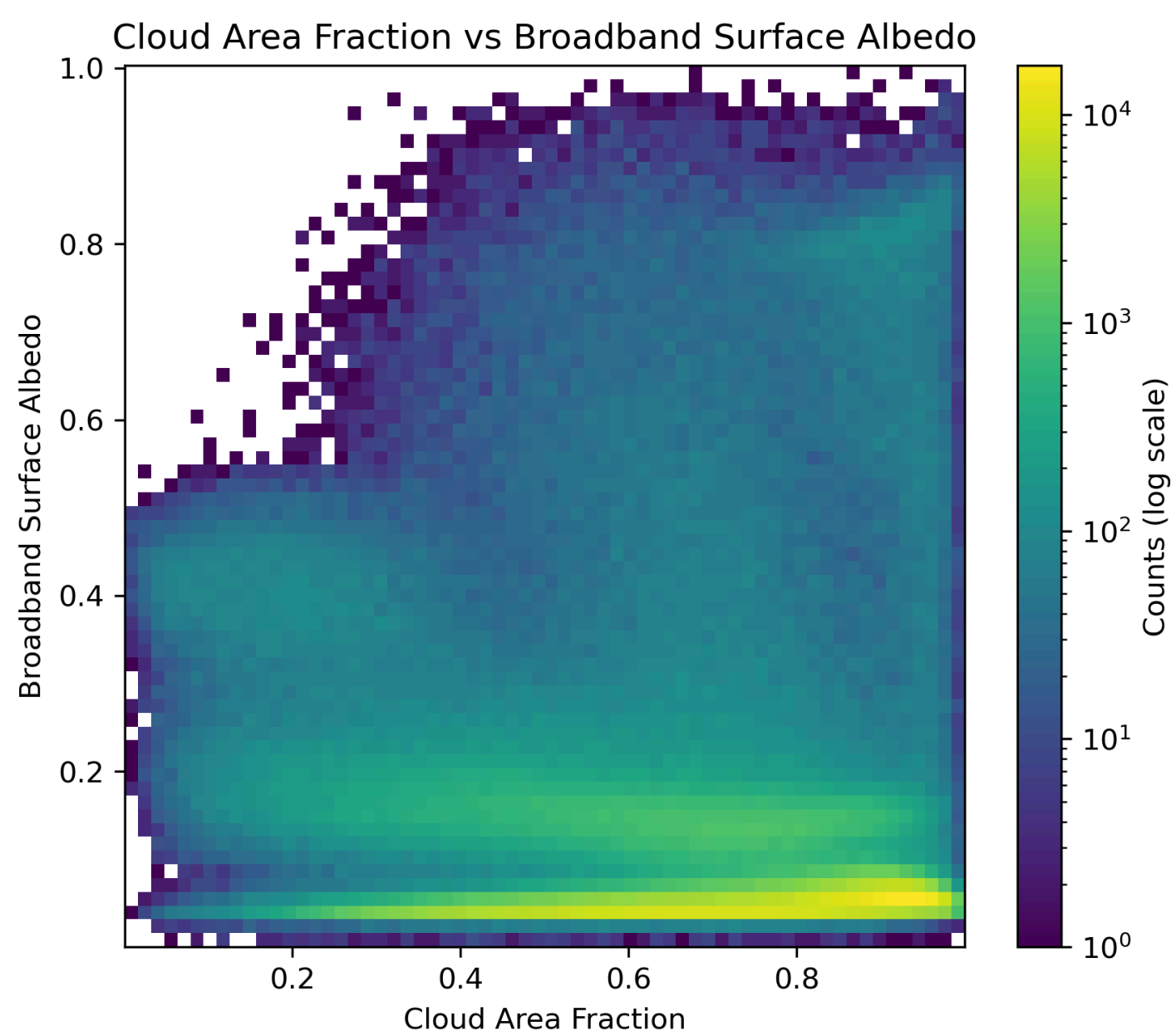

The FFNN trained on these data can then be used to determine if an imputed change in surface albedo would suggest a positive or negative change in Cloud Area Fraction (CAF), or, alternatively, no change. As shown below, we find that CAF responds inversely, monotonically, and non-linearly to fractional changes in broadband surface albedo (e.g., δ).

The left panel shows CAF vs surface albedo where the dark blue line indicates the observed relationship, while the other lines indicate the CAF that the FFNN predicts when surface albedo is changed. Meanwhile, the right panel isolates these effects by showing the change in CAF vs surface albedo. Where surface albedo is reduced (δ<1), the FFNN predicts increased CAF and vice versa.

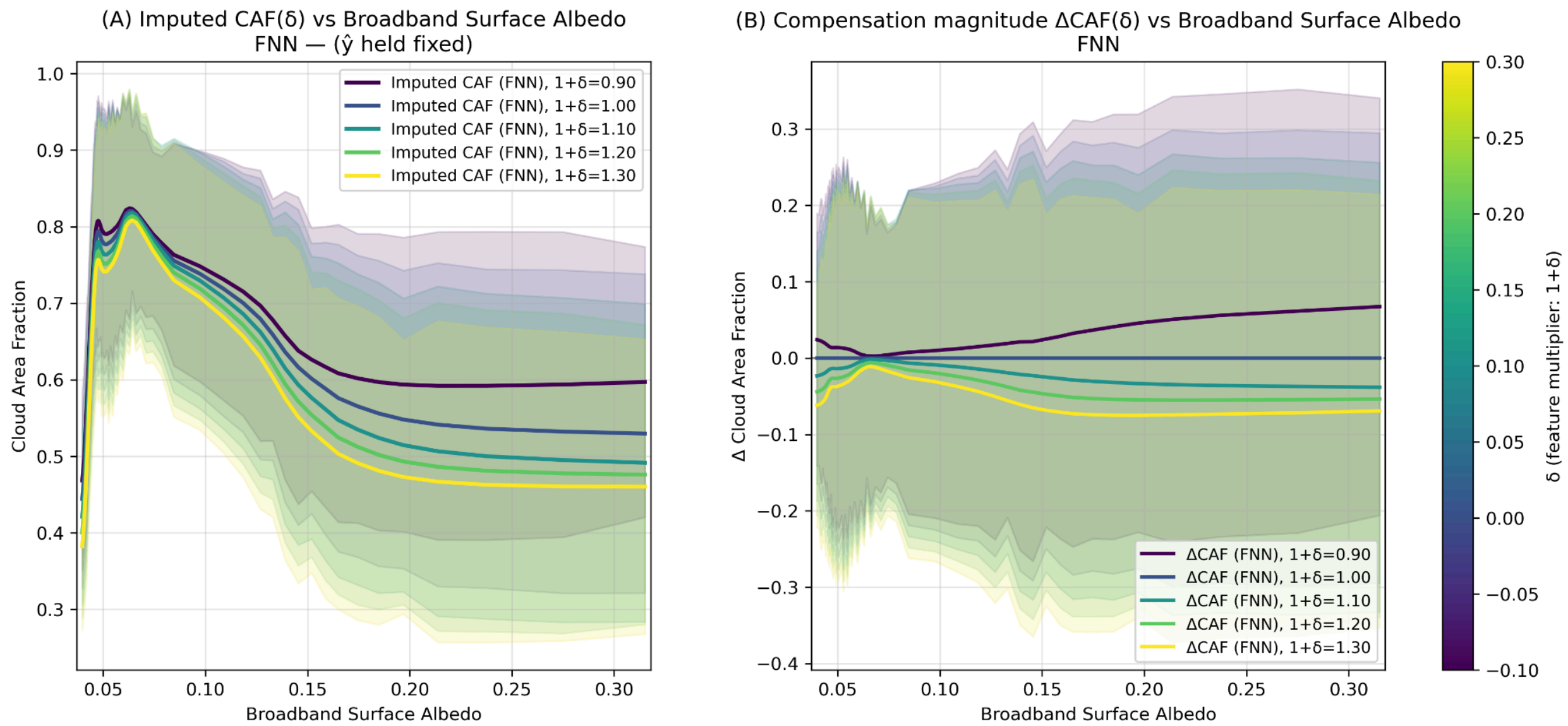

We note that the actual system complexity is quite high and so it is not physical to vary surface albedo in isolation (and absent realistic covariances) from atmospheric circulation and other predictor variables, but in aggregate, the inverse relationship between CAF and surface albedo exists, and, furthermore, is best-evaluated with nonlinear data analysis methods, since linear methods will belie the complex relationships between CAF and surface albedo that the data reveal.

We find from these plots that the data do not contradict the cloud buffering hypothesis. This is not to say that the cloud buffering hypothesis is supported, but rather, advanced data analytic techniques do not reject it as a hypothesis.

We have added this latter figure to the SI Appendix (Supplemental Figure 2) and included text (lines 24-32) to describe these results in the revised manuscript.

Dear Editor,

We thank the three Reviewers for their time and effort to review our manuscript “Towards a Theory for Earth’s Albedo Stability and Hemispheric Symmetry in the 21st Century” for *Communications Earth and Environment* so thoroughly and similarly thank your office for your time and effort here. We provide a point-by-point response (in plain text) to each comment (in blue italics) as well as a revised manuscript that specifically addresses all of the referees' concerns.

Reviewer #3 (Remarks to the Author):

The authors have responded very thoroughly to my previous comments and I find the paper much improved. In particular the new results regarding the cloud buffering hypothesis are very interesting. The general presentation and framing of the hypotheses is also a lot clearer now. I can recommend the paper for acceptance in its current form.

We thank Reviewer #3 for a thorough review and appreciate the recommendation for acceptance of the paper.
