# Supplementary material for: Towards a theory for Earth’s albedo stability and hemispheric symmetry in the 21st Century
Source: Commun Earth Environ. 2026 Jul 21;7(1):884. doi: 10.1038/s43247-026-03817-0 (PMC13751292; doi:10.1038/s43247-026-03817-0)
Supplement: Supplementary file 2 [file 43247_2026_3817_MOESM2_ESM.pdf]

Supplementary Information: Towards a Theory for Earth's Albedo Stability and Hemispheric Symmetry in the 21st Century

Daniel R. Feldman[1,*], Jake J. Gristey[2,3,4], Maria Z. Hakuba[5], Doug Hellinger[6], and Samuel Kahn[7]

[1]Earth and Environmental Sciences Area, Lawrence Berkeley National Laboratory, Berkeley, CA USA
[2]Cooperative Institute for Research in Environmental Sciences, University of Colorado, Boulder, CO, USA
[3]NOAA Chemical Sciences Laboratory, Boulder, CO, USA
[4]Laboratory for Atmospheric and Space Physics, University of Colorado, Boulder, CO, USA
[5]Jet Propulsion Laboratory, California Institute of Technology, Pasadena, USA
[6]Santa Cruz Institute for Particle Physics, University of California-Santa Cruz, Santa Cruz, CA, 95064,USA
[7]Research Fellow, University of California-Santa Cruz, Santa Cruz, CA, USA
[*]Corresponding Author: drfeldman@lbl.gov

1 Supplementary Notes 1

1.1 Feed-Forward Neural Network Performance over Time

The bias of this Feed-Forward Neural Network (FFNN) described in the Methods is evaluated over time in Figure 1. Here, the model is trained with CERES EBAF predictors and albedo values from 2000 through 2018, and then we track the bias and RMSE of the model's predictions of EBAF albedo from 2019 onwards. We see that the model's predictive skill degrades over time. It starts out at less than +0.0005 (in reflectance units) with and RMSE of less than 0.001 (also in reflectance units) better than 0.001 in predicting CERES albedo over 2-years with all CERES EBAF predictors for those 2 years withheld. The model's bias increases to -0.002 relative to EBAF with an RMSE of 0.004 after 4 years where all EBAF training data is withheld for 4 years.

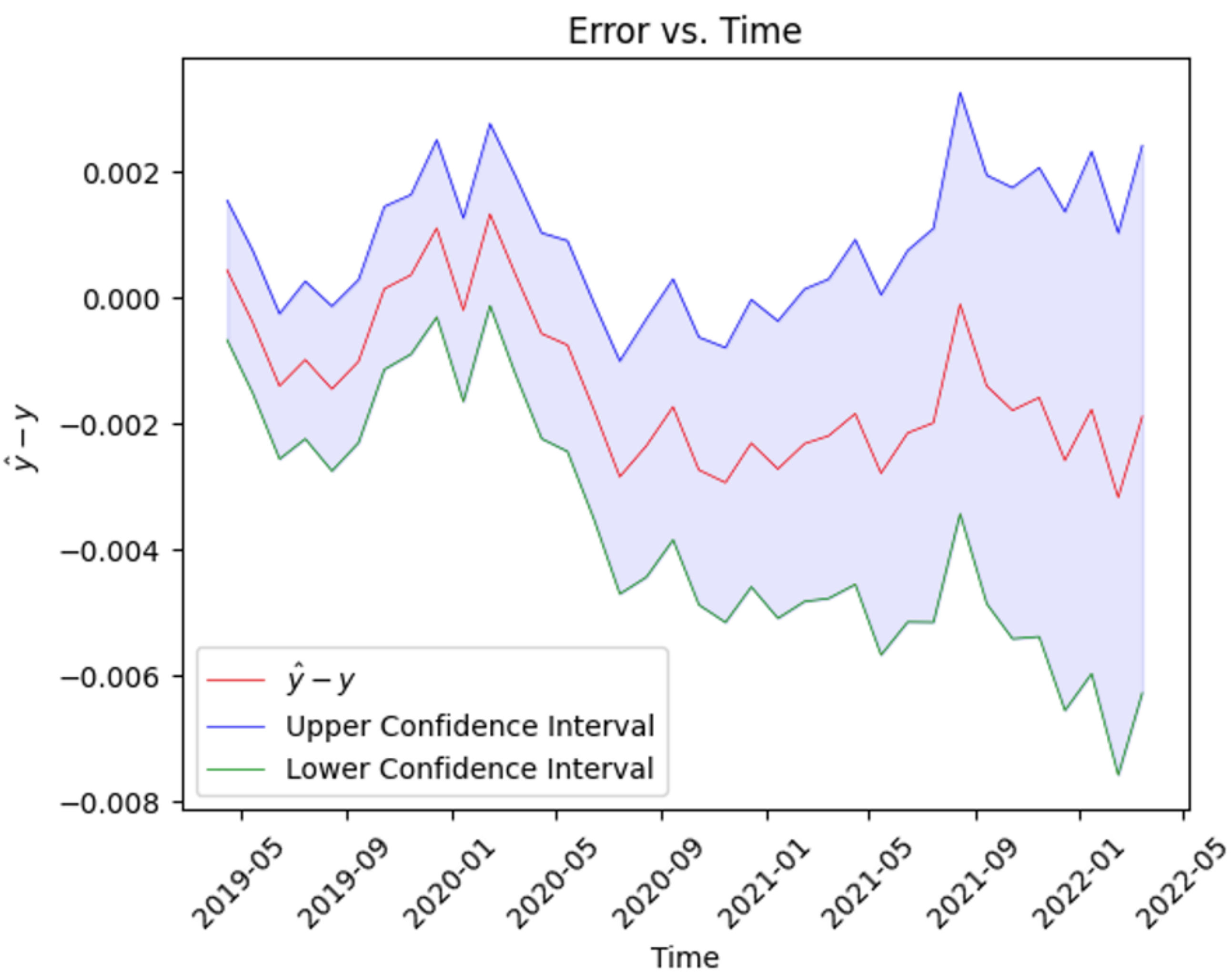

Supplemental Figure 1. FFNN skill degradation over time when trained on data from 2000 through 2018 and with bias and RMSE relative to CERES EBAF.

2 Supplementary Notes 2

2.1 FFNN Cloud Buffering Hypothesis Test

In order to test Cloud Buffering Hypothesis, we can utilize the learned relationships between cloud area fraction (CAF) and surface albedo in the FFNN in order to determine if an imputed change in surface albedo would suggest a positive or negative change in Cloud Area Fraction (CAF), or, alternatively, no change. As shown in Figure 2, we find that CAF responds inversely, monotonically, and non-linearly to fractional changes in broadband surface albedo (e.g., δ).

The left panel shows CAF vs surface albedo where the dark blue line indicates the observed relationship, while the other lines indicate the CAF that the FFNN predicts when surface albedo is changed. Meanwhile, the right panel isolates these effects by showing the change in CAF vs surface albedo. Where surface albedo is reduced ($\delta < 1$), the FFNN predicts increased CAF and vice versa.

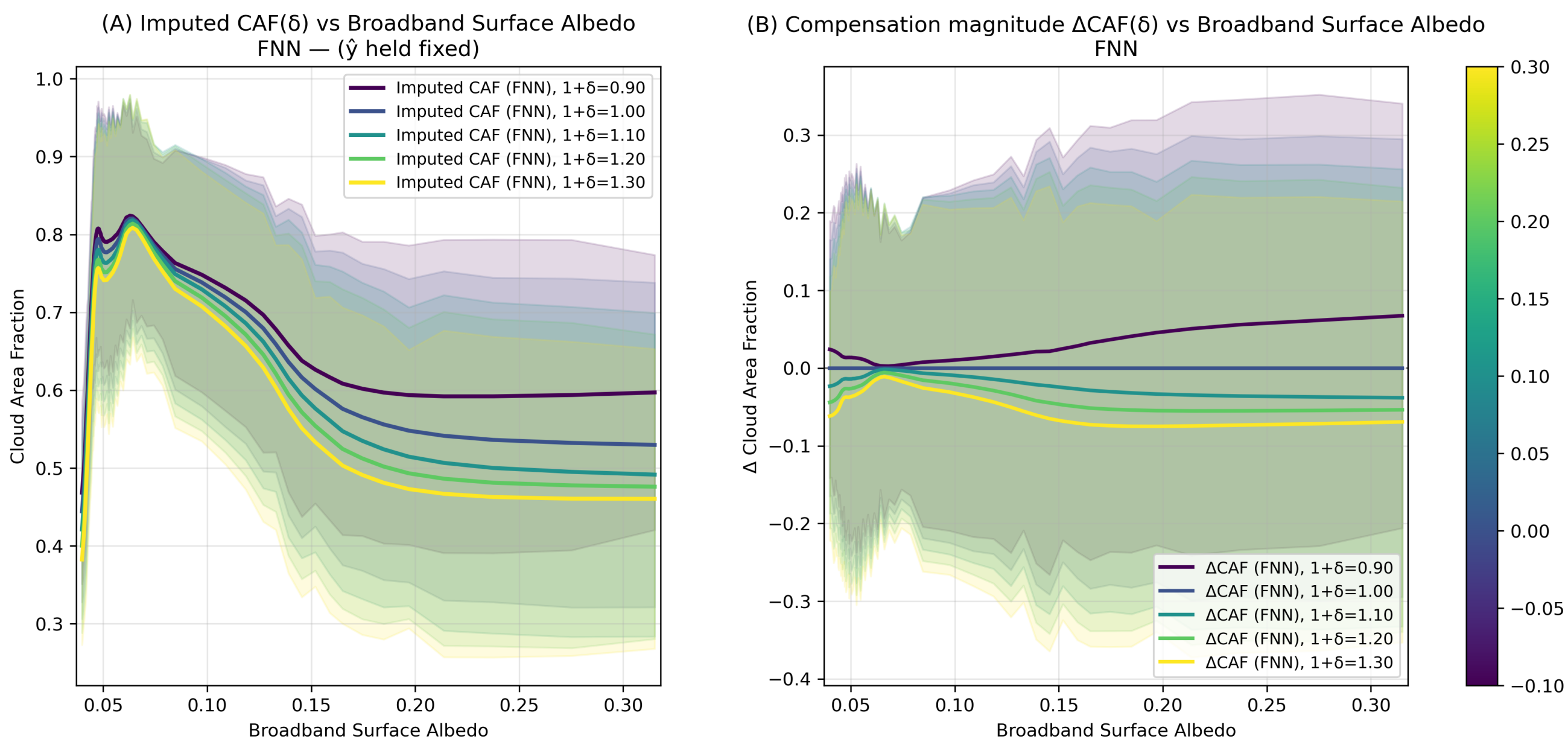

Supplemental Figure 2. (Left) Learned relationship between cloud area fraction (CAF) and surface albedo in CERES EBAF and imputed relationship between CAF and surface albedo where surface albedo increases or decreases by δ. (Right) Same as left panel but just showing change in CAF.

3 Supplementary Notes 3

3.1 U-Net Ablation Study Description

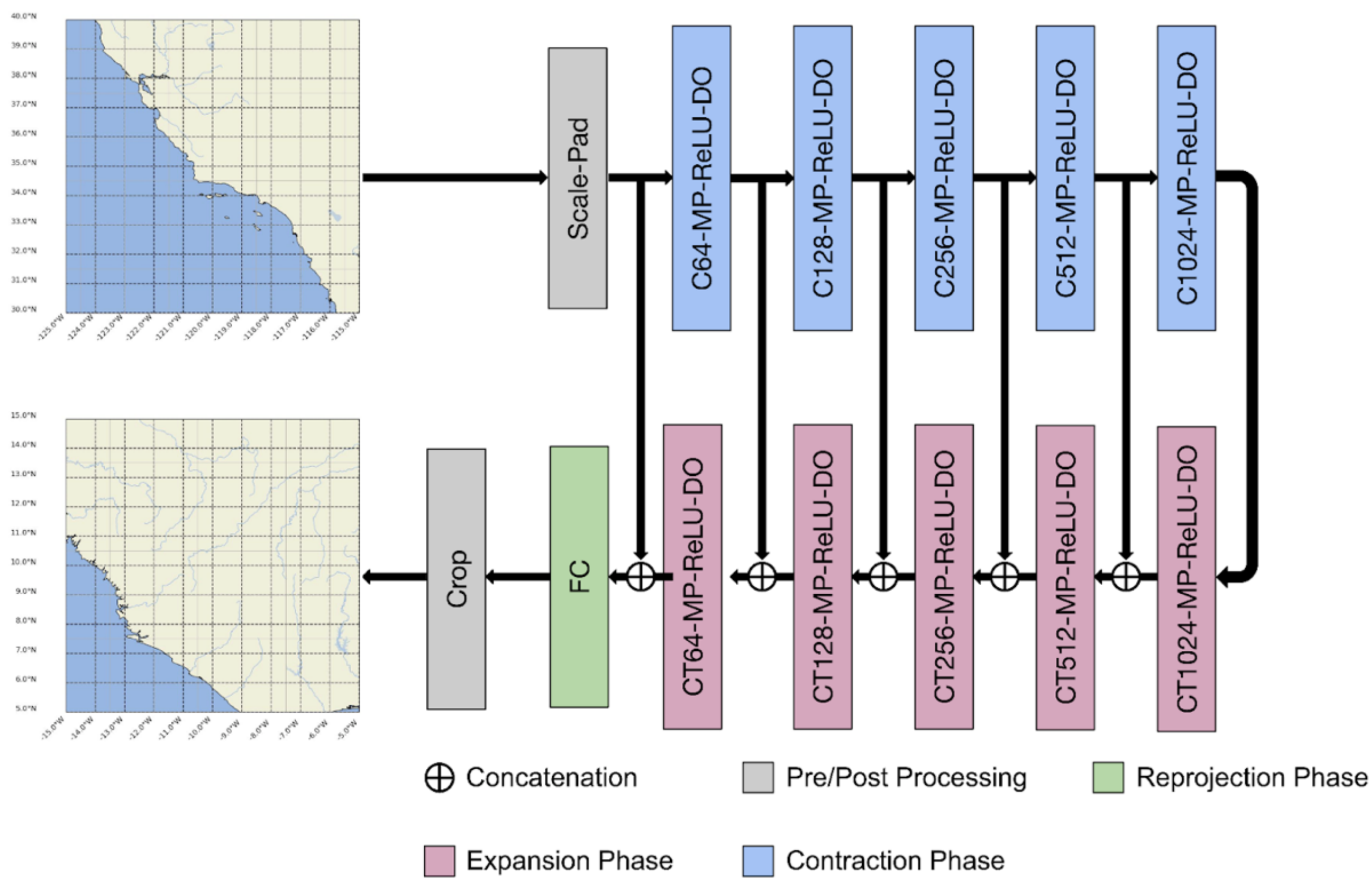

Supplemental Figure 3. Albedo prediction U-net architecture showing processing steps of the U-Net to capture and predict spatial and temporal features. The U-Net training performs a series of data-driven spatial and temporal feature compression (contraction through a Rectified Linear Unit and Dropout ReLU-DO), as shown in blue, to describe the relationships between albedo predictors and albedo with a fixed number (64, 128, 256, 512, 1024 depending on the U-Net level) of data-derived features. Then, the U-Net expands (as shown in purple) and seeks to reproduce in-sample albedo values with that number of features from depending on the U-Net level. Finally, the U-Net training includes full-connection (FC, in green) post-processing to develop an optimal number of spatio-temporal features. Once trained, the U-Net predicts training-withheld albedo values based on training-withheld albedo predictors and the training-derived spatio-temporal features.

In order to ensure that the U-Net (with architecture depicted in Supplementary Figure 3) is realistic for capturing temporal and spatial correlations in TOA albedo (and is not over-trained or developing spurious correlations), we perform an ablation study to examine the change in network performance when predictor variables are removed or shuffled or artificial noise is added to those variables. This U-Net extreme parameter analysis produces changes in the U-Net albedo prediction RMSE with respect to its ability to predict CERES EBAF Ed4.1 albedo in the absence of such ablation. Specifically, the network performance is examined for predicting the time-series of planetary albedo from the time-series of a single grid point (and then each single grid point is evaluated). Here, we plot the network's responses in accuracy to extremely unphysical feature replacements (median-centered 6σ gaussian noise). The network is quite robust to non-spatiotemporal features (Supplementary Figure 4: left plot) and qualitatively exhibits the extreme errors one might expect from physically impossible spatial coordinate perturbations (Supplementary Figure 4: right plot). Random target spatial perturbations directly decouple the output with the predictors, and random grid points for the input features decouple the selected CERES input features from their geographic contexts. Both result in immense distortions in predictions with distortion of the target the most severe, as one would expect (since there is no way to infer a deliberately mislabeled target and the perturbed target coordinates will generally be unphysical, since they are not constrained to their normalized periodic values). Whereas spatial perturbations are at best informed by global

spatial means, temporal perturbations (right plot) can be informed by local spatiotemporal means, resulting in considerably less degradation in accuracy, despite extreme distortion of target date.

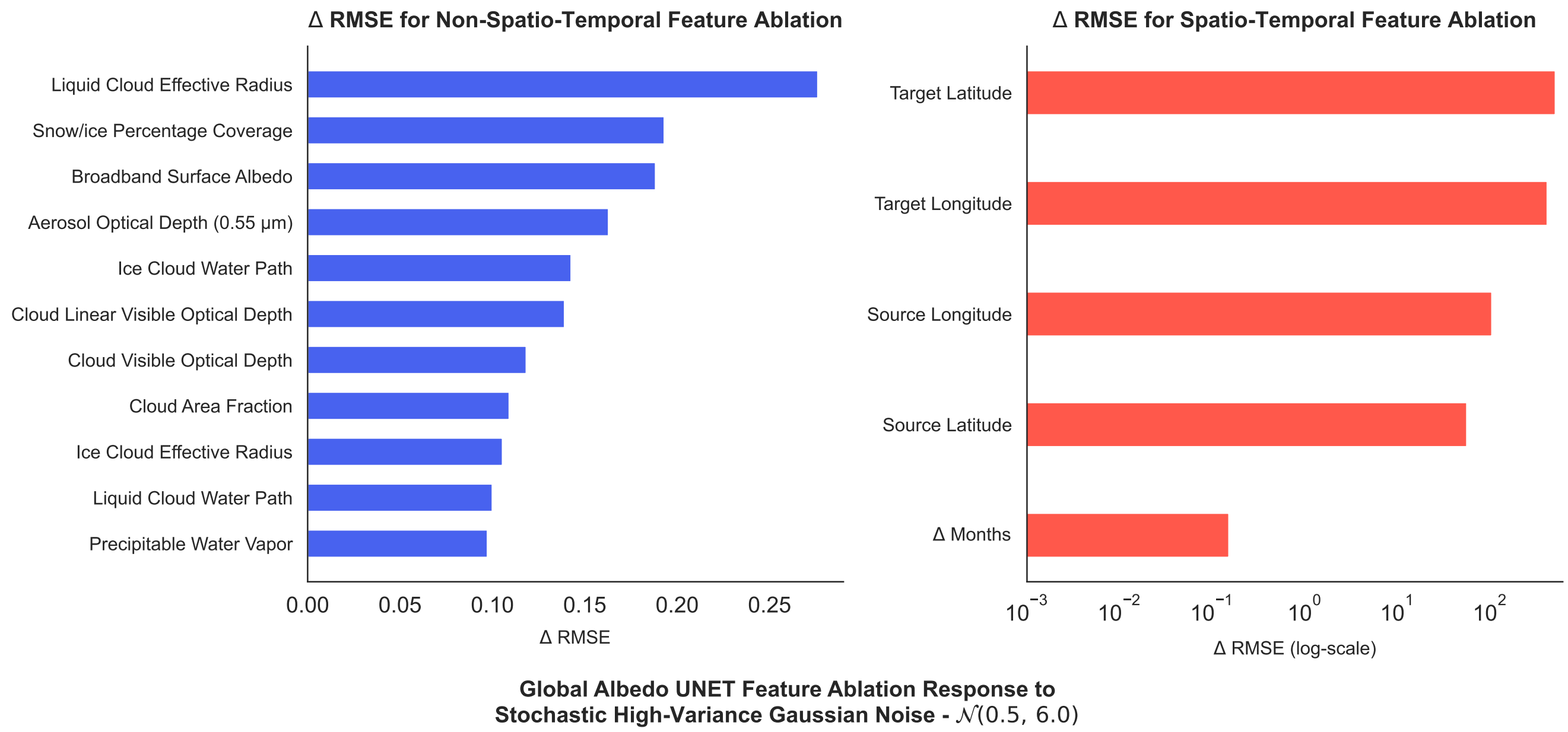

Supplemental Figure 4. U-Net ablation analysis results with change in U-Net albedo prediction RMSE relative to CERES EBAF Ed4.1 albedo for predicting planetary albedo from a single grid point.

4 Supplementary Notes 4

4.1 DEEP-C Albedo Calculation

We calculate planetary albedo with data from the Diagnosing Earth's Energy Pathways in the Climate System (DEEP-C), specifically the monthly- and globally-averaged Absorbed Shortwave Radiation (ASR) field that is part of Version 5.0 of that product[1,2] along with the corresponding monthly average of the daily Total Solar Irradiance (TSI) from the Community Consensus Composite of TSI developed by Greg Kopp (https://spot.colorado.edu/~koppg/TSI/) which followed an established methodology to create a TSI composite.[3].

Supplementary Figure 5 shows the DEEP-C albedo time-series from 1985-2020. This figure is similar to Figure 3a in the main text, except that the temporal range is different between the figures.

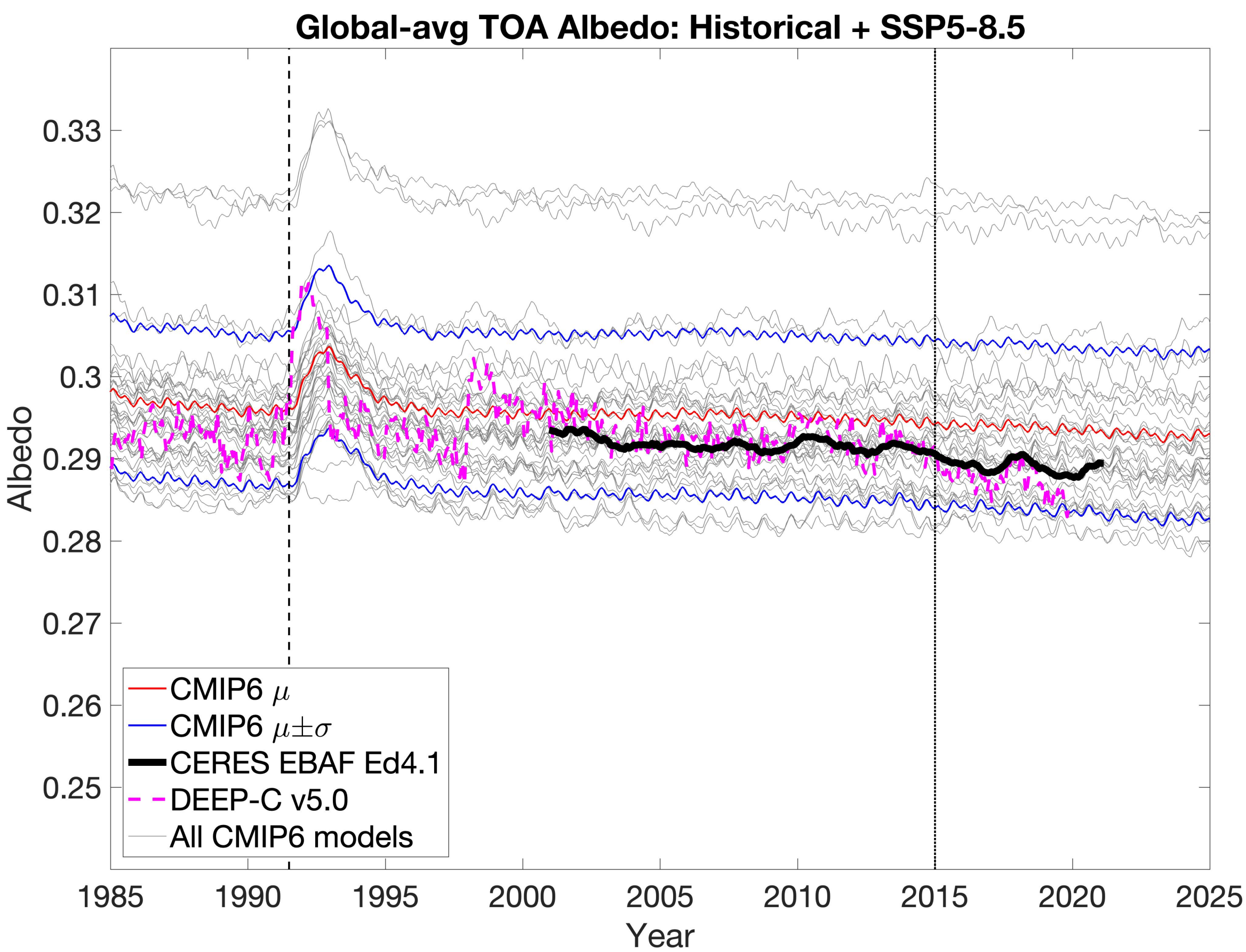

Supplemental Figure 5. Globally-averaged TOA albedo as measured by CERES-EBAF (black), DEEP-C and Kopp database (pink dashed) and historical simulations and future projections from a suite of CMIP6 models with the multi-model average and standard-deviation depicted. The vertical dashed black line indicates the Mt. Pinatubo stratovolcanic eruption in June, 1991. Vertical dotted line marks end of CMIP6 historical simulations.

5 Supplementary Notes 5

5.1 Data Model Complexity

In this paper, we utilize advanced, nonlinear methods in support of the development of a working theory for albedo, but recognize that linear and/or lower-complexity data models are more accessible and less opaque than advanced, nonlinear methods. Therefore, the use of a nonlinear, more opaque predictive model for albedo theory development requires justification.

There are strong nonlinearities in the integro-differential form of the Radiative Transfer Equation that governs what the albedo of Earth is for a given set of atmospheric and surface conditions. Furthermore, the atmospheric and surface components that we know impact albedo including atmospheric dynamics, cryospheric evolution, and vegetation dynamics, all contain strongly nonlinear interactions themselves. Consequently, we note that linear data models will necessarily be approximations that may not be appropriate across the range of conditions on Earth that contribute to the albedo field. There are also assumptions required for linear methods (multivariate normality, homoscedasticity), but whether or not violations of those assumptions preclude the use of linear is, we would argue, ultimately evidenced in the performance of those linear methods to generalize and predict albedo with predictors and predictands that are withheld and/or not included in the training.

At the same time, we recognize that there is a large range in complexity of data models: linear regression models have only a handful of parameters and that number of parameters is generally equal to the number of predictors unless features are engineered in such models. Meanwhile, advanced AI models may have millions of parameters in them. Because our understanding of the physical processes that produce the observed albedo phenomena is incomplete, we do not know a priori the optimal structure and complexity of a data model which can support albedo science.

To establish whether linear or nonlinear data models are more optimal for albedo science, we have performed an analysis to intercompare multi-linear methods to neural networks, since multi-linear regression (MLR), in particular, is straightforward to implement and explain, and may be sufficient for this problem. In Supplementary Figure 7, we perform a representative study comparing an MLR and a feed-forward neural network (FFNN) for predicting the CERES EBAF albedo field based on the set of predictor variables included along with the EBAF product. This figure clearly shows the reduction in the predictand (albedo) error when the more advanced FFNN data model is used.

Furthermore, Supplementary Figure 8 shows that there are key differences in research directions suggested by an MLR data model vs. the FFNN data model. This figure presents the contrast in feature importance results (mean SHAP values). In particular, the FFNN indicates that "latitude" is a variable that is far more important for the FFNN model's performance than for the MLR model's performance.

We explore this result further with additional error analysis in Supplementary Figure 6. This figure plots the out-of-sample errors in prediction for the Fourier Neural Network (FFNN) and Multi-Linear Regression (MLR) models, as well as the difference between the model predictions. We see the MLR errors are both larger and vary more meridionally than the FFNN errors. The FFNN model outperforms the MLR model because TOA Albedo is non-linearly dependent upon latitude. The FNN model slo has more degrees of freedom than the MLR and the relative performances results of the two models indicates that more degrees of freedom are needed for this problem than MLR can provide.

While both the MLR and FFNN point to the importance of cloud area fraction and surface albedo, the ordering of importance, particularly with respect to latitude and the contrast between the northern and southern hemisphere ("northern hemisphere flag") between the two importance identified by the two methods is striking.

Ultimately, the FFNN points researchers in directions of explanations for albedo phenomena that consider the following: meridional heat transport, the meridional variation in insolation and frozen surface extent, as well as other meridionally-varying features and differences between the northern and southern hemispheres that may be important, while also indicating that clouds and surface albedo are key predictors for TOA albedo. While the MLR model also points to clouds and surface albedo as key predictors, it does not point to latitude as a predictor, and rather points to the importance of precipitable water vapor for explaining albedo phenomena. Thus, the MLR model could be suggesting that clear-sky water vapor radiative effects are important for TOA albedo along with atmospheric humidity. The contrast in feature importance between the SHAP of the MLR and the FFNN indicates that these data models guide researchers in very different directions as they investigate the root causes of these TOA albedo phenomena.

The primary reason for the different results from the two methods is that those processes that produce meridional gradients in albedo are nonlinear, both in terms of the radiative transfer for a given atmospheric and surface state that reflects shortwave radiation and the dynamics that produce that atmospheric and surface state to begin with. For the latter effects, frozen surface coverage, mid-latitude subsidence, Hadley and Farrell cell circulation, and ocean heat transport, to name a few, are the result of nonlinear processes. Advanced data methods like ML have the ability to capture nonlinearities in their data analysis while linear regression does not.

Given this and the superior predictive performance of the FFNN, it is worth exploring meridionally-varying and hemispherically-contrasting processes for albedo phenomena, as opposed to ones that are focused on precipitable water vapor. The directions suggested from the FFNN are, therefore, more helpful than the guidance from the MLR model.

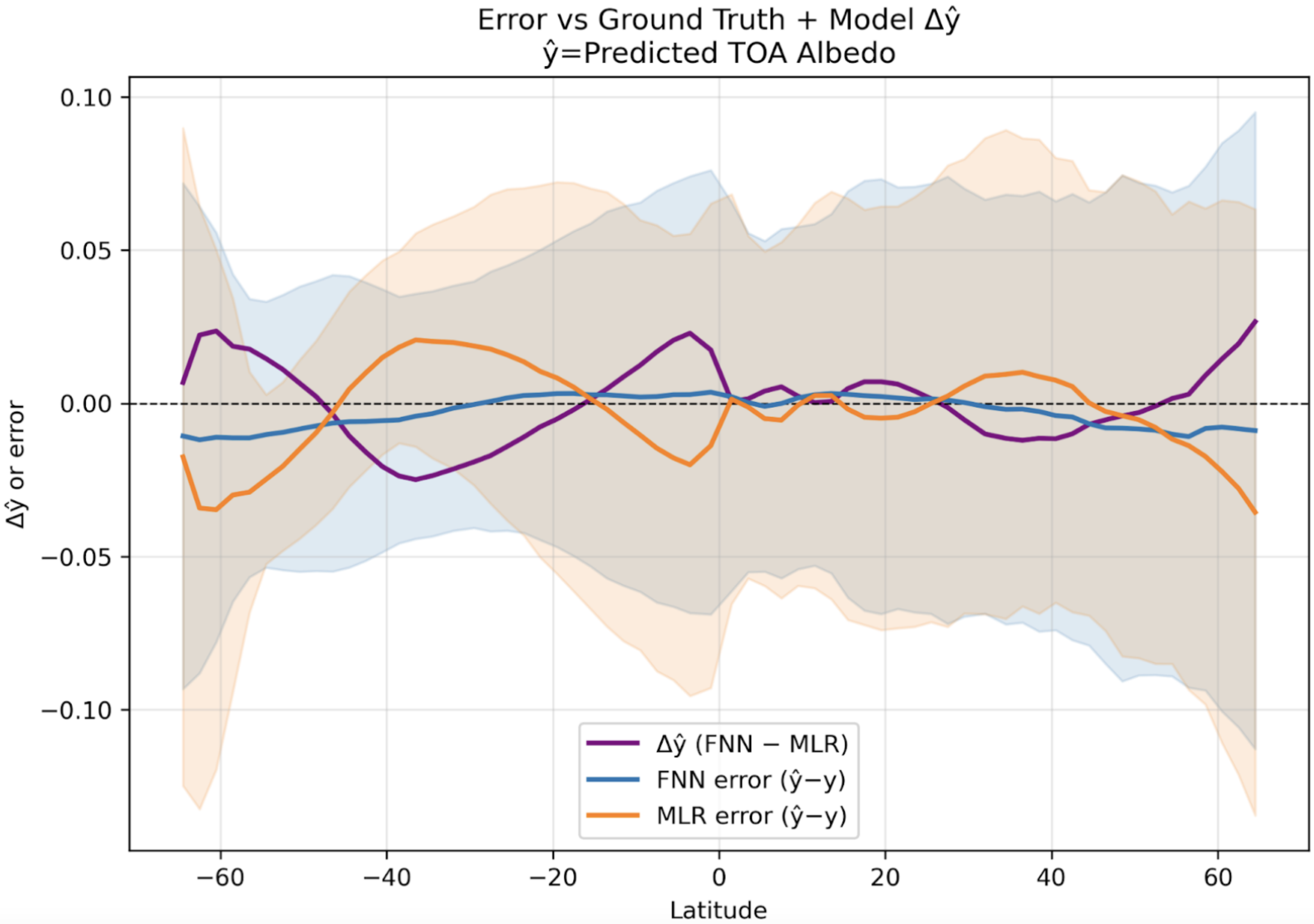

Supplemental Figure 6. Out-of-sample error meridional error structure for the FFNN and MLR models.

Additionally, we note that there is a higher-level reason to consider the "latitude" and "northern hemisphere flag" importance finding of the FFNN SHAP. FFNNs capture spatial patterns in data (e.g., 4) while the representation of spatial patterns in MLR and other regression methods must be explicit and chosen *a priori*. Because there are likely spatial teleconnections contributing to the TOA albedo phenomena, the ability of a data model to capture spatial patterns in the data and use them for prediction is paramount.

It is, however, reasonable to note that there are other models of reduced complexity and increased explainability compared to an FFNN, and also models of greater complexity and decreased explainability compared to an FFNN, which may point to yet other and different research directions the MLR and FFNN. We argue that the performance of a model against out-of-sample data will be maximized at a certain level of model complexity, simply because there is a risk of overfitting to the training data and that training data only contain a finite amount of information on the phenomena of interest and the phenomena are of finite complexity.

To the extent that more complex models achieve greater predictive skill (i.e., improving on the error in the FFNN shown in Supplementary Figure 7) with out-of-sample data (and thus are not overfitting), their use should be strongly considered. The skill of ever-more complex models with out-of-sample predictors and predictands will eventually degrade as these models become overfitted. At that point, models of excessive complexity will not generalize well, since their performance is ultimately limited by the amount of information contained in the predictors about the predictand.

There are well-established metrics that seek to balance data model performance with out-of-sample predictands with the data model's complexity. This is relevant for the MLR vs. FFNN example presented here, because, as shown in Supplementary Figure 7, the number of parameters in each of these data models is highly dissimilar (16 for MLR vs. 13409 for the FFNN). Akaike Information Criterion (AIC) is one widely-utilized metric that seeks to strike a balance between a model's goodness of fit and its complexity from an information-theoretic perspective[5]. Briefly, AIC is defined as the following:

$$AIC = 2k - 2ln(\hat{L}) \quad (1)$$

where $\hat{L}$ is the maximized value of the likelihood function for the data model and k is the number of estimated parameters in the data model. A more negative value of AIC indicates a more optimal data model in terms of skill and avoiding overfitting for prediction. Supplementary Figure 9 shows AIC values MLR and FFNN with varying levels of ablation, and shows that FFNN is more optimal for albedo prediction.

Another widely-utilized metric for balancing goodness of fit and complexity is the Bayesian Information Criterion (BIC), which is defined as

$$BIC = kln(n) - 2ln(\hat{L}) \quad (2)$$

where n is the number of data points. We compare and contrast BIC and AIC because BIC penalizes complexity more than AIC[6,7]. For both of these metrics, a negative value indicates a superior model. Also, (BIC) is generally more useful in selecting an accurate model, while AIC is more appropriate in finding the best model for predicting future observations[8]. Figure 7 shows that for both the AIC and BIC metrics, the FFNN model outperforms the MLR model and both the AIC and BIC metrics are similar for the FFNN data model. While there are certainly other metrics that balance data model performance and complexity, we have also cross-validated the FFNN in order to further establish confidence in its performance and to guard against overfitting[9,10].

For all of these data models, including ones of optimal complexity, the SHAP analysis can be helpful for aiding explainability. Though SHAP is by no means the only method for estimating feature importance and understanding data models, it is widely-utilized and has a strong information-theoretic basis. Nevertheless, SHAP will not provide deep insights into underlying physical principles, but it does highlight feature importance and it can, therefore, provide research guidance as to the processes that are important and unimportant for explaining albedo phenomena. If the SHAP analysis of models that perform better with out-of-sample data points in a different direction (in terms of which features are most important for predicting albedo) from the direction of models with inferior performance with out-of-sample data, we argue that it is warranted to prioritize the research directions suggested by the relative SHAP feature importance values in the better-performing model. Still, the SHAP values are merely suggestions for formulating observational tests of the explanatory hypotheses of the albedo phenomena.

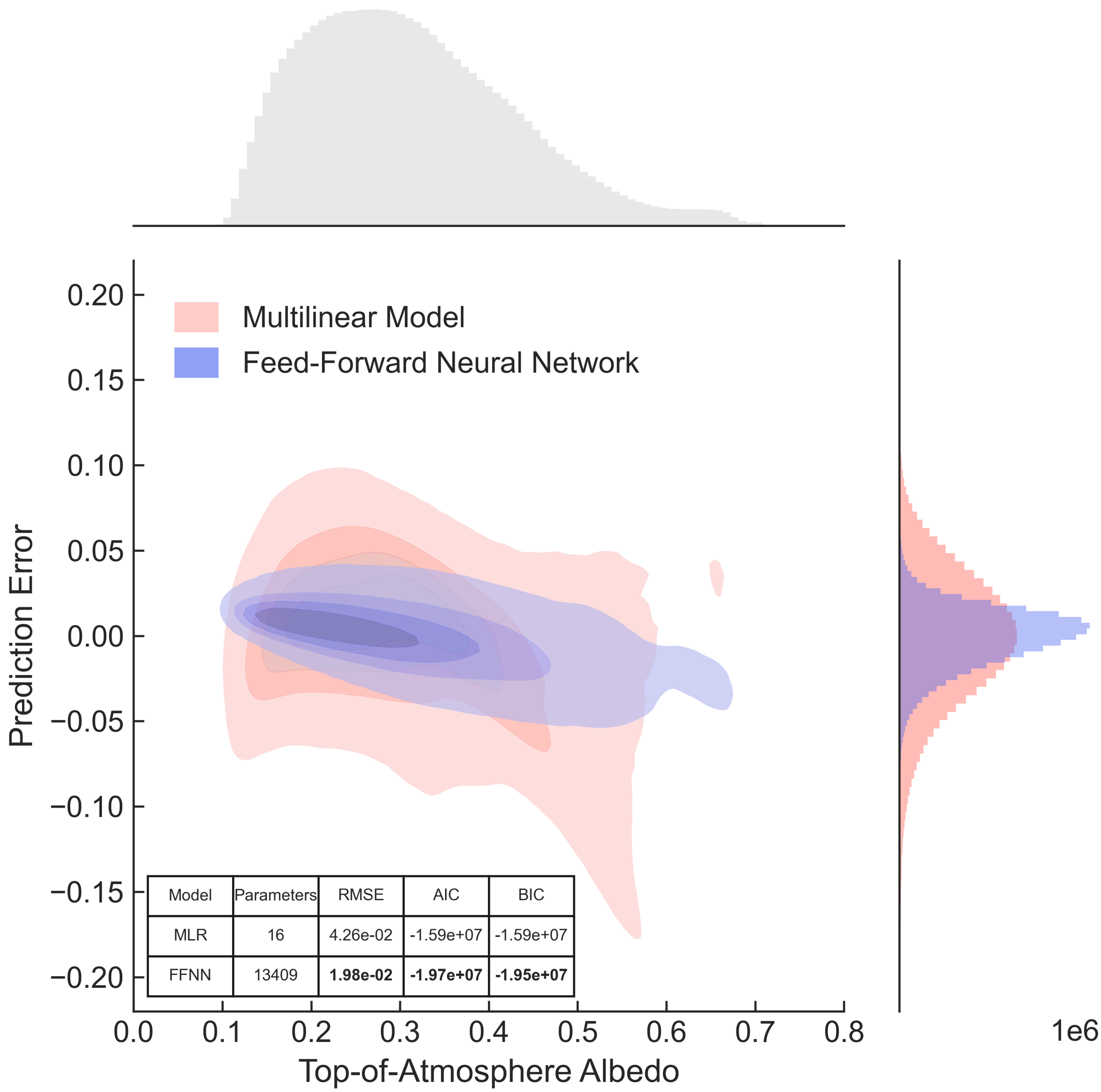

Model	Parameters	RMSE	AIC	BIC
MLR	16	4.26e-02	-1.59e+07	-1.59e+07
FFNN	13409	**1.98e-02**	**-1.97e+07**	**-1.95e+07**

Supplemental Figure 7. Out-of-sample (5-fold Cross Validation) TOA Albedo prediction errors for Multilinear Regression (MLR) and 4 Layer Feed-Forward Neural Network (FFNN) models. The gray PDF at top of plot indicates distribution of EBAF-observed TOA albedo and the red and blue PDFs on the right of plot indicate the distributions of prediction errors for all albedo values with with MLR and FFNN models, respectively. The number of model parameters, root-mean squared error (RMSE), the Akaike Information Criterion (AIC) and the Bayesian Information Criterion (BIC) for each data model are shown on the inset table. The better score of the two models is highlighted in bold.

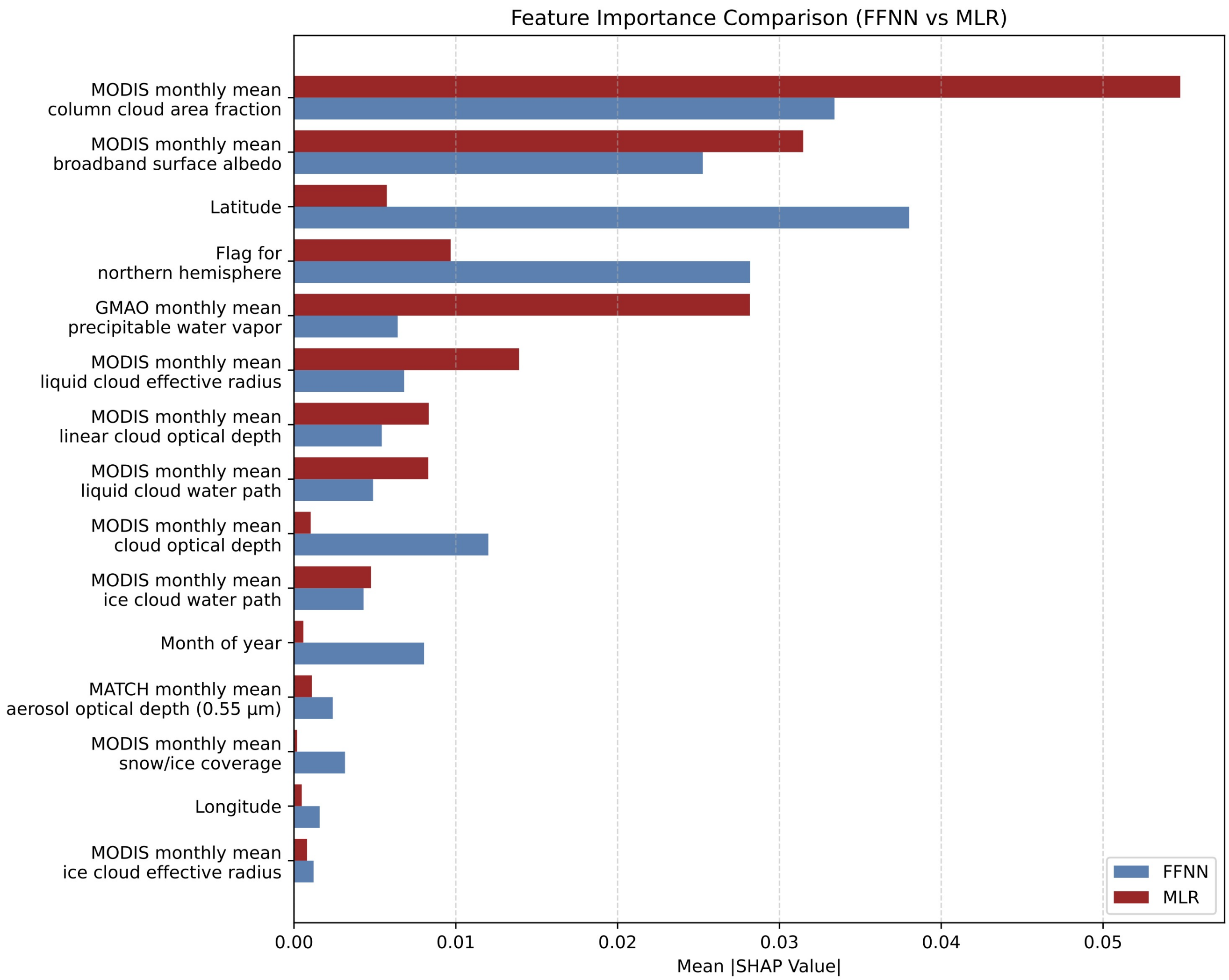

Supplemental Figure 8. The comparative feature values (Mean of the absolute values of the SHAP values) between the MLR and FFNN data models for predicting TOA albedo.

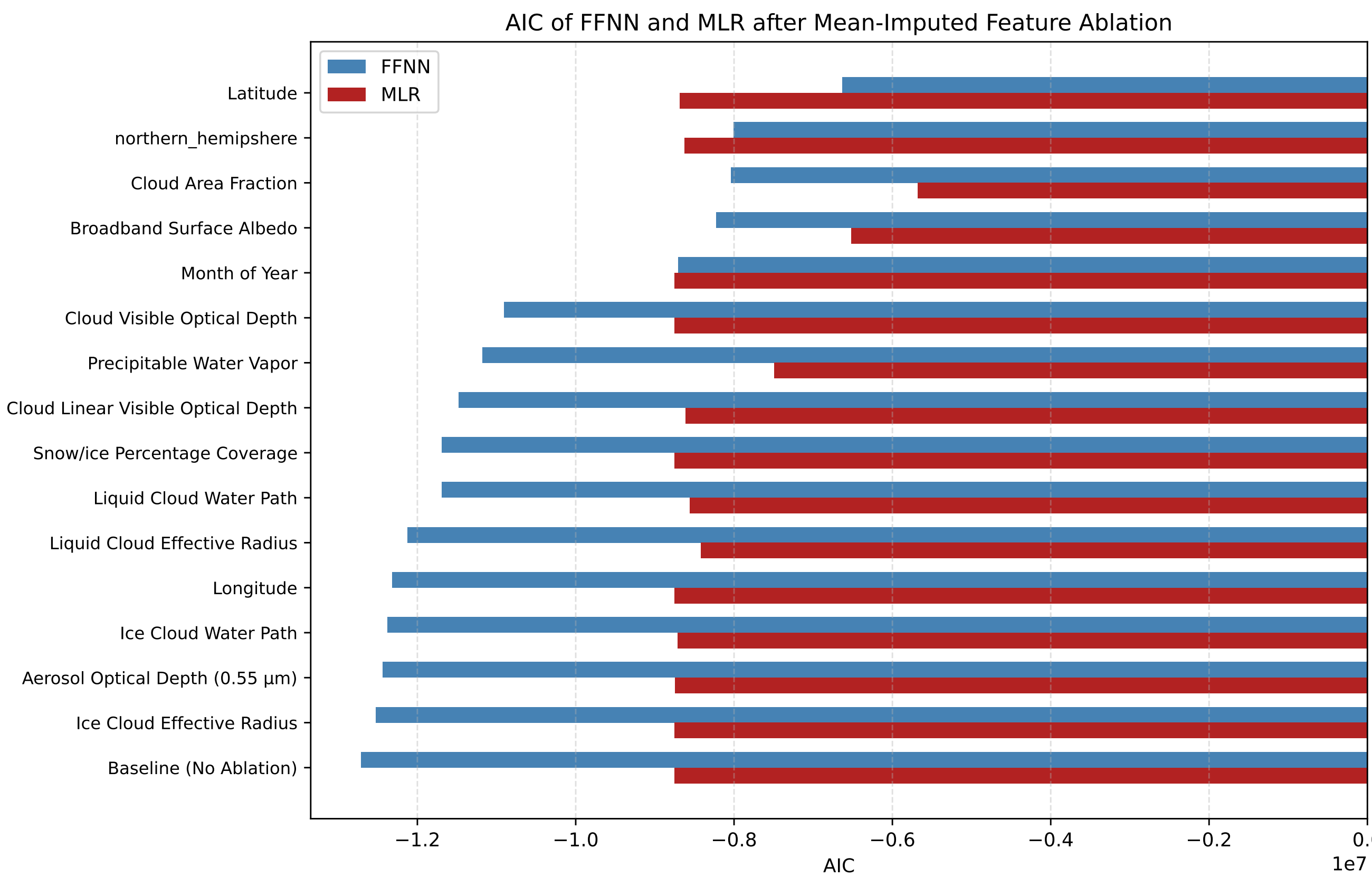

Supplemental Figure 9. Akaike Information Criterion (AIC) for the MLR and FFNN data models with and without predictor variable ablation

Supplementary References

1. Allan, R. *et al.* Changes in global net radiative imbalance 1985-2012. *Geophys. Res. Lett.* **41(15)**, 5588–5597 (2014).
2. Liu, C. & Allan, R. Reconstructions of the radiation fluxes at the top of atmosphere and net surface energy flux: Deep-c version 5.0. *Univ. Read.* (2022).
3. Dudok de Wit, T., Kopp, G., Fröhlich, C. & Schöll, M. Methodology to create a new total solar irradiance record: Making a composite out of multiple data records. *Geophys. Res. Lett.* **44**, 1196–1203 (2017).
4. Lowe, D. & Tipping, M. Feed-forward neural networks and topographic mappings for exploratory data analysis. *Neural. Comput. Applic.* **4**, 83–95 (1996).
5. Akaike, H. A new look at the statistical model identification. *IEEE Transactions on Autom. Control.* **19(6)**, 716–723 (1974).
6. Schwarz, G. Estimating the dimension of a model. *Ann. Stat.* **6(2)**, 461–464 (1978).
7. Stoica, P. & Selen, Y. Model-order selection: a review of information criterion rules. *IEEE Signal Process. Mag.* **21(4)**, 36–47 (2004).
8. Chakrabarti, A. & Ghosh, J. Aic, bic and recent advances in model selection. *Philos. Stat.* 583–605 (2011).
9. Zhang, Y. & Yang, Y. Cross-validation for selecting a model selection procedure. *J. Econom.* **187(1)**, 95–112 (2015).
10. Mei, X. & Smith, P. A comparison of in-sample and out-of-sample model selection approaches for artificial neural network (ann) daily streamflow simulation. *Water* **13(18)**, 2525 (2021).
